# Fermatean hesitant fuzzy rough aggregation operators and their applications in multiple criteria group decision-making

**DOI:** 10.1038/s41598-023-28722-w

**Published:** 2023-04-24

**Authors:** Attaullah, Noor Rehman, Asghar Khan, Gustavo Santos-García

**Affiliations:** 1 Department of Mathematics, Abdul Wali Khan University, Mardan, 23200 KP Pakistan; 2 Department of Mathematics and Statistics, Bacha Khan University Charsadda, Charsadda, 24460 KP Pakistan; 3 Instituto Multidisciplinar de la Empresa (IME), Universidad de Salamanca, 37007 Salamanca, Spain

**Keywords:** Mathematics and computing, Pure mathematics

## Abstract

The precise selection of suppliers to fulfill production requirements is a fundamental component of all manufacturing and process industries. Due to the increasing consumption levels, green supplier selection (GSS) has been one of the most important issues for environmental preservation and sustainable growth. The present work aims to develop a technique based on Fermatean hesitant fuzzy rough set (FHFRS), a robust fusion of Fermatean fuzzy set, hesitant fuzzy set, and rough set for GSS in the process industry. On the basis of the operational rules of FHFRS, a list of innovative Fermatean hesitant fuzzy rough weighted averaging operators has been established. Further, several intriguing features of the proposed operators are highlighted. To cope with the ambiguity and incompleteness of real-world decision-making (DM) challenges, a DM algorithm has been developed. To illustrate the applicability of the methodology, a numerical example for the chemical processing industry is presented to determine the optimum supplier. The empirical findings suggest that the model has a significant application of scalability for GSS in the process industry. Finally, the improved FHFR-VIKOR and TOPSIS approaches are employed to validate the proposed technique. The results demonstrate that the suggested DM approach is practicable, accessible, and beneficial for addressing uncertainty in DM problems.

## Introduction

The supply chain is a system of procedures to acquire crude materials, transform them into substantial and final product, and shipped to the customer. It comprises all relationships between suppliers and consumers. The objective of supply chain management (SCM) is to improve the physical and information flow that is exchanged across all stakeholder involved in the supply chain^1^. Sustainable supply chains may promote a long-term efficient relationship throughout the diverse firms. Supplier selection is the process through which firms locate, analyse, and negotiate with suppliers^2^. In the modern era of internet-based corporate environments, the significance of SCM and supplier selection has been elevated, and firms pay special attention to the investigation and selection of potential sources of supply. Whenever a supplier becomes a partner, the interaction between the supplier and buyer will have a significant impact on the rivalry integrity of the entire SCM. As the majority of firms devote a substantial portion of their income on procurement, the supplier selection procedure has become one of the most significant features of developing an efficient SCM system^3^. Whenever organisations become more reliant on suppliers, the direct and indirect repercussions of terrible supplier selection decisions will intensify^4^. The choosing of a supplier is a difficult DM strategy. Before firms made judgments almost completely based on expense and variety, the majority of modern investigators believe that the arrangement of features should address not only technological and economic needs, but also social and environmental requirements^5^. Ho et al.^6^ suggested that management system be utilized to accurately assess the supplier selection. Conventional supplier selection approaches emphasise the provider’s economic and technical efficiency while neglecting its sustainability performance. Today, enterprises should evaluate the environmental sensitivity of their consumption and supply that they minimize their influence on the environment^7^. In combination with appropriate factors such as price and quality, the green challenges may play a significant influence on procurement and provide key environmental variables that can be employed to evaluate the suppliers. The emergence of manufacturing, green SCM may be seen as a significant approach for all buyers and suppliers. To improve the supply chain’s value, the current competitive industries have prompted businesses to connect environmental concerns with other essential considerations (cost, quality, service level, etc.). Therefore, hiring green suppliers to decrease procurement risk is one of the most significant DM challenges.

Uncertainty is a repercussion of both the objective world’s complexity and the scarcity of human understanding. Uncertainty is a difficult-to-describe character trait, and the majority of its expressions are unpredictable and fuzzy. To correctly describe the ambiguous information in real issues, several novel theories and techniques have been developed see for^8–15^ more details. Among these approaches, fuzzy set (FS) theory has garnered considerable interest. Zadeh^14^ introduced FS theory, which is used to express the fuzzy and absurd information of objective items. FS theory describes theoretical framework for the interpretation of imprecise information and enables the transformation of DM information from the linguistics variable to the numerical variable. The FS theory has the ability to develop a novel approach to exhibit ambiguous and conflicting information while addressing the problems with conventional DM processing information. The study on FS theory has yielded promising discoveries and has been significantly employed in numerous diverse fields. Whenever the complexity of a DM problem grows, classical FS theory is unable to adequately represent the uncertain information in the problem^16^. For this issue, several researchers have provided the enhanced forms of classical FSs from a variety of perspectives, including intuitionistic FSs (IFSs)^17^, Pythagorean FSs (PFSs)^18^, hesitant FSs (HFSs)^19^, and rough sets (RSs)^20^. The efficacy of many concepts of generalised fuzzy sets served as the inspiration for the notion of MCGDM, which has been the subject of several investigations (see^21–24^ for more information). Wu et al.^25^ suggested a multi-criteria sequential calibration and uncertainty analysis technique for improving the efficiency and performance of high-reliability hydrological modelling. Two case studies were undertaken in comparison with two other approaches, sequential uncertainty fitting algorithm and generalised likelihood uncertainty estimation, to assess the performance and practicality of the suggested method. Wang et al.^26^ introduced a hybrid MCDM framework that combines the spherical fuzzy analytical hierarchy process (SF-AHP) with weighted aggregated sum product assessment (WASPAS). The optimum site for an offshore wind power station (OWPS) was determined using a decision framework based on the spherical fuzzy set approach. Basset et al.^27^ explored an axiomatic design to expand MCDM in the neutrosophic environment as a significant contribution to select optimal computed tomography equipment. They introduced a new linguistic scale based on single-valued triangular neutrosophic numbers for assessing criteria and alternatives. Limberger et al.^28^ established the first numerical model to anticipate the seismic wave field generated by wind farms as well as simulate the complicated effects of wave field interferences, surface topography, and attenuation. This proposed modelling technique can accurately estimate the effects of several wind turbines on ground motion recordings, providing critical information to guide decision-making prior to wind farm implementation. Stańczyk et al.^29^ designed and presented a method for predicting water demand based on a linear regression model integrated with evolutionary techniques to extract weekly seasonality. Eseoglu et al.^30^ designed a novel fuzzy framework for technology selection of sustainable waste water treatment plants in emerging metropolitan areas based on TODIM methodology.

The rough set theory, established by Pawlak^20^ in the 1980s, is a robust branch of artificial intelligence with applications in many areas of data mining^31–33^, attributes and feature identification^34–36^, and data prediction^37,38^. FSs theory can be combined with RS theory to handle information with continuous features and identify information discrepancies. Owing to the fact that the fuzzy RS approach is an effective technique for evaluating inconsistent and imprecise information, it has shown to be valuable in a wide variety of application domains. Numerous scholars have applied RS theory to scientific disciplines including industrial applications^39^, pharmaceutical, health, and bio-informatics^40–42^, traffic and transportation^43,44^, environmental sciences^45,46^, environmental engineering and protection of the environment management^47^, security scientific method^48^, and aerospace, space technology, and military control^49^.

Since the development of RS theory, numerous significant generalisations of RS in diverse directions have been established^50–55^. In more recent years, RS approximations have been introduced to IF sets^56,57^. They subsequently introduced the notion of IF rough sets, in which both the lower approximations (LA) and upper approximations (UA) are IF sets. Feng et al.^58,59^ introduced the innovative ideas of soft RS, soft set, and rough sets to examine certain information system characteristics. Zhang et al.^60,61^ established the IF soft RS and interval-valued hesitant fuzzy rough approximation operators. Zhan and Alcantud^62^ developed an overview AOPs, and their applicability to the DM problem. Pamucar^63^ presented a geometric Dombi Bonferroni mean operator using interval grey numbers and discussed their application in DM. Ali et al.^64^ established Einstein geometric AOPs with a unique complex interval-valued Pythagorean FSs for use in green SCM. Motivated by the robust application of AOPs in DM, in this article, the present authors introduce an innovative notation of Fermatean hesitant fuzzy RSs (FHFRSs), which is a hybrid structure of RSs and Fermatean hesitant FSs, having piqued their curiosity for its capacity to handle ambiguous and imprecise information. According to the existing literature, AOPs are essential in DM because they enable information from several sources to be aggregated into a single number^65–72^. The development of AOps FHFSs hybridization with RSs is not observed in the existing research. Pursuant to this motivation, we develop a list of algebraic AOPs for FHFR information, including FHFR weighted averaging (WA), order weighted averaging (OWA, and hybrid weighted averaging (HWA, under the algebraic t-norm and t-conorm, and explore their significant features in detail. Furthermore, a case study of a real-world DM problem in GSS for the chemical process industry based on a new concept of FHFRSs is considered, economic and environmental aspects are appropriately evaluated for GSS. To demonstrate the validity of the suggested DM technique, an enhanced FHFR-VIKOR method is employed.

The remaining of this article is organised as follows: Section Basic terminologies contains a concise review of the fundamental and the innovative concept of FHFRSs. The FHFR AOPs are described in Section The Fermatean hesitant fuzzy rough aggregation operators. Methodologies for MCGDM are discussed in Section Multi-attribute decision making framework. The implementation of the GSS and assessment of DM model in the chemical processing industry is presented in Section Numerical implementation of the MCGDM framework. In Section The comparative evaluation, the suggested technique is verified via the use of the improved VIKOR and TOPSIS schemes based on FHFRSs. Section Concluding remarks and future recommendations concludes with a description of the results and future recommendations.

## Basic terminologies

This section presents the following terms: intuitionistic FSs (IFSs), Pythagorean FSs (PFSs), Pythagorean hesitant FSs (PHFSs), rough sets (RSs), Fermatean fuzzy RSs (FFRSs) and Fermatean hesitant fuzzy RSs (FHFRSs).

### Definition 2.1

^17^An IFS $\mathcal {F}$ over a universal set $\mathcal {U}$ is described as:

$$\begin{aligned} \mathcal {F} =\left\{ \langle \vartheta ,\zeta _{\mathcal {F} }(\vartheta ),\mathcal {J} _{\mathcal {F} }(\vartheta )\rangle |\vartheta \in \mathcal {U} \right\} , \end{aligned}$$

for each $\vartheta \in \mathcal {F}$ the functions $\zeta _{\mathcal {F} }:\mathcal {U} \rightarrow [0$ ,1] and $\mathcal {J} _{\mathcal {F} }:\mathcal {U} \rightarrow [0,$ 1] denote the positive membership grade (PMG) and negative membership grade (NMG) respectively subject to the condition that $0\le \zeta _{\mathcal {F} }(\vartheta )+\mathcal {J} _{\mathcal {F} }(\vartheta )\le 1.$

### Definition 2.2

^73^A PFS ${\mathcal {T}}$ over a universal set $\mathcal {U}$ is described as follows:

$$\begin{aligned} {\mathcal {T}} =\left\{ \langle \vartheta ,\zeta _{{\mathcal {T}} }(\vartheta ),\mathcal {J} _{{\mathcal {T}} }(\vartheta )\rangle |\vartheta \in \mathcal {U} \right\} \end{aligned}$$

for all $\vartheta \in {\mathcal {T}}$ the functions $\zeta _{{\mathcal {T}} }:\mathcal {U} \rightarrow [0,$ 1] and $\mathcal {J} _{{\mathcal {T}} }:\mathcal {U} \rightarrow [0,$ 1] denote the PMG and NMG respectively subject to the condition that $(\mathcal {J} _{{\mathcal {T}} }(\vartheta ))^{2}+(\zeta _{{\mathcal {T}} }(\vartheta ))^{2}\le 1.$

### Definition 2.3

**Figure 1:**
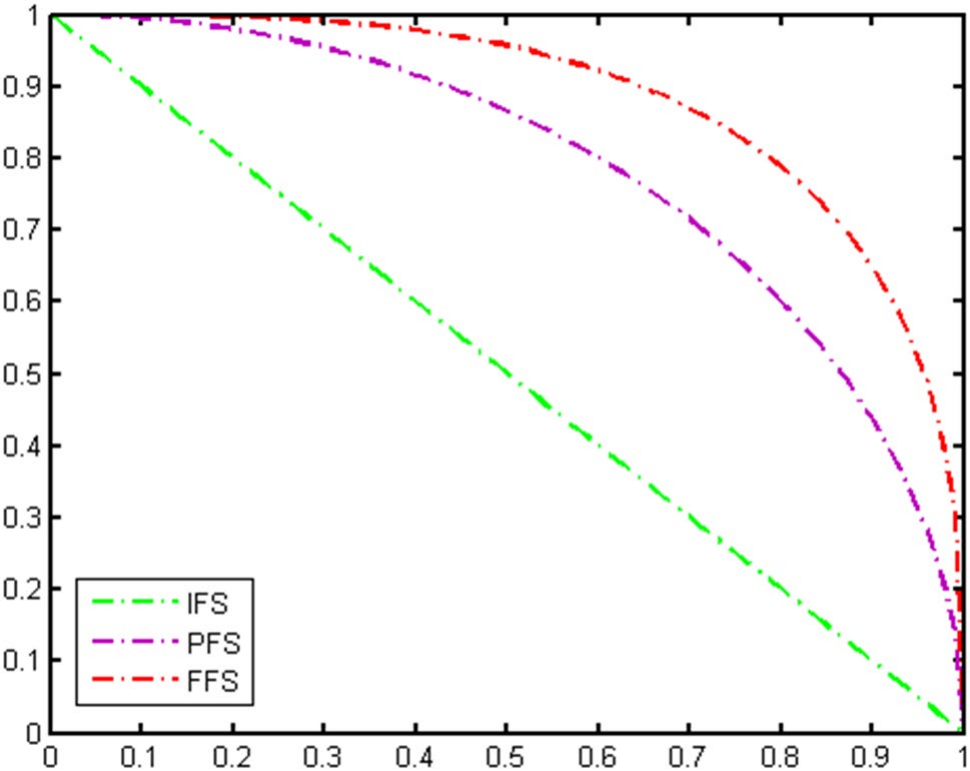
The pictorial representation of the IFS, PFS and FFS space.

^73^A Fermatean FS ${\mathcal {T}}$ over a universal set $\mathcal {U}$ is defined as follows:

$$\begin{aligned} {\mathcal {T}} =\left\{ \langle \vartheta ,\zeta _{{\mathcal {T}} }(\vartheta ),\mathcal {J} _{{\mathcal {T}} }(\vartheta )\rangle |\vartheta \in \mathcal {U} \right\} \end{aligned}$$

for all $\vartheta \in {\mathcal {T}}$ the functions $\zeta _{{\mathcal {T}} }:\mathcal {U} \rightarrow [0,$ 1] and $\mathcal {J} _{{\mathcal {T}} }:\mathcal {U} \rightarrow [0,$ 1] symbolize the PMG and NMG respectively subject to the condition that $(\mathcal {J} _{{\mathcal {T}} }(\vartheta ))^{3}+(\zeta _{{\mathcal {T}} }(\vartheta ))^{3}\le 1.$ The pictorial depiction of the IFS, PFS, and FFS is illustrated in Fig. 1.

### Definition 2.4

^74^A Fermatean hesitant fuzzy set (FHFS) $\mathcal {H}$ over a universal set $\mathcal {U}$ is defined as follows:

$$\begin{aligned} \mathcal {H} =\{\left\langle \vartheta ,\zeta _{h_{\mathcal {H} }}(\vartheta ),\mathcal {J} _{h_{\mathcal {H} }}(\vartheta )\right\rangle |\vartheta \in \mathcal {U} \}, \end{aligned}$$

where $\zeta _{h_{\mathcal {H} }}(x)$ and $\mathcal {J} _{h_{\mathcal {H} }}(\vartheta )$ are sets of some values in $\left[ 0,1\right]$ and show the PMG and NMG respectively subject to the conditions: $\forall ~\vartheta \in \mathcal {U} ,$ $\forall \mu _{\mathcal {H} }(x)\in \zeta _{h_{\mathcal {H} }}(\vartheta ),$ $\forall ~{\mathcal {V}} _{\mathcal {H} }(\vartheta )\in \mathcal {J} _{h_{\mathcal {H} }}(\vartheta )$ with $\left( \max \left( \zeta _{h_{\mathcal {H} }}(\vartheta )\right) \right) ^{3}+\left( \min \left( \mathcal {J} _{h_{\mathcal {H} }}(\vartheta )\right) \right) ^{3}\le 1$ and $\left( \min \left( \zeta _{h_{\mathcal {H} }}(\vartheta )\right) \right) ^{3}+\left( \max \left( \mathcal {J} _{h_{\mathcal {H} }}(\vartheta )\right) \right) ^{3}\le 1.$ To put it simply, we will utilize a pair $\mathcal {H}=$ $(\zeta _{h_{\mathcal {H} }},\mathcal {J} _{h_{\mathcal {H} }})$ to refer to the FHF number (FHFN).

### Definition 2.5

^74^Let $\mathcal {R} _{1}=(\zeta _{h_{\mathcal {R} _{1}}},\mathcal {J} _{h_{\mathcal {R} _{1}}})$ and $\mathcal {R} _{2}=(\zeta _{h_{\mathcal {R} _{2}}},\mathcal {J} _{h_{\mathcal {R} _{2}}})$ be two FHFNs. Then the fundamental set-theoretic operations are:

1. $$\mathcal {R} _{1}\cup \mathcal {R} _{2}=\left\{ \underset{_{\underset{\mu _{2}\in \zeta _{h_{\mathcal {R} _{2}}}}{\mu _{1}\in \zeta _{h_{\mathcal {R} _{1}}}} }}{\bigcup }\max \left( \mu _{1},\mu _{2}\right) ,\underset{_{\underset{{\mathcal {V}} _{2}\in \mathcal {J} _{h_{\mathcal {R} _{2}}}}{{\mathcal {V}} _{1}\in \mathcal {J} _{h_{\mathcal {R} _{1}}}} }}{\bigcup }\min \left( {\mathcal {V}} _{1},{\mathcal {V}} _{2}\right) \right\} ;$$
2. $$\mathcal {R} _{1}\cap \mathcal {R} _{2}=\left\{ \underset{_{\underset{\mu _{2}\in \zeta _{h_{\mathcal {R} _{2}}}}{\mu _{1}\in \zeta _{h_{\mathcal {R} _{1}}}} }}{\bigcup }\min \left( \mu _{1},\mu _{2}\right) ,\underset{_{\underset{{\mathcal {V}} _{2}\in \mathcal {J} _{h_{\mathcal {R} _{2}}}}{{\mathcal {V}} _{1}\in \mathcal {J} _{h_{\mathcal {R} _{1}}}} }}{\bigcup }\max \left( {\mathcal {V}} _{1},{\mathcal {V}} _{2}\right) \right\} ;$$
3. $$\mathcal {R} _{1}^{c}=\left\{ \mathcal {J} _{h_{\mathcal {R} _{1}}},\zeta _{h_{\mathcal {R} _{1}}}\right\} .$$

### Definition 2.6

^74^Let $\mathcal {R} _{1}=(\zeta _{h_{\mathcal {R} _{1}}},\mathcal {J} _{h_{\mathcal {R} _{1}}})$ and $\mathcal {R} _{2}=(\zeta _{h_{\mathcal {R} _{2}}},\mathcal {J} _{h_{\mathcal {R} _{2}}})$ be two FHFNs and $\gamma >0$ be any positive real number. The basic laws are formulated in the following way:

1. $$\mathcal {R} _{1}\oplus \mathcal {R} _{2}=\left\{ \underset{_{\underset{ \mu _{2}\in \zeta _{h_{\mathcal {R} _{2}}}}{\mu _{1}\in \zeta _{h_{\mathcal {R} _{1}}}}}}{\bigcup }\left\{ \root 3 \of {\mu _{1}^{3}+\mu _{2}^{3}-\mu _{1}^{3}\mu _{2}^{3}}\right\} ,\underset{_{\underset{{\mathcal {V}} _{2}\in \mathcal {J} _{h_{\mathcal {R} _{2}}}}{{\mathcal {V}} _{1}\in \mathcal {J} _{h_{\mathcal {R} _{1}}}}}}{\bigcup }\left\{ {\mathcal {V}} _{1}\cdot {\mathcal {V}} _{2}\right\} \right\} ;$$
2. $$\mathcal {R} _{1}\otimes \mathcal {R} _{2}=\left\{ \underset{_{\underset{ \mu _{2}\in \zeta _{h_{\mathcal {R} _{2}}}}{\mu _{1}\in \zeta _{h_{\mathcal {R} _{1}}}}}}{\bigcup }\left\{ \mu _{1}\cdot \mu _{2}\right\} ,\underset{_{ \underset{{\mathcal {V}} _{2}\in \mathcal {J} _{h_{\mathcal {R} _{2}}}}{{\mathcal {V}} _{1}\in \mathcal {J} _{h_{\mathcal {R} _{1}}}}}}{\bigcup }\left\{ \root 3 \of {{\mathcal {V}} _{1}^{3}+{\mathcal {V}} _{2}^{3}-{\mathcal {V}} _{1}^{3}{\mathcal {V}} _{2}^{3}}\right\} \right\} ;$$
3. $$\varsigma \mathcal {R} _{1}=\left\{ \underset{_{\mu _{1}\in \zeta _{h_{\mathcal {R} _{1}}}}}{\bigcup }\left\{ \root 3 \of {1-(1-\mu _{1}^{3})^{\varsigma }}\right\} ,\ \underset{{\mathcal {V}} _{1}\in \mathcal {J} _{h_{\mathcal {R} _{1}}}}{\bigcup }\left\{ {\mathcal {V}} _{1}^{\varsigma }\right\} \right\} ;$$(4) $$\mathcal {R} _{1}^{\varsigma }=\left\{ \underset{_{\mu _{1}\in \zeta _{h_{\mathcal {R} _{1}}}}}{\bigcup }\left\{ \mu _{1}^{\varsigma }\right\} , \underset{{\mathcal {V}} _{1}\in \mathcal {J} _{h_{\mathcal {R} _{1}}}}{\bigcup }\left\{ \root 3 \of {1-(1-{\mathcal {V}} _{1}^{3})^{\varsigma }}\right\} \right\} .$$

### Definition 2.7

^20^Let $\mathcal {Z} \subseteq \mathcal {U} \times \mathcal {U}$ be a (crisp) relation and $\mathcal {U}$ be a universal set. Then

1. $\eth$ is known to be reflexive if $(\jmath ,\jmath )\in \eth ,$ for all $\jmath \in \mathcal {U} ;$
2. $\eth$ is known to be symmetric if for all $\jmath ,\varpi \in \mathcal {U} ,$ $(\jmath ,\varpi )\in \eth$ then $(\varpi ,\jmath )\in \eth ;$
3. $\eth$ is known to be transitive if for all $\jmath ,\varpi ,\varphi \in \mathcal {U} ,(\jmath ,\varpi )\in \mathcal {U}$ and $(\varpi ,\varphi )\in \eth$ then $(\jmath ,\varphi )\in \eth .$

### Definition 2.8

^20^Let $\eth$ be any relation on a universal set $\mathcal {U} .$ Characterize a mapping $\eth ^{*}:\mathcal {U} \rightarrow M(\mathcal {U} )$ by $\eth ^{*}(\jmath )=\{\varpi \in \mathcal {U} |(\jmath ,\varpi )\in \eth \},$ for $\jmath \in \mathcal {U}$ where $\eth ^{*}(\jmath )$ is called a successor neighborhood of the element $\jmath$ w.r.t. relation $\eth .$ The pair $\left( \mathcal {U} ,\eth \right)$ is called crisp approximation space. Now for any set $\gimel \subseteq \mathcal {U},$ the LA and UA of $\gimel$ w.r.t. approximations space $\left( \mathcal {U} ,\eth \right)$ is described as follows:

$$\begin{aligned} \underline{\eth }(\gimel )= & {} \{\jmath \in \mathcal {U} |\eth ^{*}(\jmath )\subseteq \gimel \}; \\ \overline{\eth }(\gimel )= & {} \{\jmath \in \mathcal {U} |\eth ^{*}(\jmath )\cap \gimel \ne \phi \}. \end{aligned}$$

The pair $\left( \underline{\eth }(\gimel ),\overline{\eth }(\gimel )\right)$ is called RS and both $\underline{\eth }(\gimel ), \overline{\eth }(\gimel ):M(\mathcal {U} )\rightarrow M(\mathcal {U} )$ are LA and UA operators.

### Definition 2.9

^75^Let $\eth \in IFS(\mathcal {U} \times \mathcal {U} )$ be an IF relation and $\mathcal {U}$ be the universal set. Then

1. $\eth$ is known to be reflexive if $\mu _{\eth }(\jmath ,\jmath )=1$ and ${\mathcal {V}} _{\eth }(\jmath ,\jmath )=0,\forall \jmath \in \mathcal {U} ;$
2. $\eth$ is known to be symmetric if $\ \forall (\jmath , \varpi )\in \mathcal {U} \times \mathcal {U} ,$ $\mu _{\eth }(\jmath ,\varpi )=\mu _{\eth }(\varpi ,\jmath )$ and ${\mathcal {V}} _{\eth }(\jmath ,\varpi )={\mathcal {V}} _{\eth }(\varpi ,\jmath );$
3. $\eth$ is known to be transitive if $\forall (\jmath ,\varpi )\in \mathcal {U} \times \mathcal {U} ,$
  $$\begin{aligned} \mu _{\eth }(\jmath ,\varphi )\ge \bigvee \nolimits _{\varpi \in \mathcal {U} }\left[ \mu _{\eth }(\jmath ,\varpi )\wedge \mu _{\eth }(\varpi ,\varphi )\right] ; \end{aligned}$$
  and
  $$\begin{aligned} {\mathcal {V}} _{\eth }(\jmath ,\varphi )=\bigwedge \nolimits _{\varpi \in \mathcal {U} }\left[ {\mathcal {V}} _{\eth }(\jmath ,\varpi )\wedge {\mathcal {V}} _{\eth }(\varpi ,\varphi )\right] . \end{aligned}$$

### Definition 2.10

Let $\mathcal {U}$ be any fixed set. Then any $\eth \in FFS(\mathcal {U} \times \mathcal {U} )$ is called Fermatean fuzzy relation. The pair $\left( \mathcal {U} \times \eth \right)$ is said to be Fermatean approximation space. Now for any $\gimel \subseteq FFS(\mathcal {U} )$, the LA and UA of $\gimel$ w.r.t. Fermatean fuzzy approximation space $\left( \mathcal {U} ,\eth \right)$ are two FFSs, which are symbolised by $\underline{\eth }(\gimel )$ and $\overline{\eth }(\gimel )$ and described below as:

$$\begin{aligned} \underline{\eth }(\gimel )= & {} \{\left\langle \jmath ,\mu _{\underline{\eth } (\gimel )}(\jmath ),{\mathcal {V}} _{\underline{\eth }(\gimel )}(\jmath )\right\rangle |\jmath \in \mathcal {U} \}; \\ \overline{\eth }(\gimel )= & {} \{\left\langle \jmath ,\mu _{\overline{\eth } (\gimel )}(\jmath ),{\mathcal {V}} _{\overline{\eth }(\gimel )}(\jmath )\right\rangle |\jmath \in \mathcal {U} \}; \end{aligned}$$

where

$$\begin{aligned} \mu _{\overline{\eth }(\gimel )}(\jmath )= & {} \underset{g\in \mathcal {U} }{ \bigvee }[\mu _{\eth }(\jmath ,g)\bigvee \mu _{\gimel }(g)]; \\ {\mathcal {V}} _{\overline{\eth }(\gimel )}(\jmath )= & {} \underset{g\in \mathcal {U} }{ \bigwedge }[{\mathcal {V}} _{\eth }(\jmath ,c)\bigwedge {\mathcal {V}} _{\gimel }(g)]; \\ \mu _{\underline{\eth }(\gimel )}(\jmath )= & {} \underset{g\in \mathcal {U} }{ \bigwedge }[\mu _{\eth }(\jmath ,c)\bigwedge \mu _{\gimel }(g)]; \\ {\mathcal {V}} _{\underline{\eth }(\gimel )}(\jmath )= & {} \underset{g\in \mathcal {U} }{ \bigvee }[{\mathcal {V}} _{\eth }(\jmath ,c)\bigvee {\mathcal {V}} _{\gimel }(g)]; \end{aligned}$$

such that $0\le ((\mu _{\underline{\eth }(\gimel )}(\jmath ))^{3}+({\mathcal {V}} _{ \underline{\eth }(\gimel )}(\jmath ))^{3})\le 1,$ and $0\le \left( \left( \mu _{\overline{\eth }(\gimel )}(\jmath )\right) ^{3}+\left( {\mathcal {V}} _{\overline{\eth }(\gimel )}(\jmath )\right) ^{3}\right) \le 1.$ As $\left( \underline{\eth }(\gimel ),\overline{ \eth }(\gimel )\right)$ are *FFSs*,  so $\underline{\eth }(\gimel ),$ $\overline{\eth }(\gimel ):FFS(\mathcal {U} )\rightarrow FFS(\mathcal {U} )$ are LA and UA operators. The pair $\eth (\gimel )=( \underline{\eth }(\gimel ),\overline{\eth }(\gimel ))=\{\left\langle \jmath ,(\mu _{\underline{\eth }(\gimel )}(\jmath ),{\mathcal {V}} _{\underline{\eth } (\gimel )}(\jmath ),(\mu _{\overline{\eth }(\gimel )}(\jmath ),{\mathcal {V}} _{ \overline{\eth }(\gimel )}(\jmath ))\right\rangle |\jmath \in \gimel \}$ is called FFRS. Just because of simplicity, $\eth (\gimel )=\{\left\langle \jmath ,\mu _{\underline{\eth }(\gimel )}(\jmath ),{\mathcal {V}} _{ \underline{\eth }(\gimel )}(\jmath ),(\mu _{\overline{\eth }(\gimel )}(\jmath ),{\mathcal {V}} _{\overline{\eth }(\gimel )}(\jmath ))\right\rangle |\jmath \in \mathcal {U} \}$ is described as $\eth (\gimel )=((\underline{\mu }, \underline{{\mathcal {V}} }),(\overline{\mu },\overline{{\mathcal {V}} }))$ and is called as FFR value (FFRV).

### Definition 2.11

^21^Suppose $\mathcal {U}$ is a universal set and for any subset $\eth \in FHFS(\mathcal {U} \times \mathcal {U} )$ is known as Fermatean hesitant fuzzy relation. The pair $\left( \mathcal {U} ,\eth \right)$ is called to be FHF approximation space. If for any $\gimel \subseteq FHFS(\mathcal {U} )$, then the LA and UA of $\gimel$ w.r.t. FHF approximation space $\left( \mathcal {U} ,\eth \right)$ are two FHFSs, which are symbolised by $\overline{\eth }(\gimel )$ and $\underline{\eth } (\gimel )$ and described as follows:

$$\begin{aligned} \overline{\eth }(\gimel )= & {} \left\{ \left\langle \jmath ,\zeta _{h_{ \overline{\eth }(\gimel )}}(\jmath ),\mathcal {J} _{h_{\overline{\eth }(\gimel )}}(\jmath )\right\rangle |\jmath \in \mathcal {U} \right\} ; \\ \underline{\eth }(\gimel )= & {} \left\{ \left\langle \jmath ,\zeta _{h_{ \underline{\eth }(\gimel )}}(\jmath ),\mathcal {J} _{h_{\underline{\eth } (\gimel )}}(\jmath )\right\rangle |\jmath \in \mathcal {U} \right\} ; \end{aligned}$$

where

$$\begin{aligned} \zeta _{h_{\overline{\eth }(\gimel )}}(\jmath )= & {} \underset{k\in \mathcal {U} }{ \bigvee }\left[ \zeta _{h_{\eth }}(\jmath ,k)\bigvee \zeta _{h_{\gimel }}(k) \right] ; \\ \mathcal {J} _{h_{\overline{\eth }(\gimel )}}(\jmath )= & {} \underset{k\in \mathcal {U} }{ \bigwedge }\left[ \mathcal {J} _{h_{\eth }}(\jmath ,k)\bigwedge \mathcal {J} _{h_{\gimel }}(k)\right] ; \\ \zeta _{h_{\underline{\eth }(\gimel )}}(\jmath )= & {} \underset{k\in \mathcal {U} }{ \bigwedge }\left[ \zeta _{h_{\eth }}(\jmath ,k)\bigwedge \zeta _{h_{\gimel }}(k)\right] ; \\ \mathcal {J} _{h_{\underline{\eth }(\gimel )}}(\jmath )= & {} \underset{k\in \mathcal {U} }{ \bigvee }\left[ \mathcal {J} _{h_{\eth }}(\jmath ,k)\bigvee \mathcal {J} _{h_{\gimel }}(k) \right] ; \end{aligned}$$

such that $0\le \left( \min (\zeta _{h_{\underline{\eth } (\gimel )}}(\jmath )\right) ^{3}+\left( \max (\mathcal {J} _{h_{\underline{\eth } (\gimel )}}(\jmath ))\right) ^{3}\le 1$ and $0\le \left( \max (\zeta _{h_{\overline{\eth }(\gimel )}}(\jmath ))\right) ^{3}+\left( \min (\mathcal {J} _{h_{\overline{\eth }(\gimel )}}(\jmath ))\right) ^{3}\le 1.$ As $\left( \underline{\eth } (\gimel ),\overline{\eth }(\gimel )\right)$ are *FHFSs*,  so $\underline{\eth }(\gimel ),\overline{\eth }(\gimel ):FHFS(\mathcal {U} )\rightarrow FFS(\mathcal {U} )$ are LA and UA operators. The pair

$$\begin{aligned} \eth (\gimel )=\left( \underline{\eth }(\gimel ),\overline{\eth } (\gimel )\right) =\left\{ \left\langle \jmath ,\left( \zeta _{h_{\underline{ \eth }(\gimel )}}(\jmath ),\mathcal {J} _{h_{\underline{\eth }(\gimel )}}(\jmath )\right) ,\left( \zeta _{h_{\overline{\eth }(\gimel )}}(\jmath ),\mathcal {J} _{h_{ \overline{\eth }(\gimel )}}(\jmath )\right) \right\rangle |\jmath \in \gimel \right\} \end{aligned}$$

will be called Fermatean hesitant fuzzy rough set. Just because of simplicity

$$\begin{aligned} \eth (\gimel )=\left\{ \left\langle \jmath ,\left( \zeta _{h_{\underline{\eth }(\gimel )}}(\jmath ),\mathcal {J} _{h_{\underline{\eth }(\gimel )}}(\jmath )\right) ,\left( \zeta _{h_{\overline{\eth }(\gimel )}}(\jmath ),\mathcal {J} _{h_{ \overline{\eth }(\gimel )}}(\jmath )\right) \right\rangle |\jmath \in \gimel \right\} \end{aligned}$$

is written as $\eth (\gimel )=\left( (\underline{\zeta },\underline{ \mathcal {J} }),(\overline{\zeta },\overline{\mathcal {J} })\right)$ and is called as FHFR value. For explanation of the above concept of FHFRS, we present the following example.

### Example 2.12

Suppose $\mathcal {U} =\left\{ \varphi _{1},\varphi _{2},\varphi _{3},\varphi _{4}\right\}$ be any fixed set and $\left( \mathcal {U} ,\eth \right)$ is FHF approximation space where $\eth \in FHFRS(\mathcal {U} \times \mathcal {U} )$ is the FHFR relation shown in Table 1. A decision expert now provides the ideal normal decision object $\gimel$ (in the form of FHFRS).

**Table 1:** FHFR  relation  in  $\mathcal {U}$.

$\gimel$	$c_{1}$	$c_{2}$	$c_{3}$	$c_{4}$
$\varphi _{1}$	$$\left( \begin{array}{l} \left\{ 0.1,0.3,0.4\right\} , \\ \left\{ 0.2,0.5,0.7\right\} \end{array} \right)$$	$$\left( \begin{array}{l} \left\{ 0.2,0.3\right\} , \\ \left\{ 0.7,0.9\right\} \end{array} \right)$$	$$\left( \begin{array}{l} \left\{ 0.2,0.5,0.7\right\} , \\ \left\{ 0.2,0.3\right\} \end{array} \right)$$	$$\left( \begin{array}{l} \left\{ 0.3,0.5\right\} , \\ \left\{ 0.8\right\} \end{array} \right)$$
$\varphi _{2}$	$$\left( \begin{array}{l} \left\{ 0.2,0.3,0.5\right\} , \\ \left\{ 0.2,0.7\right\} \end{array} \right)$$	$$\left( \begin{array}{l} \left\{ 0.2,0.3,,0.5\right\} , \\ \left\{ 0.3,0.4\right\} \end{array} \right)$$	$$\left( \begin{array}{l} \left\{ 0.1,0.4,0.6\right\} , \\ \left\{ 0.7,0.9\right\} \end{array} \right)$$	$$\left( \begin{array}{l} \left\{ 0.2,0.4\right\} , \\ \left\{ 0.7\right\} \end{array} \right)$$
$\varphi _{3}$	$$\left( \begin{array}{l} \left\{ 0.5,0.6\right\} , \\ \left\{ 0.7,0.9\right\} \end{array} \right)$$	$$\left( \begin{array}{l} \left\{ 0.5,0.8,0.9\right\} , \\ \left\{ 0.1,0.9\right\} \end{array} \right)$$	$$\left( \begin{array}{l} \left\{ 0.2,0.3\right\} , \\ \left\{ 0.5,0.9\right\} \end{array} \right)$$	$$\left( \begin{array}{l} \left\{ 0.7,0.9\right\} , \\ \left\{ 0.1,0.2,0.3\right\} \end{array} \right)$$
$\varphi _{4}$	$$\left( \begin{array}{l} \left\{ 0.2,0.5,0.9\right\} , \\ \left\{ 0.6,0.7,0.9\right\} \end{array} \right)$$	$$\left( \begin{array}{l} \left\{ 0.3,0.8,0.9\right\} , \\ \left\{ 0.4,0.8\right\} \end{array} \right)$$	$$\left( \begin{array}{l} \left\{ 0.2,0.5\right\} , \\ \left\{ 0.6,0.9\right\} \end{array} \right)$$	$$\left( \begin{array}{l} \left\{ 0.5,0.7\right\} , \\ \left\{ 0.1,0.8\right\} \end{array} \right)$$

and

$$\begin{aligned} \gimel =\left\{ \begin{array}{l} \left\langle \varphi _{1},\left\{ 0.2,0.3,0.4\right\} ,\left\{ 0.5,0.7\right\} \right\rangle ,\left\langle \varphi _{2},\left\{ 0.2,0.3,0.7\right\} ,\left\{ 0.1,0.7,0.8\right\} \right\rangle , \\ \left\langle \varphi _{3},\left\{ 0.5,0.7,0.8\right\} ,\left\{ 0.1,0.5,0.7\right\} \right\rangle ,\left\langle \varphi _{4},\left\{ 0.6,0.8,0.9\right\} ,\left\{ 0.2,0.6,0.7\right\} \right\rangle \end{array} \right\} . \end{aligned}$$

Then it follows that

$$\begin{aligned} \zeta _{h_{\overline{\eth }(\gimel )}}(\varphi _{1})= & {} \underset{k\in \mathcal {U} }{\bigvee }\left[ \zeta _{h_{\eth }}(\varphi ,c)\bigvee \zeta _{h_{\gimel }}(k)\right] \\= & {} \left\{ \begin{array}{l} \left\{ 0.1\vee 0.2,0.3\vee 0.3,0.4\vee 0.4\right\} \vee \\ \left\{ 0.2\vee 0.2,0.3\vee 0.3,0\vee 0.7\right\} \vee \\ \left\{ 0.2\vee 0.5,0.5\vee 0.7,0.7\vee 0.8\right\} \vee \\ \left\{ 0.3\vee 0.6,0.5\vee 0.8,0\vee 0.9\right\} \end{array} \right\} \\= & {} \left\{ \begin{array}{l} \left\{ 0.2,0.3,0.4\right\} \vee \left\{ 0.2,0.3,0\right\} \vee \\ \left\{ 0.5,0.7,0.8\right\} \vee \left\{ 0.6,0.8,0.9\right\} \end{array} \right\} \\= & {} \left\{ 0.6,0.8,0.9\right\} \end{aligned}$$

Similarly, we can find the remaining values as:

$$\begin{aligned} \begin{array}{cc} \zeta _{h_{\overline{\eth }(\gimel )}}(\varphi _{2})=\left\{ 0.6,0.8,0.9\right\} , &{} \zeta _{h_{\overline{\eth }(\gimel )}}(\varphi _{3})=\left\{ 0.7,0.9\right\} , \\ \zeta _{h_{\overline{\eth }(\gimel )}}(\varphi _{4})=\left\{ 0.6,0.8,0.9\right\} . &{} \end{array} \end{aligned}$$

Now,

$$\begin{aligned} \mathcal {J} _{h_{\overline{\eth }(\gimel )}}(\varphi _{1})= & {} \underset{k\in \mathcal {U} }{\bigwedge }\left[ \mathcal {J} _{h_{\eth }}(\varphi ,c)\bigwedge \mathcal {J} _{h_{\gimel }}(k)\right] \\= & {} \left\{ \begin{array}{l} \left\{ 0.2\wedge 0.5,0.5\wedge 0.7,0\wedge 0.7\right\} \wedge \\ \left\{ 0.7\wedge 0.1,0.9\wedge 0.7,0\wedge 0.8\right\} \wedge \\ \left\{ 0.2\wedge 0.1,0.3\wedge 0.5,0\wedge 0.7\right\} \wedge \\ \left\{ 0.8\wedge 0.2,0\wedge 0.6,0\wedge 0.7\right\} \end{array} \right\} \\= & {} \left\{ \left\{ 0.2,0.5\right\} \wedge \left\{ 0.1,0.7\right\} \wedge \left\{ 0.2,0.3\right\} \wedge \left\{ 0.2\right\} \right\} , \\= & {} \left\{ 0.2\right\} . \end{aligned}$$

By the routine calculations, we get

$$\begin{aligned} \begin{array}{ccc} \mathcal {J} _{h_{\overline{\eth }(\gimel )}}(\varphi _{2})=\left\{ 0.1\right\} ,&\mathcal {J} _{h_{\overline{\eth }(\gimel )}}(\varphi _{3})=\left\{ 0.1,0.2\right\} ,&\mathcal {J} _{h_{\overline{\eth }(\gimel )}}(\varphi _{4})=\left\{ 0.1,0.5\right\} . \end{array} \end{aligned}$$

Further,

$$\begin{aligned} \zeta _{h_{\underline{\eth }(\gimel )}}(\varphi _{1})= & {} \underset{k\in \mathcal {U} }{\bigwedge }\left[ \zeta _{h_{\eth }}(\varphi ,c)\bigwedge \zeta _{h_{\gimel }}(k)\right] \\= & {} \left\{ \begin{array}{l} \left\{ 0.1\wedge 0.2,0.3\wedge 0.3,0.4\wedge 0.4\right\} \wedge \\ \left\{ 0.2\wedge 0.2,0.3\wedge 0.3,0\wedge 0.7\right\} \wedge \\ \left\{ 0.2\wedge 0.5,0.5\wedge 0.7,0.7\wedge 0.8\right\} \wedge \\ \left\{ 0.3\wedge 0.6,0.5\wedge 0.8,0\wedge 0.9\right\} \end{array} \right\} \\= & {} \left\{ \begin{array}{l} \left\{ 0.1,0.3,0.4\right\} \wedge \left\{ 0.2,0.3\right\} \wedge \\ \left\{ 0.2,0.5,0.7\right\} \wedge \left\{ 0.3,0.5\right\} \end{array} \right\} \\= & {} \left\{ 0.1,0.3\right\} . \end{aligned}$$

By the routine calculations, we get

$$\begin{aligned} \begin{array}{ccc} \zeta _{h_{\underline{\eth }(\gimel )}}(\varphi _{2})=\left\{ 0.1,0.3\right\} ,&\zeta _{h_{\underline{\eth }(\gimel )}}(\varphi _{3})=\left\{ 0.2,0.3\right\} ,&\zeta _{h_{\underline{\eth }(\gimel )}}(\varphi _{4})=\left\{ 0.2,0.3\right\} . \end{array} \end{aligned}$$

Now,

$$\begin{aligned} \mathcal {J} _{h_{\underline{\eth }(\gimel )}}(\varphi _{1})= & {} \underset{k\in \mathcal {U} }{\bigvee }\left[ \mathcal {J} _{h_{\eth }}(\varphi ,c)\bigvee \mathcal {J} _{h_{\gimel }}(k)\right] \\= & {} \left\{ \begin{array}{l} \left\{ 0.2\vee 0.5,0.5\vee 0.7,0.7\vee 0\right\} \vee \\ \left\{ 0.7\vee 0.2,0.9\vee 0.3,0\vee 0.7\right\} \vee \\ \left\{ 0.2\vee 0.1,0.3\vee 0.5,0\vee 0.7\right\} \vee \\ \left\{ 0.8\vee 0.2,0\vee 0.6,0\vee 0.7\right\} \end{array} \right\} \\= & {} \left\{ \begin{array}{l} \left\{ 0.5,0.7,0.7\right\} \vee \left\{ 0.7,0.9,0.7\right\} \vee \\ \left\{ 0.2,0.5,0.7\right\} \vee \left\{ 0.8,0.6,0.7\right\} \end{array} \right\} \\= & {} \left\{ 0.8,0.9,0.7\right\} . \end{aligned}$$

Keeping on the same route, the remaining values may be determined as follows:

$$\begin{aligned} \begin{array}{ccc} \mathcal {J} _{h_{\underline{\eth }(\gimel )}}(\varphi _{2})=\left\{ 0.7,0.9\right\} ,&\mathcal {J} _{h_{\underline{\eth }(\gimel )}}(\varphi _{3})=\left\{ 0.7,0.9,0.8\right\} ,&\mathcal {J} _{h_{\underline{\eth }(\gimel )}}(\varphi _{4})=\left\{ 0.6,0.9,0.9\right\} . \end{array} \end{aligned}$$

The LA and UA operators in the form of FHFR approximation are follows:

$$\begin{aligned}{} & {} \underline{\eth }(\gimel )=\left\{ \begin{array}{l} \left\langle \varphi _{1},\left\{ 0.1,0.3\right\} ,\left\{ 0.8,0.9,0.7\right\} \right\rangle ,\left\langle \varphi _{2},\left\{ 0.1,0.3\right\} ,\left\{ 0.7,0.9\right\} \right\rangle , \\ \left\langle \varphi _{3},\left\{ 0.2,0.3\right\} ,\left\{ 0.7,0.9,0.8\right\} \right\rangle ,\left\langle \varphi _{3},\left\{ 0.2,0.3\right\} ,\left\{ 0.6,0.9,0.9\right\} \right\rangle \end{array} \right\} ,\\{} & {} \overline{\eth }(\gimel )=\left\{ \begin{array}{l} \left\langle \varphi _{1},\left\{ 0.6,0.8,0.9\right\} ,\left\{ 0.2\right\} \right\rangle ,\left\langle \varphi _{2},\left\{ 0.6,0.8,0.9\right\} ,\left\{ 0.1\right\} \right\rangle , \\ \left\langle \varphi _{3},\left\{ 0.7,0.9,0.9\right\} ,\left\{ 0.1,0.2\right\} \right\rangle ,\left\langle \varphi _{4},\left\{ 0.6,0.8,0.9\right\} ,\left\{ 0.1,0.5\right\} \right\rangle \end{array} \right\} . \end{aligned}$$

Hence

$$\begin{aligned} \eth (\gimel )= & {} (\underline{\eth }(\gimel ),\overline{\eth } (\gimel )) \\= & {} \left\{ \begin{array}{l} \left\langle \varphi _{1},\left( \left\{ 0.1,0.3\right\} ,\left\{ 0.8,0.9,0.7\right\} \right) ,\left( \left\{ 0.6,0.8,0.9\right\} ,\left\{ 0.2\right\} \right) \right\rangle , \\ \left\langle \varphi _{2},\left( \left\{ 0.1,0.3\right\} ,\left\{ 0.7,0.9\right\} \right) ,\left( \left\{ 0.6,0.8,0.9\right\} ,\left\{ 0.1\right\} \right) \right\rangle , \\ \left\langle \varphi _{3},\left( \left\{ 0.2,0.3\right\} ,\left\{ 0.7,0.9,0.8\right\} \right) 
,\left( \left\{ 0.7,0.9,0.9\right\} ,\left\{ 0.1,0.2\right\} \right) \right\rangle , \\ \left\langle \varphi _{3},\left( \left\{ 0.2,0.3\right\} ,\left\{ 0.6,0.9,0.9\right\} \right) ,\left( \left\{ 0.6,0.8,0.9\right\} ,\left\{ 0.1,0.5\right\} \right) \right\rangle \end{array} \right\} . \end{aligned}$$

### Definition 2.13

Let $\eth (\gimel _{1})=(\underline{\eth }(\gimel _{1}),\overline{ \eth }(\gimel _{1}))$ and $\eth (\gimel _{2})=(\underline{\eth } (\gimel _{2}),\overline{\eth }(\gimel _{2}))$ be two FHFRSs. Then

1. $$\eth (\gimel _{1})\cup$$ $$\eth (\gimel _{2})=\{(\underline{\eth } (\gimel _{1})\cup \underline{\eth }(\gimel _{2})),(\overline{\eth } (\gimel _{1})\cup \overline{\eth }(\gimel _{2}))\}$$
2. $$\eth (\gimel _{1})\cap$$ $$\eth (\gimel _{2})=\{(\underline{\eth } (\gimel _{1})\cap \underline{\eth }(\gimel _{2})),(\overline{\eth } (\gimel _{1})\cap \overline{\eth }(\gimel _{2}))\}.$$

### Definition 2.14

Let $\eth (\gimel _{1})=(\underline{\eth }(\gimel _{1}),\overline{ \eth }(\gimel _{1}))$ and $\eth (\gimel _{2})=(\underline{\eth } (\gimel _{2}),\overline{\eth }(\gimel _{2}))$ be two FHFRSs. Then

1. $$\eth (\gimel _{1})\oplus$$ $$\eth (\gimel _{2})=\{(\underline{ \eth }(\gimel _{1})\oplus \underline{\eth }(\gimel _{2})),(\overline{ \eth }(\gimel _{1})\oplus \overline{\eth }(\gimel _{2}))\}$$
2. $$\eth (\gimel _{1})\otimes$$ $$\eth (\gimel _{2})=\{(\underline{ \eth }(\gimel _{1})\otimes \underline{\eth }(\gimel _{2})),( \overline{\eth }(\gimel _{1})\otimes \overline{\eth }(\gimel _{2}))\}$$
3. $$\eth (\gimel _{1})\subseteq$$ $$\eth (\gimel _{2})=\{(\underline{ \eth }(\gimel _{1})\subseteq \underline{\eth }(\gimel _{2}))$$ and $$( \overline{\eth }(\gimel _{1})\subseteq \overline{\eth }(\gimel _{2}))\}$$
4. $$\varsigma \eth (\gimel _{1})=(\varsigma \underline{\eth }(\gimel _{1}),$$ $$\varsigma \overline{\eth }(\gimel _{1}))$$ for $$\varsigma \ge 1$$
5. $$(\eth (\gimel _{1}))^{\varsigma }=((\underline{\eth }(\gimel _{1}))^{\varsigma },$$ $$(\overline{\eth }(\gimel _{1}))^{\varsigma })$$ for $$\varsigma \ge 1$$
6. $\eth (\gimel _{1})^{c}=(\underline{\eth }(\gimel _{1})^{c},$ $\overline{\eth }(\gimel _{1})^{c})$ where $\underline{\eth }(\gimel _{1})^{c}$ and $\overline{\eth }(\gimel _{1})^{c}$ illustrate the complement of FFR approximation operators $\underline{\eth }(\gimel _{1})$ and $\overline{\eth }(\gimel _{1}),$  that is $\underline{\eth } (\gimel _{1})^{c}=\left( \mathcal {J} _{h_{\underline{\eth }(\gimel )}},\zeta _{h_{\underline{\eth }(\gimel )}}\right) .$
7. $$\eth (\gimel _{1})=$$ $$\eth (\gimel _{2})$$ iff $$\underline{\eth } (\gimel _{1})=\underline{\eth }(\gimel _{2})$$ and $$\overline{\eth } (\gimel _{1})=\overline{\eth }(\gimel _{2}).$$

The score function will be utilized to compare and rank two or more FHFR values. Greater FHFR score values indicate superiority, whilst lower FHFR score values indicate inferiority. We will employ the accuracy function when the score values are identical.

### Definition 2.15

The function for scoring FHFR value $\eth (\gimel )=( \underline{\eth }(\gimel ),\overline{\eth }(\gimel ))=((\underline{ \zeta },\underline{\mathcal {J} }),(\overline{\zeta },\overline{\mathcal {J} }))$ is given as:

$$\begin{aligned} \Game (\eth (\gimel ))=\frac{1}{4}\left( \begin{array}{l} 2+\frac{1}{M_{\mathcal {R} }}\sum \nolimits _{\underline{\mu _{\imath }}\in \zeta _{h_{ \underline{\eth }(\gimel )}}}\left\{ \underline{\mu _{\imath }}\right\} +\frac{ 1}{N_{\mathcal {R} }}\sum \nolimits _{\overline{\mu _{\imath }}\in \zeta _{h_{ \overline{\eth }(\gimel )}}}\left\{ \overline{\mu _{\imath }}\right\} - \\ \frac{1}{M_{\mathcal {R} }}\sum \nolimits _{\underline{{\mathcal {V}} _{\imath }}\in \mathcal {J} _{h_{ \underline{\eth }(\gimel )}}}(\underline{{\mathcal {V}} _{\imath }})-\frac{1}{ M_{\mathcal {R} }}\sum \nolimits _{\overline{{\mathcal {V}} _{\imath }}\in \mathcal {J} _{h_{\overline{ \eth }(\gimel )}}}(\overline{{\mathcal {V}} _{\imath }}) \end{array} \right) . \end{aligned}$$

The accuracy function for FHFR value $\eth (\gimel )=(\underline{\eth } (\gimel ),\overline{\eth }(\gimel ))=((\underline{\zeta },\underline{ \mathcal {J} }),(\overline{\zeta },\overline{\mathcal {J} }))$ is given as:

$$\begin{aligned} \textbf{AC}\eth (\gimel )=\frac{1}{4}\left( \begin{array}{l} \frac{1}{M_{\mathcal {R} }}\sum \nolimits _{\mu _{\imath }\in \zeta _{h_{\overline{\eth }(\gimel )}}}(\overline{\mu _{\imath }})+\frac{1}{M_{\mathcal {R} }} \sum \nolimits _{\mu _{\imath }\in \zeta _{h_{\overline{\eth }(\gimel )}}}( \overline{\mu _{\imath }})+ \\ \frac{1}{M_{\mathcal {R} }}\sum \nolimits _{\underline{{\mathcal {V}} _{\imath }}\in \mathcal {J} _{h_{ \underline{\eth }(\gimel )}}}(\underline{{\mathcal {V}} _{\imath }})+\frac{1}{ M_{\mathcal {R} }}\sum \nolimits _{\overline{{\mathcal {V}} _{\imath }}\in \mathcal {J} _{h_{\overline{ \eth }(\gimel )}}}(\overline{{\mathcal {V}} _{\imath }}) \end{array} \right) , \end{aligned}$$

where $$M_{\mathcal {R} }$$ and $$N_{\mathcal {R} }$$ show the number of elements in $$\zeta _{h_{g}}$$ and $$\mathcal {J} 
_{h_{g}}$$, respectively.

### Definition 2.16

Suppose $\eth (\gimel _{1})=(\underline{\eth }(\gimel _{1}), \overline{\eth }(\gimel _{1}))$ and $\eth (\gimel _{2})=(\underline{ \eth }(\gimel _{2}),\overline{\eth }(\gimel _{2}))$ are two FHFRVs. Then

1. If $$\Game (\eth (\gimel _{1}))>\Game (\eth (\gimel _{2})),$$ then $$\eth (\gimel _{1})>\eth (\gimel _{2}),$$
2. If $$\Game (\eth (\gimel _{1}))\prec \Game (\eth (\gimel _{2})),$$ then $$\eth (\gimel _{1})\prec \eth (\gimel _{2}),$$
3. If $$\Game (\eth (\gimel _{1}))=\Game (\eth (\gimel _{2})),$$ then
  - If $$\textbf{AC}\eth (\gimel _{1})>\textbf{AC}\eth (\gimel _{2})$$ then $$\eth (\gimel _{1})>\eth (\gimel _{2}),$$
  - If $$\textbf{AC}\eth (\gimel _{1})\prec \textbf{AC}\eth (\gimel _{2})$$ then $$\eth (\gimel _{1})\prec \eth (\gimel _{2}),$$
  - If $$\textbf{AC}\eth (\gimel _{1})=\textbf{AC}\eth (\gimel _{2})$$ then $$\eth (\gimel _{1})=\eth (\gimel _{2}).$$

## The Fermatean hesitant fuzzy rough aggregation operators

In this section, we establish the concept of FHFR aggregation operators by combining the idea of rough sets and FHF aggregation operators. Further we obtain aggregation notions for FHFRWA, FHFROWA, and FHFRHWA. Several fundamental characteristics of these notions are highlighted.

### The Fermatean hesitant fuzzy rough weighted averaging operator

#### Definition 3.1

Consider the collection $\eth (\gimel _{\imath })=(\underline{\eth } (\gimel _{\imath }),\overline{\eth }(\gimel _{\imath }))$ $(\imath =1,2,3,...,\ell )$ of FHFRVs with weight vector $\mathcal {W}=\left( \propto _{1},\propto _{2},...,\propto _{n}\right) ^{T}$ such that $\bigoplus _{i=1}^{\ell }\propto _{\imath }=1$ and $\propto _{\imath }\in [0, 1].$ The FHFRWA operator is identified as:

$$\begin{aligned} FHFRWA\left( \eth (\gimel _{1}),\eth (\gimel _{2}),...,\eth (\gimel _{\ell })\right) =\left( \bigoplus _{\imath =1}^{\ell }\propto _{\imath }\underline{\eth } (\gimel _{\imath }),\bigoplus _{\imath =1}^{\ell }\propto _{\imath }\overline{\eth }(\gimel _{\imath })\right) . \end{aligned}$$

#### Theorem 1

Let $\eth (\gimel _{\imath })=(\underline{\eth }(\gimel _{\imath }), \overline{\eth }(\gimel _{\imath }))$ $(\imath =1,2,3,...,\ell )$ be the collection of FHFRVs with weight vector $\mathcal {W}=\left( \propto _{1},\propto _{2},...,\propto _{\ell }\right) ^{T}.$ Then the FHFRWA operator is defined as:

$$\begin{aligned}{} & {} FHFRWA\left( \eth (\gimel _{1}),\eth (\gimel _{2}),...,\eth (\gimel _{\ell })\right) \\= & {} \left( \bigoplus _{i=1}^{\ell }\propto _{\imath }\underline{\eth }(\gimel _{\imath }),\bigoplus _{i=1}^{\ell }\propto _{\imath }\overline{\eth }(\gimel _{\imath })\right) \\= & {} \left[ \begin{array}{l} \bigcup \limits _{\underline{\mu _{\imath }}\in \zeta _{h_{\underline{\eth } (\gimel )}}}\root 3 \of {\left( 1-\overset{\ell }{\underset{i=1}{\boxtimes }} \left( 1-\left( \underline{\mu _{\imath }}\right) ^{3}\right) ^{\propto _{\imath }}\right) }, \text { }\bigcup \limits _{\underline{{\mathcal {V}} _{\imath }}\in \mathcal {J} _{h_{\underline{\eth } (\gimel )}}}\overset{\ell }{\underset{i=1}{\boxtimes }}\left( \underline{{\mathcal {V}} _{\imath }}\right) ^{^{\propto _{\imath }}} \\ \bigcup \limits _{\overline{\mu _{\imath }}\in \zeta _{h_{\overline{\eth }(\gimel )}}}\root 3 \of {\left( 1-\overset{\ell }{\underset{\imath =1}{\boxtimes }}\left( 1-\left( \overline{\mu _{\imath }}\right) ^{3}\right) ^{\propto _{\imath }}\right) },\text { } \bigcup \limits _{\overline{{\mathcal {V}} _{\imath }}\in \mathcal {J} _{h_{\overline{\eth }(\gimel )}}}\overset{\ell }{\underset{\imath =1}{\boxtimes }}\left( \overline{{\mathcal {V}} _{\imath }}\right) ^{^{\propto _{\imath }}} \end{array} \right] \end{aligned}$$

#### Proof

We employ the mathematical induction to get the required proof. In terms of the operational law, it follows that

$$\begin{aligned} \eth (\gimel _{1})\oplus \eth (\gimel _{2})=\left[ \underline{\eth } (\gimel _{1})\oplus \underline{\eth }(\gimel _{2}),\overline{\eth } (\gimel _{1})\oplus \overline{\eth }(\gimel _{2})\right] \end{aligned}$$

and

$$\begin{aligned} \varsigma \eth (\gimel _{1})=\left( \varsigma \underline{\eth }(\gimel _{1}),\varsigma \overline{\eth }(\gimel _{1})\right) \end{aligned}$$

If $$\ell =2$$, then

$$\begin{aligned}{} & {} FHFRWA\left( \eth (\gimel _{1}),\eth (\gimel _{2})\right) \\= & {} \left( \bigoplus _{\imath =1}^{2}\propto _{\imath }\underline{\eth }(\gimel _{\imath }),\bigoplus _{\imath =1}^{2}\propto _{\imath }\overline{\eth }(\gimel _{\imath })\right) \\= & {} \left( \begin{array}{l} \left( \bigcup \limits _{\underline{\mu _{\imath }}\in \zeta _{h_{\underline{\eth } (\gimel )}}}\root 3 \of {\left( 1-\overset{2}{\underset{\imath =1}{\boxtimes }} \left( 1-\left( \underline{\mu _{\imath }}\right) ^{3}\right) ^{\propto _{\imath }}\right) }, \text { }\bigcup \limits _{\underline{{\mathcal {V}} _{\imath }}\in \mathcal {J} _{h_{\underline{\eth } (\gimel )}}}\overset{2}{\underset{\imath =1}{\boxtimes }}\left( \underline{{\mathcal {V}} _{\imath }}\right) ^{^{\propto _{\imath }}}\right) \\ \left( \bigcup \limits _{\overline{\mu _{\imath }}\in \zeta _{h_{\overline{\eth } (\gimel )}}}\root 3 \of {\left( 1-\overset{2}{\underset{\imath =1}{\boxtimes }} \left( 1-\left( \overline{\mu _{\imath }}\right) ^{3}\right) ^{\propto _{\imath }}\right) }, \text { }\bigcup \limits _{\overline{{\mathcal {V}} _{\imath }}\in \mathcal {J} _{h_{\overline{\eth } (\gimel )}}}\overset{2}{\underset{\imath =1}{\boxtimes }}\left( \overline{{\mathcal {V}} _{\imath }}\right) ^{^{\propto _{\imath }}}\right) \end{array} \right) . \end{aligned}$$

For $\ell =2,$ the result is accurate. Assume it is true for $\ell =k,$ that is,

$$\begin{aligned}{} & {} FHFRWA\left( \eth (\gimel _{1}),\eth (\gimel _{2}),...,\eth (\gimel _{k})\right) \\= & {} \left( \bigoplus _{\imath =1}^{k}\propto _{\imath }\underline{\eth }(\gimel _{\imath }),\bigoplus _{\imath =1}^{k}\propto _{\imath }\overline{\eth }(\gimel _{\imath })\right) \\= & {} \left( \begin{array}{l} \left( \bigcup \limits _{\underline{\mu _{\imath }}\in \zeta _{h_{\underline{\eth } (\gimel )}}}\root 3 \of {\left( 1-\overset{k}{\underset{\imath =1}{\boxtimes }} \left( 1-\left( \underline{\mu _{\imath }}\right) ^{3}\right) ^{\propto _{\imath }}\right) }, \text { }\bigcup \limits _{\underline{{\mathcal {V}} _{\imath }}\in \mathcal {J} _{h_{\underline{\eth } (\gimel )}}}\overset{k}{\underset{\imath =1}{\boxtimes }}\left( \underline{{\mathcal {V}} _{\imath }}\right) ^{^{\propto _{\imath }}}\right) \\ \left( \bigcup \limits _{\overline{\mu _{\imath }}\in \zeta _{h_{\overline{\eth } (\gimel )}}}\root 3 \of {\left( 1-\overset{k}{\underset{\imath =1}{\boxtimes }} \left( 1-\left( \overline{\mu _{\imath }}\right) ^{3}\right) ^{\propto _{\imath }}\right) }, \text { }\bigcup \limits 
_{\overline{{\mathcal {V}} _{\imath }}\in \mathcal {J} _{h_{\overline{\eth } (\gimel )}}}\overset{k}{\underset{\imath =1}{\boxtimes }}\left( \overline{{\mathcal {V}} _{\imath }}\right) ^{^{\propto _{\imath }}}\right) \end{array} \right) . \end{aligned}$$

Now we need to prove it for $\ell =k+1.$

$$\begin{aligned} Consider{} & {} FHFRWA\left( \eth (\gimel _{1}),\eth (\gimel _{2}),...,\eth (\gimel _{k+1})\right) \\= & {} \left( \begin{array}{l} \left( \bigoplus _{\imath =1}^{k}\propto _{\imath }\underline{\eth }\left( \gimel _{\imath }\right) \oplus \propto _{k+1}\underline{\eth }(\gimel _{k+1})\right) , \\ \left( \bigoplus _{\imath =1}^{k}\propto _{\imath }\overline{\eth }\left( \gimel _{\imath }\right) \oplus \propto _{k+1}\overline{\eth }(\gimel _{k+1})\right) \end{array} \right) , \\= & {} \left( \begin{array}{l} \left( \bigcup \limits _{\underline{\mu _{\imath }}\in \zeta _{h_{\underline{\eth } (\gimel )}}}\root 3 \of {\left( 1-\overset{k+1}{\underset{\imath =1}{\boxtimes }} \left( 1-\left( \underline{\mu _{\imath }}\right) ^{3}\right) ^{\propto _{\imath }}\right) }, \text { }\bigcup \limits _{\underline{{\mathcal {V}} _{\imath }}\in \mathcal {J} _{h_{\underline{\eth } (\gimel )}}}\overset{k+1}{\underset{\imath =1}{\boxtimes }}\left( \underline{{\mathcal {V}} _{\imath }}\right) ^{^{\propto _{\imath }}}\right) \\ \left( \bigcup \limits _{\overline{\mu _{\imath }}\in \zeta _{h_{\overline{\eth } (\gimel )}}}\root 3 \of {\left( 1-\overset{k+1}{\underset{\imath =1}{\boxtimes }} \left( 1-\left( \overline{\mu _{\imath }}\right) ^{3}\right) ^{\propto _{\imath }}\right) }, \text { }\bigcup \limits _{\overline{{\mathcal {V}} _{\imath }}\in \mathcal {J} _{h_{\overline{\eth } (\gimel )}}}\overset{k+1}{\underset{\imath =1}{\boxtimes }}\left( \overline{{\mathcal {V}} _{\imath }}\right) ^{^{\propto _{\imath }}}\right) \end{array} \right) . \end{aligned}$$

Therefore, the obtained outcome holds for $\ell =k+1$. As a result, the finding is applicable for all $\ell \ge 1.$ Based on the preceding analysis $\underline{\eth }(\gimel )$ and $\overline{\eth } (\gimel )$ are FHFRVs. So, $\bigoplus _{\imath =1}^{k}\propto _{\imath }\underline{\eth } \left( \gimel _{\imath }\right)$ and $\bigoplus _{\imath =1}^{k}\propto _{\imath }\overline{\eth } \left( \gimel _{\imath }\right)$ are also FHFRVs. Therefore, FHFRWA $\left( \eth (\gimel _{1}),\eth (\gimel _{2}),...,\eth (\gimel _{\ell })\right)$ is a FHFRV under FHF approximation space $\left( \mathcal {U} ,\eth \right) .$

#### Example 3.2

Consider the set

$$\begin{aligned} \gimel \subseteq \mathcal {U} =\left\{ \begin{array}{l} \left( \begin{array}{l} \varphi _{1},\left\langle \left\{ 0.20,0.30,0.40\right\} ,\left\{ 0.20,0.40,0.70\right\} \right\rangle , \\ \left\langle \left\{ 0.50,0.70,0.90\right\} ,\left\{ 0.20,0.50,0.70\right\} \right\rangle \end{array} \right) , \\ \left( \begin{array}{l} \varphi _{2},\left\langle \left\{ 0.40,0.70,0.90\right\} ,\left\{ 0.50,0.80,0.90\right\} \right\rangle , \\ \left\langle \left\{ 0.20,0.30,0.40\right\} ,\left\{ 0.10,0.20,0.30\right\} \right\rangle \end{array} \right) , \\ \left( \begin{array}{l} \varphi _{3},\left\langle \left\{ 0.20,0.30,0.40\right\} ,\left\{ 0.10,0.50,0.70\right\} \right\rangle , \\ \left\langle \left\{ 0.40,0.60,0.80\right\} ,\left\{ 0.20,0.40,0.60\right\} \right\rangle \end{array} \right) , \end{array} \right\} , \end{aligned}$$

with weight vector $\mathcal {W}=\left\{ 0.25,0.42,0.33\right\} ^{T}.$ It follows that

$$\begin{aligned}{} & {} FHFRWA\left( \eth (\gimel _{1}),\eth (\gimel _{2}),\eth (\gimel _{3})\right) =\left( \bigoplus _{\imath =1}^{3}\propto _{\imath }\underline{\eth } (\gimel _{\imath }),\bigoplus _{\imath =1}^{3}\propto _{\imath }\overline{\eth }(\gimel _{\imath })\right) \\{} & {} \quad =\left( \begin{array}{l} \left( \begin{array}{l} \left( \begin{array}{l} \left( \left( 1-(1-0.2^{3})^{0.25}(1-0.4^{3})^{0.42}(1-0.2^{3})^{0.33}\right) \right) ^{ \frac{1}{3}}, \\ \left( \left( 1-(1-0.3^{3})^{0.25}(1-0.7^{3})^{0.42}(1-0.3^{3})^{0.33}\right) \right) ^{{\frac{1}{3}}}, \\ \left( \left( 1-(1-0.4^{3})^{0.25}(1-0.7^{3})^{0.42}(1-0.4^{3})^{0.33}\right) \right) ^{{ \frac{1}{3}}} \end{array} \right) , \\ \left( \begin{array}{l} \left( 0.2^{0.25}\times 0.5^{0.42}\times 0.1^{0.33}\right) ,\left( 0.4^{0.25}\times 0.8^{0.42}\times 0.5^{0.33}\right) , \\ \left( 0.7^{0.25}\times 0.9^{0.42}\times 0.7^{0.33}\right) \end{array} \right) \end{array} \right) \\ \left( \begin{array}{l} \left( \begin{array}{l} \left( \left( 1-(1-0.5^{3})^{0.25}(1-0.2^{3})^{0.42}(1-0.4^{3})^{0.33}\right) \right) ^{{\frac{1}{3}}}, \\ \left( \left( 1-(1-0.7^{3})^{0.25}(1-0.3^{3})^{0.42}(1-0.6^{3})^{0.33}\right) \right) ^{{\frac{1}{3}}}, \\ \left( \left( 1-(1-0.9^{3})^{0.25}(1-0.4^{3})^{0.42}(1-0.8^{3})^{0.33}\right) \right) ^{{ \frac{1}{3}}} \end{array} \right) , \\ \left( \begin{array}{l} \left( 0.2^{0.25}\times 0.1^{0.42}\times 0.2^{0.33}\right) ,\left( 0.5^{0.25}\times 0.2^{0.42}\times 0.4^{0.33}\right) , \\ \left( 0.7^{0.25}\times 0.3^{0.42}\times 0.6^{0.33}\right) \end{array} \right) \end{array} \right) \end{array} \right) \\{} & {} \quad =\left( \begin{array}{l} \left( \left\{ 0.3172,0.5592,0.5781\right\} ,\left\{ 0.2337,0.5760,0.7779\right\} \right) , \\ \left( \left\{ 0.3846,0.5632,0.7641\right\} ,\left\{ 0.1494,0.3161,0.4660\right\} \right) \end{array} \right) . \end{aligned}$$

#### Theorem 2

Consider the collection $\eth (\gimel _{\imath })=(\underline{ \eth }(\gimel _{\imath }),\overline{\eth }(\gimel _{\imath }))$ $(\imath =1,2,3,...,\ell )$ of FHFRVs with weight vectors $\mathcal {W}=\left( \propto _{1},\propto _{2},...,\propto _{\ell }\right) ^{T}$ such that $\bigoplus _{\imath =1}^{\ell }\propto _{\imath }=1$ and $\propto _{\imath }\in [0, 1].$ Then the FHFRWA operator must fulfill the following properties:

(1) **Idempotency: **If $\eth (\gimel _{\imath })=\mathcal {G} (\gimel )$ for $\left( \imath =1,2,3,...,\ell \right) ,$ where $\mathcal {G} (\gimel )=\left( \underline{\mathcal {G} }(\gimel ),\overline{ \mathcal {G} }(\gimel )\right) =\left( (\underline{\partial },\underline{d}),( \overline{\partial },\overline{d})\right) .$ Then

$$\begin{aligned} FHFRWA\left( \eth (\gimel _{1}),\eth (\gimel _{2}),...,\eth (\gimel _{\ell })\right) =\mathcal {G} (\gimel ). \end{aligned}$$

(2) **Boundedness: **Let $\left( \eth (\gimel )\right) ^{-}=\left( \underset{\imath }{\min }\underline{\eth }\left( \gimel _{\imath }\right) ,\underset{ \imath }{\max }\overline{\eth }(\gimel _{\imath })\right)$ and $\left( \eth (\gimel )\right) ^{+}=$ $\left( \underset{\imath }{\max }\underline{\eth } \left( \gimel _{\imath }\right) ,\underset{\imath }{\min }\overline{\eth } (\gimel _{\imath })\right) .$ Then

$$\begin{aligned} \left( \eth (\gimel )\right) ^{-}\le FHFRWA\left( \eth (\gimel _{1}),\eth (\gimel _{2}),...,\eth (\gimel _{\ell })\right) \le \left( \eth (\gimel )\right) ^{+}. \end{aligned}$$

(3) **Monotonicity: **Suppose $\mathcal {G} (\gimel )=\left( \underline{\mathcal {G} }(\gimel _{\imath }),\overline{ \mathcal {G} }(\gimel _{\imath })\right) (\imath =1,2,...,n)$ be another collection of FHFRVs such that $\underline{\mathcal {G} }(\gimel _{\imath })\le \underline{\eth }\left( \gimel _{\imath }\right)$ and $\overline{\mathcal {G} } (\gimel _{\imath })\le \overline{\eth }(\gimel _{\imath })$. Then

$$\begin{aligned} FHFRWA\left( \mathcal {G} (\gimel _{1}),\mathcal {G} (\gimel _{2}),...,\mathcal {G} (\gimel _{\ell })\right) \le FHFRWA\left( \eth (\gimel _{1}),\eth (\gimel _{2}),...,\eth (\gimel _{\ell })\right) . \end{aligned}$$

(4) **Shiftinvariance:** Consider another FHFRV $\mathcal {G} (\gimel )=\left( \underline{\mathcal {G} }(\gimel ),\overline{ \mathcal {G} }(\gimel )\right) =\left( (\underline{\partial },\underline{d}),( \overline{\partial },\overline{d})\right) .$ Then

$$\begin{aligned}{} & {} FHFRWA\left( \eth (\gimel _{1})\oplus \mathcal {G} (\gimel ),\eth (\gimel _{2})\oplus \mathcal {G} (\gimel ),...,\eth (\gimel _{\ell })\oplus \mathcal {G} (\gimel )\right) = \\{} & {} FHFRWA\left( \eth (\gimel _{1}),\eth (\gimel _{2}),...,\eth (\gimel _{\ell })\right) \oplus \mathcal {G} (\gimel ). \end{aligned}$$

(5) **Homogeneity: **For any real number $\varsigma >0;$

$$\begin{aligned} FHFRWA\left( \varsigma \eth (\gimel _{1}),\varsigma \eth (\gimel _{2}),...,\varsigma \eth (\gimel _{\ell })\right) =\varsigma \cdot FHFRWA\left( \eth (\gimel _{1}),\eth (\gimel _{2}),...,\eth (\gimel _{\ell })\right) . \end{aligned}$$

(6) **Commutativity: **Suppose $\eth ^{^{\prime }}(\gimel _{\imath })=\left( \underline{\eth ^{^{\prime }}}\left( \gimel _{\imath }\right) , \overline{\eth ^{^{\prime }}}(\gimel _{\imath })\right)$ and $\eth (\gimel _{\imath })=(\underline{\eth }(\gimel _{\imath }),\overline{\eth } (\gimel _{\imath })),$ $(\imath =1,2,3,...,\ell )$ is a collection of FHFRVs. Then

$$\begin{aligned} FHFRWA\left( \eth (\gimel _{1}),\eth (\gimel _{2}),...,\eth (\gimel _{\ell })\right) =FHFRWA\left( \eth ^{^{\prime }}(\gimel _{1}),\eth ^{^{\prime }}(\gimel _{2}),...,\eth ^{^{\prime }}(\gimel _{\ell })\right) . \end{aligned}$$

#### Proof

**(1) Idempotency:** As $\eth (\gimel _{\imath })=\mathcal {G} (\gimel )$ (for all $\imath =1,2,3,...,\ell$) where $\mathcal {G} (\gimel _{\imath })=\left( \underline{\mathcal {G} }(\gimel ),\overline{\mathcal {G} }(\gimel )\right) =\left( (\underline{\partial _{\imath }},\underline{d}_{\imath }),(\overline{\partial _{\imath }}, \overline{d}_{\imath })\right) .$

$$\begin{aligned}{} & {} FHFRWA\left( \eth (\gimel _{1}),\eth (\gimel _{2}),...,\eth (\gimel _{\ell })\right) \\= & {} \left( \bigoplus \nolimits _{\imath =1}^{\ell }\propto _{\imath }\underline{\eth }\left( \gimel _{\imath }\right) ,\bigoplus \nolimits _{\imath =1}^{\ell }\propto _{\imath }\overline{\eth }(\gimel _{\imath })\right) \\= & {} \left[ \begin{array}{l} \bigcup \limits _{\underline{\mu _{\imath }}\in \zeta _{h_{\underline{\eth } (\gimel )}}}\root 3 \of {\left( 1-\overset{\ell }{\underset{\imath =1}{\boxtimes }} \left( 1-\left( \underline{\mu _{\imath }}\right) ^{3}\right) ^{\propto _{\imath }}\right) }, \text { }\bigcup \limits _{\underline{{\mathcal {V}} _{\imath }}\in \mathcal {J} _{h_{\underline{\eth } (\gimel )}}}\overset{\ell }{\underset{\imath =1}{\boxtimes }}\left( \underline{{\mathcal {V}} _{\imath }}\right) ^{^{\propto _{\imath }}} \\ \bigcup \limits _{\overline{\mu _{\imath }}\in \zeta _{h_{\overline{\eth }(\gimel )}}}\root 3 \of {\left( 1-\overset{\ell }{\underset{\imath =1}{\boxtimes }}\left( 1-\left( \overline{\mu _{\imath }}\right) ^{3}\right) ^{\propto _{\imath }}\right) },\text { } \bigcup \limits _{\overline{{\mathcal {V}} _{\imath }}\in \mathcal {J} _{h_{\overline{\eth }(\gimel )}}}\overset{\ell }{\underset{\imath =1}{\boxtimes }}\left( \overline{{\mathcal {V}} _{\imath }}\right) ^{^{\propto _{\imath }}} \end{array} \right] , \end{aligned}$$

for all *i*,  $\eth (\gimel _{\imath })=\mathcal {G} (\gimel )=$ $\left( \underline{\mathcal {G} }(\gimel ),\overline{\mathcal {G} }(\gimel )\right) =\left( (\underline{\partial _{\imath }},\underline{d_{\imath }}),(\overline{d}_{\imath }, \overline{e_{\imath }})\right) .$ Therefore,

$$\begin{aligned}= & {} \left[ \begin{array}{l} \bigcup \limits _{\underline{b_{\imath }}\in \zeta _{h_{\underline{\eth }(\gimel )}}}\root 3 \of {\left( 1-\overset{\ell 
}{\underset{\imath =1}{\boxtimes }}\left( 1-\left( \underline{\partial _{\imath }}\right) ^{3}\right) ^{\propto _{\imath }}\right) },\text { } \bigcup \limits _{\underline{d_{\imath }}\in \mathcal {J} _{h_{\underline{\eth }(\gimel )}}}\overset{\ell }{\underset{\imath =1}{\boxtimes }}\left( \underline{d_{\imath }}\right) ^{^{\propto _{\imath }}} \\ \bigcup \limits _{\overline{\partial _{\imath }}\in \zeta _{h_{\overline{\eth }(\gimel )}}}\root 3 \of {\left( 1-\overset{\ell }{\underset{\imath =1}{\boxtimes }}\left( 1-\left( \overline{\partial _{\imath }}\right) ^{3}\right) ^{\propto _{\imath }}\right) },\text { } \bigcup \limits _{\overline{d_{\imath }}\in \mathcal {J} _{h_{\overline{\eth }(\gimel )}}}\overset{\ell }{\underset{\imath =1}{\boxtimes }}\left( \overline{d_{\imath }}\right) ^{^{\propto _{\imath }}} \end{array} \right] . \\= & {} \left[ \left( 1-\left( 1-\underline{\partial _{\imath }}\right) ,\underline{\partial _{\imath }} \right) ,\left( 1-\left( 1-\overline{d}_{\imath }\right) ,\overline{\partial }_{\imath }\right) \right] =\left( \underline{\mathcal {G} }(\gimel ),\overline{\mathcal {G} } (\gimel )\right) =\mathcal {G} (\gimel ). \end{aligned}$$

Hence FHFRWA$$\left( \eth (\gimel _{1}),\eth (\gimel _{2}),...,\eth (\gimel _{\ell })\right) =\mathcal {G} (\gimel ).$$

**(2) Boundedness: **As

$$\begin{aligned} \left( \underline{\eth }\left( \gimel \right) \right) ^{-}= & {} \left[ \left( \underset{\imath }{\min }\{\underline{\mu _{\imath }}\},\underset{\imath }{\max }\ \left\{ \underline{{\mathcal {V}} _{\imath }}\right\} \right) ,\left( \underset{\imath }{\min }\{ \overline{\mu _{\imath }}\},\underset{\imath }{\max }\left\{ \overline{{\mathcal {V}} }_{\imath }\right\} \right) \right] \\ \left( \underline{\eth }\left( \gimel \right) \right) ^{+}= & {} \left[ \left( \underset{\imath }{\max }\{\underline{\mu _{\imath }}\},\underset{\imath }{\min }\ \left\{ \underline{{\mathcal {V}} _{\imath }}\right\} \right) ,\left( \underset{\imath }{\max }\{ \overline{\mu _{\imath }}\},\underset{\imath }{\min }\left\{ \overline{{\mathcal {V}} }_{\imath }\right\} \right) \right] \end{aligned}$$

and $\eth (\gimel _{\imath })=\left[ \left( \underline{\zeta _{\imath }},\underline{ \mathcal {J} _{\imath }}\right) ,\left( \overline{\zeta _{\imath }},\overline{\mathcal {J} }_{\imath }\right) \right] .$ To prove that

$$\begin{aligned} \left( \eth (\gimel )\right) ^{-}\le FHFRWA\left( \eth (\gimel _{1}),\eth (\gimel _{2}),...,\eth (\gimel _{\ell })\right) \le \left( \eth (\gimel )\right) ^{+}. \end{aligned}$$

Since for each $\imath =1,2,3,...,\ell ,$ this implies that

$$\begin{aligned} \underset{\imath }{\min }\{\underline{\mu _{\imath }}\}\le & {} \{\underline{\mu _{\imath }} \}\le \underset{\imath }{\max }\{\underline{\mu _{\imath }}\}\Longleftrightarrow 1- \underset{\imath }{\max }\{\underline{\mu _{\imath }}\}\le 1-\{\underline{\mu _{\imath }} \}\le 1-\{\underline{\mu _{\imath }}\} \\\Longleftrightarrow & {} \overset{\ell }{\underset{\imath =1}{\boxtimes }}\left( 1-\underset{ i}{\max }\{\underline{\mu _{\imath }}\}\right) ^{\propto _{\imath }}\le \overset{\ell }{\underset{ \imath =1}{\boxtimes }}\left( 1-\{\underline{\mu _{\imath }}\}\right) ^{\propto _{\imath }}\le \overset{ n}{\underset{\imath =1}{\boxtimes }}\left( 1-\underset{\imath }{\min }\{\underline{\mu _{\imath }} \}\right) ^{\propto _{\imath }} \\\Longleftrightarrow & {} \left( 1-\underset{\imath }{\max }\{\underline{\mu _{\imath }} \}\right) \le \overset{\ell }{\underset{\imath =1}{\boxtimes }}\left( 1-\{\underline{\mu _{\imath }}\}\right) ^{\propto _{\imath }}\le \left( 1-\underset{\imath }{\min }\{\underline{\mu _{\imath } }\}\right) \\\Longleftrightarrow & {} 1-\left( 1-\underset{\imath }{\min }\{\underline{\mu _{\imath }} \}\right) \le 1-\overset{\ell }{\underset{\imath =1}{\boxtimes }}\left( 1-\{\underline{ \mu _{\imath }}\}\right) ^{\propto _{\imath }}\le 1-\left( 1-\underset{\imath }{\max }\{\underline{ \mu _{\imath }}\}\right) . \end{aligned}$$

Hence

1 $$\begin{aligned} \underset{\imath }{\min }\{\underline{\mu _{\imath }}\}\le 1-\overset{\ell }{\underset{\imath =1}{ \boxtimes }}\left( 1-\{\underline{\mu _{\imath }}\}\right) ^{\propto _{\imath }}\le \underset{\imath }{ \max }\{\underline{\mu _{\imath }}\} \end{aligned}$$

Next for each $\imath =1,2,3,...,\ell$, we have

$$\begin{aligned} \underset{\imath }{\min }\left\{ \underline{{\mathcal {V}} _{\imath }}\right\}\le & {} \left\{ \underline{{\mathcal {V}} _{\imath }}\right\} \le \underset{\imath }{\max }\left\{ \underline{{\mathcal {V}} _{\imath }}\right\} \Longleftrightarrow \overset{\ell }{\underset{\imath =1}{\boxtimes }}\left( \underset{\imath }{\min }\left\{ \underline{{\mathcal {V}} _{\imath }}\right\} \right) ^{^{\propto _{\imath }}}\\\le & {} \overset{\ell }{\underset{\imath =1}{\boxtimes }}\left( \underline{{\mathcal {V}} _{\imath }} \right) ^{^{\propto _{\imath }}}\le \overset{\ell }{\underset{\imath =1}{\boxtimes }}\left( \underset{ \imath }{\max }\left\{ \underline{{\mathcal {V}} _{\imath }}\right\} \right) ^{^{\propto _{\imath }}}. \end{aligned}$$

This follows that

2 $$\begin{aligned} \underset{\imath }{\min }\left\{ \underline{{\mathcal {V}} _{\imath }}\right\} \le \overset{\ell }{ \underset{\imath =1}{\boxtimes }}\left\{ \underline{{\mathcal {V}} _{\imath }}\right\} ^{^{\propto _{\imath }}}\le \underset{\imath }{\max }\left\{ \underline{{\mathcal {V}} _{\imath }}\right\} . \end{aligned}$$

Likewise, we can demonstrate that

3 $$\begin{aligned} \underset{\imath }{\min }\left\{ \overline{\mu _{\imath }}\right\} \le \overset{\ell }{ \underset{\imath =1}{\boxtimes }}\left\{ \overline{\mu _{\imath }}\right\} ^{^{\propto _{\imath }}}\le \underset{\imath }{\max }\left\{ \overline{\mu _{\imath }}\right\} \end{aligned}$$

and

4 $$\begin{aligned} \underset{\imath }{\min }\left\{ \overline{{\mathcal {V}} }_{\imath }\right\} \le \overset{\ell }{ \underset{\imath =1}{\boxtimes }}\left\{ \overline{{\mathcal {V}} }_{\imath }\right\} ^{^{\propto _{\imath }}}\le \underset{\imath }{\max }\left\{ \overline{{\mathcal {V}} }_{\imath }\right\} . \end{aligned}$$

Therefore, based on Equations (1), (2), (3) and (4) we have

$$\begin{aligned} \left( \underline{\eth }\left( \gimel \right) \right) ^{-}=\left[ \left( \underset{\imath }{\min }\{\underline{\mu _{\imath }}\},\underset{\imath }{\max }\left\{ \underline{{\mathcal {V}} _{\imath }}\right\} \right) ,\left( \underset{\imath }{\min }\{\overline{ \mu _{\imath }}\},\underset{\imath }{\max }\left\{ \overline{{\mathcal {V}} }_{\imath }\right\} \right) \right] . \end{aligned}$$

**(3) Monotonicity: **Since $\mathcal {G} (\gimel )=\left( \underline{\mathcal {G} }(\gimel _{\imath }),\overline{\mathcal {G} }(\gimel _{\imath })\right) =\left( \left( \underline{\partial },\underline{d}\right) ,\left( \overline{\partial },\overline{d}\right) \right)$ and $\eth (\gimel _{\imath })=\left( \underline{\eth }\left( \gimel _{\imath }\right) ,\overline{\eth } (\gimel _{\imath })\right)$ to show that $\underline{\mathcal {G} } (\gimel _{\imath })\le \underline{\eth }\left( \gimel _{\imath }\right)$ and $\overline{\mathcal {G} }(\gimel _{\imath })\le \overline{\eth }(\gimel _{\imath })$ (for $\imath =1,2,3,...,\ell$), so

5 $$\begin{aligned} \underline{\partial _{\imath }}\le & {} \underline{\mu _{\imath }}\Rightarrow 1-\underline{\partial _{\imath }} \le 1-\underline{\mu _{\imath }}\Rightarrow \overset{\ell }{\underset{\imath =1}{\boxtimes }} \left( 1-\underline{\mu _{\imath }}\right) ^{\propto _{\imath }}\le \overset{\ell }{\underset{\imath =1}{ \boxtimes }}\left( 1-\underline{\partial _{\imath }}\right) ^{\propto _{\imath }} \nonumber \\\Rightarrow & {} 1-\overset{\ell }{\underset{\imath =1}{\boxtimes }}\left( 1-\underline{\partial _{\imath } }\right) ^{\propto _{\imath }}\le 1-\overset{\ell }{\underset{\imath =1}{\boxtimes }}\left( 1- \underline{\mu _{\imath }}\right) ^{\propto _{\imath }} \end{aligned}$$

next

6 $$\begin{aligned} \underline{d}_{\imath }\ge \underline{{\mathcal {V}} _{\imath }}\Rightarrow \overset{\ell }{\underset{ \imath =1}{\boxtimes }}\underline{d}_{\imath }^{\propto _{\imath }}\ge \overset{\ell }{\underset{\imath =1}{ \boxtimes }}\underline{{\mathcal {V}} _{\imath }}^{\propto _{\imath }}. \end{aligned}$$

Likewise, we can show that

7 $$\begin{aligned} 1-\overset{\ell }{\underset{\imath =1}{\boxtimes }}\left( 1-\overline{\partial }_{\imath }\right) ^{\propto _{\imath }}\le 1-\overset{\ell }{\underset{\imath =1}{\boxtimes }}\left( 1-\overline{\mu _{\imath 
}}\right) ^{\propto _{\imath }} \end{aligned}$$

8 $$\begin{aligned} \overset{\ell }{\underset{\imath =1}{\boxtimes }}\left( \overline{\partial }_{\imath }\right) ^{\propto _{\imath }}\ge \overset{\ell }{\underset{\imath =1}{\boxtimes }}\left( \overline{{\mathcal {V}} } _{\imath }\right) ^{\propto _{\imath }} \end{aligned}$$

Thus, based on the Equations (5), (6), (7) and (8), we get $\underline{ \mathcal {G} }(\gimel _{\imath })\le \underline{\eth }\left( \gimel _{\imath }\right)$ and $\overline{\mathcal {G} }(\gimel _{\imath })\le \overline{ \eth }(\gimel _{\imath }).$ Therefore,

$$\begin{aligned} FHFRWA\left( \mathcal {G} (\gimel _{1}),\mathcal {G} (\gimel _{2}),...,\mathcal {G} (\gimel _{\ell })\right) \le FHFRWA\left( \eth (\gimel _{1}),\eth (\gimel _{2}),...,\eth (\gimel _{\ell })\right) . \end{aligned}$$

**(4) Shiftinvariance: **As $\mathcal {G} (\gimel )=\left( \underline{\mathcal {G} }(\gimel ),\overline{\mathcal {G} }(\gimel )\right) =\left( (\underline{\partial _{\imath }},\underline{d_{\imath }}),(\overline{\partial }_{\imath }, \overline{d}_{\imath })\right)$ is a FHFRV and $\eth (\gimel _{\imath })=\left( \underline{\eth }\left( \gimel _{\imath }\right) ,\overline{\eth }(\gimel _{\imath })\right) =\left[ \left( \underline{\zeta _{\imath }},\underline{\mathcal {J} _{\imath }}\right) ,\left( \overline{\zeta _{\imath }},\overline{\mathcal {J} }_{\imath }\right) \right]$ is the collection of FHFRVs, so

$$\begin{aligned} \eth (\gimel _{1})\oplus \mathcal {G} (\gimel )=\left[ \underline{\eth }\left( \gimel _{1}\right) \oplus \underline{\mathcal {G} }(\gimel ), \overline{\eth }(\gimel _{\imath })\oplus \overline{\mathcal {G} }(\gimel ) \right] . \end{aligned}$$

As

$$\begin{aligned} \left( \left( 1-\left( 1-\underline{\mu _{\imath }}\right) \left( 1-\underline{ d_{\imath }}\right) ,\underline{{\mathcal {V}} _{\imath }}\underline{d_{\imath }}\right) ,\left( 1-\left( 1-\overline{\mu _{\imath }}\right) \left( 1-\overline{d_{\imath }}\right) ,\overline{{\mathcal {V}} }_{\imath }\overline{d_{\imath }}\right) \right) . \end{aligned}$$

Thus, FHFRV $\mathcal {G} (\gimel )=\left( \underline{\mathcal {G} } (\gimel ),\overline{\mathcal {G} }(\gimel )\right) =\left( ( \underline{\partial _{\imath }},\underline{d_{\imath }}),(\overline{\partial _{\imath }},\overline{d} _{\imath })\right) .$ It follows that

$$\begin{aligned}{} & {} FHFRWA\left( \eth (\gimel _{1})\oplus \mathcal {G} (\gimel ),\eth (\gimel _{2})\oplus \mathcal {G} (\gimel ),...,\eth (\gimel _{\ell })\oplus \mathcal {G} (\gimel )\right) \\= & {} \left[ \bigoplus \nolimits _{\imath =1}^{\ell }\propto _{\imath }\underline{\eth }\left( \gimel _{\imath }\right) \oplus \mathcal {G} (\gimel ),\bigoplus \nolimits _{\imath =1}^{\ell }\propto _{\imath }\left( \underline{\eth }(\gimel _{\imath })\oplus \mathcal {G} (\gimel )\right) \right] \\= & {} \left[ \begin{array}{l} \left\{ \begin{array}{l} \bigcup \limits _{\underline{\mu _{\imath }}\in \zeta _{h_{\underline{\eth } (\gimel )}}}\root 3 \of {\left( 1-\overset{\ell }{\underset{\imath =1}{\boxtimes }} \left( 1-\left( \underline{\mu _{\imath }}\right) ^{3}\right) ^{\propto _{\imath }}\left( 1- \underline{\partial _{\imath }}\right) ^{\propto _{\imath }}\right) }, \text { }\bigcup \limits _{\underline{{\mathcal {V}} _{\imath }}\in \mathcal {J} _{h_{\underline{\eth } (\gimel )}}}\overset{\ell }{\underset{\imath =1}{\boxtimes }}\left( \underline{{\mathcal {V}} _{\imath }}\right) ^{^{\propto _{\imath }}}\underline{d}_{\imath }^{^{\propto _{\imath }}} \end{array} \right\} , \\ \left\{ \begin{array}{l} \bigcup \limits _{\overline{\mu _{\imath }}\in \zeta _{h_{\overline{\eth }(\gimel )}}}\root 3 \of {\left( 1-\overset{\ell }{\underset{\imath =1}{\boxtimes }}\left( 1-\left( \overline{\mu _{\imath }}\right) ^{3}\right) ^{\propto _{\imath }}\right) \left( 1-\overline{ \partial _{\imath }}\right) ^{\propto _{\imath }}}, \text { }\bigcup \limits _{\overline{{\mathcal {V}} _{\imath }}\in \mathcal {J} _{h_{\overline{\eth } (\gimel )}}}\underline{d}_{\imath }\overset{\ell }{\underset{\imath =1}{\boxtimes }}\left( \overline{{\mathcal {V}} }_{\imath }\right) ^{^{\propto _{\imath }}} \end{array} \right\} \end{array} \right] \\& =\left[ \begin{array}{l} \left\{ \begin{array}{l} \bigcup \limits _{\underline{\mu _{\imath }}\in \zeta _{h_{\underline{\eth } (\gimel )}}}\root 3 \of {\left( 1-\left( 1-\underline{\partial }\right) \overset{\ell }{ \underset{\imath =1}{\boxtimes }}\left( 1-\left( \underline{\mu _{\imath }}\right) ^{3}\right) ^{\propto _{\imath }}\right) },\text { } \bigcup \limits _{\underline{{\mathcal {V}} _{\imath }}\in \mathcal {J} _{h_{\underline{\eth } (\gimel )}}}\underline{d}\overset{\ell }{\underset{\imath =1}{\boxtimes }}\left( \underline{{\mathcal {V}} _{\imath }}\right) ^{^{\propto _{\imath }}} \end{array} \right\} , \\ \left\{ \begin{array}{l} \bigcup \limits _{\overline{\mu _{\imath }}\in \zeta _{h_{\overline{\eth }(\gimel )}}}\root 3 \of {\left( 1-\overline{\partial }\right) \left( 1-\overset{\ell }{\underset{\imath =1}{\boxtimes }}\left( 1-\left( \overline{\mu _{\imath }}\right) ^{3}\right) ^{\propto _{\imath }}\right) },\text { } \bigcup \limits _{\overline{{\mathcal {V}} _{\imath }}\in \mathcal {J} _{h_{\overline{\eth }(\gimel )}}}\overline{d}\overset{\ell }{\underset{\imath =1}{\boxtimes }}\left( \overline{{\mathcal {V}} } _{\imath }\right) ^{^{\propto _{\imath }}} \end{array} \right\} \end{array} \right] \\ & =\left[ \begin{array}{l} \left\{ \left( \begin{array}{l} \bigcup \limits _{\underline{\mu _{\imath }}\in \zeta _{h_{\underline{\eth } (\gimel )}}}\root 3 \of {\left( 1-\overset{\ell }{\underset{\imath =1}{\boxtimes }} \left( 1-\left( \underline{\mu _{\imath }}\right) ^{3}\right) ^{\propto _{\imath }}\right) }, \text { } \bigcup \limits _{\underline{{\mathcal {V}} _{\imath }}\in \mathcal {J} _{h_{\underline{\eth } (\gimel )}}}\overset{\ell }{\underset{\imath =1}{\boxtimes }}\left( \underline{{\mathcal {V}} _{\imath }}\right) ^{^{\propto _{\imath }}} \end{array} \right) \oplus \left( \underline{\partial _{\imath }},\underline{d}_{\imath }\right) \right\} , \\ \left\{ \left( \begin{array}{l} \bigcup \limits _{\overline{\mu _{\imath }}\in \zeta _{h_{\overline{\eth }(\gimel )}}}\root 3 \of {\left( 1-\overset{\ell }{\underset{\imath =1}{\boxtimes }}\left( 1-\left( \overline{\mu _{\imath }}\right) ^{3}\right) ^{\propto _{\imath }}\right) }, \text { }\bigcup \limits _{\overline{{\mathcal {V}} _{\imath }}\in \mathcal {J} _{h_{\overline{\eth } (\gimel )}}}\overset{\ell }{\underset{\imath =1}{\boxtimes }}\left( \overline{{\mathcal {V}} } _{\imath }\right) ^{^{\propto _{\imath }}} \end{array} \right) \oplus \left( \overline{\partial _{\imath }},\overline{d_{\imath }}\right) \right\} \end{array} \right] \end{aligned}$$

$$\begin{aligned}= & {} \left[ \begin{array}{l} \left( \begin{array}{l} \bigcup \limits _{\underline{\mu _{\imath }}\in \zeta _{h_{\underline{\eth } (\gimel )}}}\root 3 \of {\left( 1-\overset{\ell }{\underset{\imath =1}{\boxtimes }} \left( 1-\left( \underline{\mu _{\imath }}\right) ^{3}\right) ^{\propto 
_{\imath }}\right) }, \text { } \bigcup \limits _{\underline{{\mathcal {V}} _{\imath }}\in \mathcal {J} _{h_{\underline{\eth } (\gimel )}}}\overset{\ell }{\underset{\imath =1}{\boxtimes }}\left( \underline{{\mathcal {V}} _{\imath }}\right) ^{^{\propto _{\imath }}} \end{array} \right) , \\ \left( \begin{array}{l} \bigcup \limits _{\overline{\mu _{\imath }}\in \zeta _{h_{\overline{\eth }(\gimel )}}}\root 3 \of {\left( 1-\overset{\ell }{\underset{\imath =1}{\boxtimes }}\left( 1-\left( \overline{\mu _{\imath }}\right) ^{3}\right) ^{\propto _{\imath }}\right) }, \text { }\bigcup \limits _{\overline{{\mathcal {V}} _{\imath }}\in \mathcal {J} _{h_{\overline{\eth } (\gimel )}}}\overset{\ell }{\underset{\imath =1}{\boxtimes }}\left( \overline{{\mathcal {V}} } _{\imath }\right) ^{^{\propto _{\imath }}} \end{array} \right) \end{array} \right] \oplus \left[ \left( \underline{\partial _{\imath }},\underline{d_{\imath }}\right) ,\left( \overline{\partial _{\imath }},\overline{d_{\imath }}\right) \right] \\= & {} FHFRWA\left( \eth (\gimel _{1}),\eth (\gimel _{2}),...,\eth (\gimel _{\ell })\right) \oplus \mathcal {G} (\gimel ). \end{aligned}$$

**(5) Homogeneity: **For real number $\varsigma >0$ and $\eth (\gimel _{\imath })=\left( \underline{\eth }\left( \gimel _{\imath }\right) , \overline{\eth }(\gimel _{\imath })\right)$ be a FHFRVs. Consider

$$\begin{aligned} \varsigma \eth (\gimel _{\imath })= & {} \left( \varsigma \underline{\eth }\left( \gimel _{\imath }\right) ,\varsigma \overline{\eth }(\gimel _{\imath })\right) \\= & {} \left[ \begin{array}{l} \left\{ \bigcup \limits _{\underline{\mu _{\imath }}\in \zeta _{h_{\underline{\eth } (\gimel )}}}\left( \root 3 \of {\left( 1-\left( 1-\underline{\mu _{\imath }} ^{3}\right) ^{\varsigma }\right) }\right) ,\bigcup \limits _{\underline{{\mathcal {V}} _{\imath }} \in \mathcal {J} _{h_{\underline{\eth }(\gimel )}}}\left( \underline{{\mathcal {V}} _{\imath }} ^{^{\varsigma }}\right) \right\} , \\ \left\{ \bigcup \limits _{\overline{\mu _{\imath }}\in \zeta _{h_{\overline{\eth } (\gimel )}}}\left( \root 3 \of {\left( 1-\left( 1-\overline{\mu _{\imath }} ^{3}\right) ^{\varsigma }\right) }\right) ,\bigcup \limits _{\overline{{\mathcal {V}} _{\imath }} \in \mathcal {J} _{h_{\underline{\eth }(\gimel )}}}\left( \overline{{\mathcal {V}} } _{\imath 
}^{^{^{\varsigma }}}\right) \right\} \end{array} \right] \end{aligned}$$

Now

$$\begin{aligned}{} & {} FHFRWA\left( \varsigma \eth (\gimel _{1}),\varsigma \eth (\gimel _{2}),...,\varsigma \eth (\gimel _{\ell })\right) \\= & {} \left[ \begin{array}{l} \left( \bigcup \limits _{\underline{\mu _{\imath }}\in \zeta _{h_{\underline{\eth } (\gimel )}}}\root 3 \of {\left( 1-\overset{\ell }{\underset{\imath =1}{\boxtimes }} \left( 1-\left( \underline{\mu _{\imath }}\right) ^{3}\right) ^{\varsigma }\right) } ,\bigcup \limits _{\underline{{\mathcal {V}} _{\imath }}\in \mathcal {J} _{h_{\underline{\eth } (\gimel )}}}\overset{\ell }{\underset{\imath =1}{\boxtimes }}\left( \underline{{\mathcal {V}} _{\imath }}\right) ^{\varsigma }\right) , \\ \left( \bigcup \limits _{\overline{\mu _{\imath }}\in \zeta _{h_{\overline{\eth } (\gimel )}}}\root 3 \of {\left( 1-\overset{\ell }{\underset{\imath =1}{\boxtimes }} \left( 1-\left( \overline{\mu _{\imath }}\right) ^{3}\right) ^{\varsigma }\right) } ,\bigcup \limits _{\overline{{\mathcal {V}} _{\imath }}\in \mathcal {J} _{h_{\underline{\eth } (\gimel )}}}\overset{\ell }{\underset{\imath =1}{\boxtimes }}\left( \overline{{\mathcal {V}} } _{\imath }\right) ^{^{\varsigma }}\right) \end{array} \right] \\= & {} \varsigma FHFRWA\left( \eth (\gimel _{1}),\eth (\gimel _{2}),...,\eth (\gimel _{\ell })\right) . \end{aligned}$$

**(6) Commutativity: **Suppose

$$\begin{aligned}{} & {} FHFRWA\left( \eth (\gimel _{1}),\eth (\gimel _{2}),...,\eth (\gimel _{\ell })\right) , \\= & {} \left[ \bigoplus \nolimits _{\imath =1}^{\ell }\varsigma _{\imath }\underline{\eth }(\gimel _{\imath }),\bigoplus \nolimits _{\imath =1}^{\ell }\varsigma _{\imath }\overline{\eth }(\gimel _{\imath }) \right] , \\= & {} \left[ \begin{array}{l} \left( \bigcup \limits _{\underline{\mu _{\imath }}\in \zeta _{h_{\underline{\eth } (\gimel )}}}\root 3 \of {\left( 1-\overset{\ell }{\underset{\imath =1}{\boxtimes }} \left( 1-\left( \underline{\mu _{\imath }}\right) ^{3}\right) ^{\varsigma _{\imath }}\right) },\bigcup \limits _{\underline{{\mathcal {V}} _{\imath }}\in \mathcal 
{J} _{h_{\underline{ \eth }(\gimel )}}}\overset{\ell }{\underset{\imath =1}{\boxtimes }}\left( \underline{ {\mathcal {V}} _{\imath }}\right) ^{\varsigma _{\imath }}\right) , \\ \left( \bigcup \limits _{\overline{\mu _{\imath }}\in \zeta _{h_{\overline{\eth } (\gimel )}}}\root 3 \of {\left( 1-\overset{\ell }{\underset{\imath =1}{\boxtimes }} \left( 1-\left( \overline{\mu _{\imath }}\right) ^{3}\right) ^{\varsigma _{\imath }}\right) },\bigcup \limits _{\overline{{\mathcal {V}} _{\imath }}\in \mathcal {J} _{h_{\underline{\eth } (\gimel )}}}\overset{\ell }{\underset{\imath =1}{\boxtimes }}\left( \overline{{\mathcal {V}} } _{\imath }\right) ^{\varsigma _{\imath }}\right) \end{array} \right] . \end{aligned}$$

Let $\left( \eth ^{^{\prime }}(\gimel _{1}),\eth ^{^{\prime }}(\gimel _{2}),...,\eth ^{^{\prime }}(\gimel _{\ell })\right)$ be a permutation of $\left( \eth (\gimel _{1}),\eth (\gimel _{2}),...,\eth (\gimel _{\ell })\right) .$ Then we have $\eth (\gimel _{\imath })=\eth ^{^{\prime }}(\gimel _{\imath })(\imath =1,2,3,...,\ell )$

$$\begin{aligned}= & {} \left[ \begin{array}{l} \left( \bigcup \limits _{\underline{\mu _{\imath }}\in \zeta _{h_{\underline{\eth } (\gimel )}}}\root 3 \of {\left( 1-\overset{\ell }{\underset{\imath =1}{\boxtimes }} \left( 1-\left( \underline{\mu _{\imath }^{^{\prime }}}\right) ^{3}\right) ^{\varsigma _{\imath }}\right) },\bigcup \limits _{\underline{{\mathcal {V}} _{\imath }}\in \mathcal {J} _{h_{ \underline{\eth }(\gimel )}}}\overset{\ell }{\underset{\imath =1}{\boxtimes }}\left( \underline{{\mathcal {V}} _{\imath }^{^{\prime }}}\right) ^{\varsigma _{\imath }}\right) , \\ \left( \bigcup \limits _{\overline{\mu _{\imath }}\in \zeta _{h_{\overline{\eth } (\gimel )}}}\root 3 \of {\left( 1-\overset{\ell }{\underset{\imath =1}{\boxtimes }} \left( 1-\left( \overline{\mu _{\imath }^{^{\prime }}}\right) ^{3}\right) ^{\varsigma _{\imath }}\right) },\bigcup \limits _{\overline{{\mathcal {V}} _{\imath }}\in \mathcal {J} _{h_{\underline{ \eth }(\gimel )}}}\overset{\ell }{\underset{\imath =1}{\boxtimes }}\left( \overline{ {\mathcal {V}} _{\imath }^{^{\prime }}}\right) ^{\varsigma _{\imath }}\right) \end{array} \right] , \\= & {} \left[ \bigoplus \nolimits _{\imath =1}^{\ell }\varsigma _{\imath }\underline{\eth }^{^{\prime }}(\gimel _{\imath }),\bigoplus \nolimits _{\imath =1}^{\ell }\varsigma _{\imath }\overline{\eth ^{^{\prime }}}(\gimel _{\imath })\right] , \\= & {} FHFRWA\left( \eth ^{^{\prime }}(\gimel _{1}),\eth ^{^{\prime }}(\gimel _{2}),...,\eth ^{^{\prime }}(\gimel _{\ell })\right) . \end{aligned}$$

#### Definition 3.3

Consider the collection $\eth (\gimel _{\imath })=(\underline{\eth } (\gimel _{\imath }),\overline{\eth }(\gimel _{\imath }))$ $(\imath =1,2,3,...,\ell )$ of FHFRVs with weight vector $\mathcal {W}=\left( \propto _{1},\propto _{2},...,\propto _{\ell }\right) ^{T}$ such that $\bigoplus _{\imath =1}^{\ell }\propto _{\imath }=1$ and $0\le$ $\propto _{\imath }\le 1.$ The FHFROWA operator is as follows:

$$\begin{aligned}{} & {} FHFROWA\left( \eth (\gimel _{1}),\eth (\gimel _{2}),...,\eth (\gimel _{\ell })\right) \\= & {} \left( \bigoplus _{\imath =1}^{\ell }\propto _{\imath }\underline{\eth _{\rho _{\imath }}}(\gimel _{\imath }),\bigoplus _{\imath =1}^{\ell }\propto _{\imath }\overline{\eth _{\rho _{\imath }}}(\gimel _{\imath })\right) ,\\{} & {} \left( \begin{array}{l} \left( \bigcup \limits _{\underline{\mu _{\imath }}\in \zeta _{h_{\underline{\eth } (\gimel )}}}\root 3 \of {\left( 1-\overset{k+1}{\underset{\imath =1}{\boxtimes }} \left( 1-\left( \underline{\mu _{\imath }}\right) ^{3}\right) ^{\propto _{\imath }}\right) }, \text { }\bigcup \limits _{\underline{{\mathcal {V}} _{\imath }}\in \mathcal {J} _{h_{\underline{\eth } (\gimel )}}}\overset{k+1}{\underset{\imath =1}{\boxtimes }}\left( \underline{{\mathcal {V}} _{\imath }}\right) ^{^{\propto _{\imath }}}\right) \\ \left( \bigcup \limits _{\overline{\mu _{\imath }}\in \zeta _{h_{\overline{\eth } (\gimel )}}}\root 3 \of {\left( 1-\overset{k+1}{\underset{\imath =1}{\boxtimes }} \left( 1-\left( \overline{\mu _{\imath }}\right) ^{3}\right) ^{\propto _{\imath }}\right) }, \text { }\bigcup \limits _{\overline{{\mathcal {V}} _{\imath }}\in \mathcal {J} _{h_{\overline{\eth } (\gimel )}}}\overset{k+1}{\underset{\imath =1}{\boxtimes }}\left( \overline{{\mathcal {V}} _{\imath }}\right) ^{^{\propto _{\imath }}}\right) \end{array} \right) . \end{aligned}$$

#### Theorem 3

Let $\eth (\gimel _{\imath })=(\underline{\eth }(\gimel _{\imath }),\overline{ \eth }(\gimel _{\imath }))$ $(\imath =1,2,3,...,\ell )$ be the collection of FHFRVs with weight vectors $\mathcal {W}=\left( \propto _{1},\propto _{2},...,\propto _{\ell }\right) ^{T}.$ Then FHFROWA operator is given as:

$$\begin{aligned}{} & {} FHFROWA\left( \eth (\gimel _{1}),\eth (\gimel _{2}),...,\eth (\gimel _{\ell })\right) \\= & {} \left( \bigoplus _{\imath =1}^{\ell }\propto _{\imath }\underline{\eth _{\rho _{\imath }}}(\gimel _{\imath }),\bigoplus _{\imath =1}^{\ell }\propto _{\imath }\overline{\eth _{\rho _{\imath }}}(\gimel _{\imath })\right) \\= & {} \left[ \begin{array}{l} \left( \bigcup \limits _{\underline{\mu _{\imath }}\in \zeta _{h_{\underline{\eth } (\gimel )}}}\root 3 \of {\left( 1-\overset{\ell }{\underset{\imath =1}{\boxtimes }} \left( 1-\left( \underline{\mu _{\rho _{\imath }}}\right) ^{3}\right) ^{\propto _{\imath }}\right) },\bigcup \limits _{\underline{{\mathcal {V}} _{\imath }}\in \mathcal {J} _{h_{ \underline{\eth }(\gimel )}}}\overset{\ell }{\underset{\imath =1}{\boxtimes }}\left( \underline{{\mathcal {V}} _{\rho _{\imath }}}\right) ^{^{\propto _{\imath }}}\right) \\ \left( \bigcup \limits _{\overline{\mu _{\imath }}\in \zeta _{h_{\overline{\eth } (\gimel )}}}\root 3 \of {\left( 1-\overset{\ell }{\underset{\imath =1}{\boxtimes }} \left( 1-\left( \overline{\mu _{\rho _{\imath }}}\right) ^{3}\right) ^{\propto _{\imath }}\right) },\bigcup \limits _{\overline{{\mathcal {V}} _{\imath }}\in \mathcal {J} _{h_{\overline{ \eth }(\gimel )}}}\overset{\ell }{\underset{\imath =1}{\boxtimes }}\left( \overline{ {\mathcal {V}} _{\rho _{\imath }}}\right) ^{^{\propto _{\imath }}}\right) \end{array} \right] , \end{aligned}$$

where $\eth _{\rho }(\gimel _{\imath })=(\underline{\eth _{\rho _{\imath }}} (\gimel _{\imath }),\overline{\eth _{\rho _{\imath }}}(\gimel _{\imath }))$ demonstrates the highest permutation value from a collection of FHFRVs

#### Proof

This proof follows the proof of Theorem-1.

#### Theorem 4

Let $\eth (\gimel _{\imath })=(\underline{\eth }(\gimel _{\imath }),\overline{ \eth }(\gimel _{\imath }))$ $(\imath =1,2,3,...,\ell )$ be a collection of FHFRVs and $\mathcal {W}=\left( \propto _{1},\propto _{2},...,\propto _{\ell }\right) ^{T}$ is a weight vector such that $\bigoplus _{\imath =1}^{\ell }\propto _{\imath }=1$ and $0\le$ $\propto _{\imath }\le 1.$ The FHFROWA operator needs to satisfy all of the following conditions:

**(1) Idempotency: **If $\eth (\gimel _{\imath })=\mathcal {G} (\gimel )$ for $\imath =1,2,3,...,\ell$ where $\mathcal {G} (\gimel )=\left( \underline{\mathcal {G} }(\gimel ),\overline{\mathcal {G} }(\gimel )\right) =\left( (\underline{\partial },\underline{d}),(\overline{\partial },\overline{d} )\right) ,$ then

$$\begin{aligned} FHFROWA\left( \eth (\gimel _{1}),\eth (\gimel _{2}),...,\eth (\gimel _{\ell })\right) =\mathcal {G} (\gimel ). \end{aligned}$$

**(2) Boundedness: **Let $\left( \eth (\gimel )\right) ^{-}=\left( \underset{\imath }{\min }\underline{\eth }\left( \gimel _{\imath }\right) ,\underset{ \imath }{\max }\overline{\eth }(\gimel _{\imath })\right)$ and $\left( \eth (\gimel )\right) ^{+}=$ $\left( \underset{\imath }{\max }\underline{\eth } \left( \gimel _{\imath }\right) ,\underset{\imath }{\min }\overline{\eth } (\gimel _{\imath })\right) .$ Then

$$\begin{aligned} \left( \eth (\gimel )\right) ^{-}\le FHFROWA\left( \eth (\gimel _{1}),\eth (\gimel _{2}),...,\eth (\gimel _{\ell })\right) \le \left( \eth (\gimel )\right) ^{+}. \end{aligned}$$

**(3) Monotonicity: **Suppose $\mathcal {G} (\gimel )=\left( \underline{\mathcal {G} }(\gimel _{\imath }),\overline{\mathcal {G} } (\gimel _{\imath })\right) (\imath =1,2,...,n)$ is another collection of FHFRVs such that $\underline{\mathcal {G} }(\gimel _{\imath })\le \underline{\eth } \left( \gimel _{\imath }\right)$ and $\overline{\mathcal {G} }(\gimel _{\imath })\le \overline{\eth }(\gimel _{\imath })$. Then

$$\begin{aligned} FHFROWA\left( \mathcal {G} (\gimel _{1}),\mathcal {G} (\gimel _{2}),...,\mathcal {G} (\gimel _{\ell })\right) \le FHFROWA\left( \eth (\gimel _{1}),\eth (\gimel _{2}),...,\eth (\gimel _{\ell })\right) . \end{aligned}$$

**(4) Shiftinvariance:** Consider another FHFRV $\mathcal {G} (\gimel )=\left( \underline{\mathcal {G} }(\gimel ),\overline{ \mathcal {G} }(\gimel )\right) =\left( (\underline{\partial },\underline{d}),( \overline{\partial },\overline{d})\right) .$ Then

$$\begin{aligned}{} & {} FHFROWA\left( \eth (\gimel _{1})\oplus \mathcal {G} (\gimel ),\eth (\gimel _{2})\oplus \mathcal {G} (\gimel ),...,\eth (\gimel _{\ell })\oplus \mathcal {G} (\gimel )\right) \\= & {} FHFROWA\left( \eth (\gimel _{1}),\eth (\gimel _{2}),...,\eth (\gimel _{\ell })\right) \oplus \mathcal {G} (\gimel ). \end{aligned}$$

**(5) Homogeneity:** For any real number $\varsigma >0;$

$$\begin{aligned} FHFROWA\left( \varsigma \eth (\gimel _{1}),\varsigma \eth (\gimel _{2}),...,\varsigma \eth (\gimel _{\ell })\right) =\varsigma \cdot FHFROWA\left( \eth (\gimel _{1}),\eth (\gimel _{2}),...,\eth (\gimel _{\ell })\right) . \end{aligned}$$

**(6) Commutativity: **Suppose $\eth ^{^{\prime }}(\gimel _{\imath })=\left( \underline{\eth ^{^{\prime }}}\left( \gimel _{\imath }\right) , \overline{\eth ^{^{\prime }}}(\gimel _{\imath })\right)$ and $\eth (\gimel _{\imath })=(\underline{\eth }(\gimel _{\imath }),\overline{\eth } (\gimel _{\imath })),$ $(\imath =1,2,3,...,\ell )$ is any FHFRVs. Then

$$\begin{aligned} FHFROWA\left( \eth (\gimel _{1}),\eth (\gimel _{2}),...,\eth (\gimel _{\ell })\right) =FHFROWA\left( \eth ^{^{\prime }}(\gimel _{1}),\eth ^{^{\prime }}(\gimel _{2}),...,\eth ^{^{\prime }}(\gimel _{\ell })\right) . \end{aligned}$$

#### Proof

The proof is similar to the proof of Theorem-2.

#### Definition 3.4

Let $\eth (\gimel _{\imath })=(\underline{\eth }(\gimel _{\imath }),\overline{ \eth }(\gimel _{\imath }))$ $(\imath =1,2,3,...,\ell )$ be the collection of FHFRVs and $\mathcal {W}=\left( \propto _{1},\propto _{2},...,\propto _{\ell }\right) ^{T}$ is a weights vector such that $\bigoplus _{\imath =1}^{\ell }\propto _{\imath }=1$ and $0\le$ $\propto _{\imath }\le 1.$ Let $\varrho =\left( \varrho _{1},\varrho _{2},...,\varrho _{\ell }\right) ^{T}$ such that $\bigoplus _{\imath =1}^{\ell }\varrho _{\imath }=1$ and $0\le$ $\varrho _{\imath }\le 1$ be the weight vectors of specified collection of FHFRVs. Then FHFRHWA operator is given by:

$$\begin{aligned} FHFRHWA\left( \eth (\gimel _{1}),\eth (\gimel _{2}),...,\eth (\gimel _{\ell })\right) =\left( \bigoplus _{\imath =1}^{\ell }\varrho _{\imath }\underline{ \widetilde{\eth }_{\rho }}(\gimel _{\imath }),\bigoplus _{\imath =1}^{\ell }\varrho _{\imath } \overline{\widetilde{\eth }_{\rho }}(\gimel _{\imath })\right) . \end{aligned}$$

#### Theorem 5

Let $\eth (\gimel _{\imath })=(\underline{\eth }(\gimel _{\imath }),\overline{ \eth }(\gimel _{\imath }))$ $(\imath =1,2,3,...,\ell )$ be the collection of FHFRVs and $\mathcal {W}=\left( \propto _{1},\propto _{2},...,\propto _{\ell }\right) ^{T},$ be a weight vector such that $\bigoplus _{\imath =1}^{\ell }\propto _{\imath }=1$ and $0\le$ $\propto _{\imath }\le 1.$ Let $\varrho =\left( \varrho _{1},\varrho _{2},...,\varrho _{\ell }\right) ^{T}$ be the collection of FHFRVs with the properties that $\bigoplus _{\imath =1}^{\ell }\varrho _{\imath }=1$ and $0\le$ $\varrho _{\imath }\le 1$, then the FHFRHWA operator is characterized as:

$$\begin{aligned}{} & {} FHFRHWA\left( \eth (\gimel _{1}),\eth (\gimel _{2}),...,\eth (\gimel _{\ell })\right) \\= & {} \left( \bigoplus _{\imath =1}^{\ell }\varrho _{\imath }\underline{\widetilde{\eth }_{\rho }} (\gimel _{\imath }),\bigoplus _{\imath =1}^{\ell }\varrho _{\imath }\overline{\widetilde{\eth } _{\rho }}(\gimel _{\imath })\right) \\= & {} \left( \begin{array}{l} \left\{ \bigcup \limits _{\underline{\mu _{\imath }}\in \zeta _{h_{\underline{\eth } (\gimel )}}}\root 3 \of {\left( 1-\overset{\ell }{\underset{\imath =1}{\boxtimes }} \left( 1-\left( \underline{\widetilde{\mu }_{\rho _{\imath }}}\right) ^{3}\right) ^{\varrho _{\imath }}\right) },\bigcup \limits _{\underline{{\mathcal {V}} _{\imath }} \in \mathcal {J} _{h_{\underline{\eth }(\gimel )}}}\overset{\ell }{\underset{\imath =1}{ \boxtimes }}\left( \underline{\widetilde{{\mathcal {V}} }_{\rho _{\imath }}}\right) ^{\varrho _{\imath }}\right\} , \\ \left\{ \bigcup \limits _{\overline{\mu _{\imath }}\in \zeta _{h_{\overline{\eth } (\gimel )}}}\root 3 \of {\left( 1-\overset{\ell }{\underset{\imath =1}{\boxtimes }} \left( 1-\left( \overline{\widetilde{\mu }_{\rho _{\imath }}}\right) ^{3}\right) ^{\varrho _{\imath }}\right) },\text { }\bigcup \limits _{\overline{{\mathcal {V}} _{\imath }}\in \mathcal {J} _{h_{\overline{\eth }(\gimel )}}}\overset{\ell }{\underset{\imath =1}{\boxtimes }} \left( \overline{\widetilde{{\mathcal {V}} }_{\rho _{\imath }}}\right) ^{^{\varrho _{\imath }}}\right\} \end{array} \right) , \end{aligned}$$

where $\widetilde{\eth _{\rho }}(\gimel _{\imath })=n\propto _{\imath }\eth (\gimel _{\imath })=(n\propto _{\imath }\underline{\eth }(\gimel _{\imath }),n\propto _{\imath }\overline{\eth } (\gimel _{\imath }))$ indicates the superior permutation value from the set of FHFRVs, and *n* denotes the balancing coefficient.

#### Proof

This proof is similar to the proof of Theorem-1.

#### Theorem 6

Let $\eth (\gimel _{\imath })=(\underline{\eth }(\gimel _{\imath }),\overline{ \eth }(\gimel _{\imath }))$ $(\imath =1,2,3,...,\ell )$ be the collection of FHFRVs and $\mathcal {W}=\left( \propto _{1},\propto _{2},...,\propto _{\ell }\right) ^{T}$ be a weight vector such that $\bigoplus _{\imath =1}^{\ell }\propto _{\imath }=1$ and $0\le$ $\propto _{\imath }\le 1.$ Then FHFRHWA operator must accomplish the following conditions:

**(1) Idempotency: **If $\eth (\gimel _{\imath })=\mathcal {G} (\gimel )$ for $\imath =1,2,3,...,\ell$ where $\mathcal {G} (\gimel )=\left( \underline{\mathcal {G} }(\gimel ),\overline{\mathcal {G} }(\gimel )\right) =\left( (\underline{\partial },\underline{d}),(\overline{\partial },\overline{d} )\right) ,$ then

$$\begin{aligned} FHFRHWA\left( \eth (\gimel _{1}),\eth (\gimel _{2}),...,\eth (\gimel _{\ell })\right) =\mathcal {G} (\gimel ). \end{aligned}$$

**(2) Boundedness: **Let $\left( \eth (\gimel )\right) ^{-}=\left( \underset{\imath }{\min }\underline{\eth }\left( \gimel _{\imath }\right) ,\underset{ \imath }{\max }\overline{\eth }(\gimel _{\imath })\right)$ and $\left( \eth (\gimel )\right) ^{+}=$ $\left( \underset{\imath }{\max }\underline{\eth } \left( \gimel _{\imath }\right) ,\underset{\imath }{\min }\overline{\eth } (\gimel _{\imath })\right) .$ Then

$$\begin{aligned} \left( \eth (\gimel )\right) ^{-}\le FHFRHWA\left( \eth (\gimel _{1}),\eth (\gimel _{2}),...,\eth (\gimel _{\ell })\right) \le \left( \eth (\gimel )\right) ^{+}. \end{aligned}$$

**(3) Monotonicity: **Suppose $\mathcal {G} (\gimel )=\left( \underline{\mathcal {G} }(\gimel _{\imath }),\overline{\mathcal {G} } (\gimel _{\imath })\right) (\imath =1,2,...,n)$ is collection of FHFRVs such that $\underline{\mathcal {G} }(\gimel _{\imath })\le \underline{\eth } \left( \gimel _{\imath }\right)$ and $\overline{\mathcal {G} }(\gimel _{\imath })\le \overline{\eth }(\gimel _{\imath })$. Then

$$\begin{aligned} FHFRHWA\left( \mathcal {G} (\gimel _{1}),\mathcal {G} (\gimel _{2}),...,\mathcal {G} (\gimel _{\ell })\right) \le FHFRHWA\left( \eth (\gimel _{1}),\eth (\gimel _{2}),...,\eth (\gimel _{\ell })\right) . \end{aligned}$$

**(4) Shiftinvariance:** Consider FHFRVs $\mathcal {G} (\gimel )=\left( \underline{\mathcal {G} }(\gimel ),\overline{ \mathcal {G} }(\gimel )\right) =\left( (\underline{\partial },\underline{d}),( \overline{\partial },\overline{d})\right) .$ Then

$$\begin{aligned}{} & {} FHFRHWA\left( \eth (\gimel _{1})\oplus \mathcal {G} (\gimel ),\eth (\gimel _{2})\oplus \mathcal {G} (\gimel ),...,\eth (\gimel _{\ell })\oplus \mathcal {G} (\gimel )\right) = \\{} & {} FHFRHWA\left( \eth (\gimel _{1}),\eth (\gimel _{2}),...,\eth (\gimel _{\ell })\right) \oplus \mathcal {G} (\gimel ). \end{aligned}$$

**(5) Homogeneity: **For any real number $\varsigma >0;$

$$\begin{aligned} FHFRHWA\left( \varsigma \eth (\gimel _{1}),\varsigma \eth (\gimel _{2}),...,\varsigma \eth (\gimel _{\ell })\right) =\varsigma \cdot FHFRHWA\left( \eth (\gimel _{1}),\eth (\gimel _{2}),...,\eth (\gimel _{\ell })\right) . \end{aligned}$$

**(6) Commutativity: **Suppose $\eth ^{^{\prime }}(\gimel _{\imath })=\left( \underline{\eth ^{^{\prime }}}\left( \gimel _{\imath }\right) , \overline{\eth ^{^{\prime }}}(\gimel _{\imath })\right)$ and $\eth (\gimel _{\imath })=(\underline{\eth }(\gimel _{\imath }),\overline{\eth } (\gimel _{\imath })),$ $(\imath =1,2,3,...,\ell )$ is a collection of FHFRVs. Then

$$\begin{aligned} FHFRHWA\left( \eth (\gimel _{1}),\eth (\gimel _{2}),...,\eth (\gimel _{\ell })\right) =FHFRHWA\left( \eth ^{^{\prime }}(\gimel _{1}),\eth ^{^{\prime }}(\gimel _{2}),...,\eth ^{^{\prime }}(\gimel _{\ell })\right) . \end{aligned}$$

#### Proof

This proof is similar to the proof of Theorem-2.

## Multi-attribute decision making framework

In this part, we provide an approach for dealing with uncertainty in MCGDM employing FHFR information. Assume a DM problem having $\left\{ A _{1},A _{2},...,A _{\ell }\right\}$ a set of *n* alternatives and $\left\{ c_{1},c_{2},...,c_{\ell }\right\}$ is a set of attributes along a weight vector $\mathcal {W}=\left( \propto _{1},\propto _{2},...,\propto _{\ell }\right) ^{T}$ that is, $\propto _{\imath }\in [0,1]$ , $\bigoplus _{\imath =1}^{\ell }\propto _{\imath }=1.$ Suppose ${\left\{ \mathring{\mathcal {D}}_{1},\mathring{\mathcal {D}}_{2},...,\mathring{\mathcal {D}}_{\hat{\jmath } }\right\} }$ is a collection of decision makers and  is the weight vector of decision makers such that  $\bigoplus _{\imath =1}^{\ell }\daleth _{\imath }=1$. To assess the trustworthiness of k$^{th}$ alternative $A _{\imath }$ under the attribute $c_{\imath },$ the matrix for expert evaluation is outlined as follows:

$$\begin{aligned} M= & {} \left[ \overline{\eth }(\gimel _{\imath j}^{\hat{\jmath }})\right] _{m\times n} \\= & {} \left[ \begin{array}{cccc} \left( \underline{\eth }(\gimel _{11}),\overline{\eth }(\gimel _{11})\right) &{} \left( \underline{\eth }(\gimel _{12}),\overline{\eth } (\gimel _{12})\right) &{} \cdots &{} \left( \underline{\eth } (\gimel _{1j}),\overline{\eth }(\gimel _{1j})\right) \\ \left( \underline{\eth }(\gimel _{21}),\overline{\eth }(\gimel _{21})\right) &{} \left( \underline{\eth }(\gimel _{22}),\overline{\eth } (\gimel _{22})\right) &{} \cdots &{} \left( \underline{\eth } (\gimel _{2j}),\overline{\eth }(\gimel _{2j})\right) \\ \left( \underline{\eth }(\gimel _{31}),\overline{\eth }(\gimel _{31})\right) &{} \left( \underline{\eth }(\gimel _{32}),\overline{\eth } (\gimel _{32})\right) &{} \cdots &{} \left( \underline{\eth } (\gimel _{3j}),\overline{\eth }(\gimel _{3j})\right) \\ \vdots &{} \vdots &{} \ddots &{} \vdots \\ \left( \underline{\eth }(\gimel _{\imath 1}),\overline{\eth }(\gimel _{\imath 1})\right) &{} \left( \underline{\eth }(\gimel _{\imath 2}),\overline{\eth } (\gimel _{\imath 2})\right) &{} \cdots &{} \left( \underline{\eth } (\gimel _{\imath j}),\overline{\eth }(\gimel _{\imath j})\right) \end{array} \right] , \end{aligned}$$

where $\underline{\eth }(\gimel )=\left\{ \left\langle \jmath ,\zeta _{h_{ \underline{\eth }(\gimel )}}(\jmath ),\mathcal {J} _{h_{\underline{\eth } (\gimel )}}(\jmath )\right\rangle |\jmath \in \mathcal {U} \right\}$ and $\overline{\eth }(\gimel _{\imath j})=\left\{ \left\langle \jmath ,\zeta _{h_{\overline{\eth }(\gimel )}}(\jmath ),\mathcal {J} _{h_{\overline{\eth } (\gimel )}}(\jmath )\right\rangle |\jmath \in \mathcal {U} \right\}$ such that $0\le \left( \min (\zeta _{h_{\underline{\eth }(\gimel )}}(\jmath )\right) ^{3}+\left( \max (\mathcal {J} _{h_{\underline{\eth }(\gimel )}}(\jmath ))\right) ^{3}\le 1$ and $0\le \left( \max (\zeta _{h_{\overline{\eth }(\gimel )}}(\jmath ))\right) ^{3}+\left( \min (\mathcal {J} _{h_{\overline{\eth }(\gimel )}}(\jmath ))\right) ^{3}\le 1$ are the FHFR values. The following are the main steps for MAGDM:

Step-1: Establish the expert evaluation matrices as follows:
  $$\begin{aligned} \left( E\right) ^{_{\hat{\jmath }}}=\left[ \begin{array}{cccc} \left( \underline{\eth }(\gimel _{11}^{_{\hat{\jmath }}}),\overline{\eth } (\gimel _{11}^{_{\hat{\jmath }}})\right) &{} \left( \underline{\eth } (\gimel _{12}^{_{\hat{\jmath }}}),\overline{\eth }(\gimel _{12}^{_{ \hat{\jmath }}})\right) &{} \cdots &{} \left( \overline{\eth }(\gimel _{1j}^{_{\hat{\jmath }}}),\overline{\eth }(\gimel _{1j}^{_{\hat{\jmath } }})\right) \\ \left( \underline{\eth }(\gimel _{21}^{_{\hat{\jmath }}}),\overline{\eth } (\gimel _{21}^{_{\hat{\jmath }}})\right) &{} \left( \underline{\eth } (\gimel _{22}^{_{\hat{\jmath }}}),\overline{\eth }(\gimel _{22}^{_{ \hat{\jmath }}})\right) &{} \cdots &{} \left( \underline{\eth }(\gimel _{2j}^{_{\hat{\jmath }}}),\overline{\eth }(\gimel _{2j}^{_{\hat{\jmath } }})\right) \\ \left( \underline{\eth }(\gimel _{31}^{_{\hat{\jmath }}}),\overline{\eth } (\gimel _{31}^{_{\hat{\jmath }}})\right) &{} \left( \underline{\eth } (\gimel _{32}^{_{\hat{\jmath }}}),\overline{\eth }(\gimel _{32}^{_{ \hat{\jmath }}})\right) &{} \cdots &{} \left( \underline{\eth }(\gimel _{3j}^{_{\hat{\jmath }}}),\overline{\eth }(\gimel _{3j}^{_{\hat{\jmath } }})\right) \\ \vdots &{} \vdots &{} \ddots &{} \vdots \\ \left( \underline{\eth }(\gimel _{i1}^{_{\hat{\jmath }}}),\overline{\eth } (\gimel _{\imath 1}^{_{\hat{\jmath }}})\right) &{} \left( \underline{\eth } (\gimel _{\imath 2}^{_{\hat{\jmath }}}),\overline{\eth }(\gimel _{\imath 2}^{_{ \hat{\jmath }}})\right) &{} \cdots &{} \left( \underline{\eth }(\gimel _{\imath j}^{_{\hat{\jmath }}}),\overline{\eth }(\gimel _{\imath j}^{_{\hat{\jmath } }})\right) \end{array}\right] \end{aligned}$$
  where $\hat{\jmath }$ represents the number of experts.
Step-2: Examine the normalised expert matrices $\left( N\right) ^{ \hat{\jmath }},$ as
  $$\begin{aligned} \left( N\right) ^{\hat{\jmath }}=\left\{ \begin{array}{ccc} \eth (\gimel _{\imath j})=\left( \underline{\eth }\left( \gimel _{\imath j}\right) ,\overline{\eth }\left( \gimel _{\imath j}\right) \right) =\left( \left( \underline{\mu _{\imath j}},\underline{{\mathcal {V}} _{\imath j}}\right) ,\left( \overline{ \mu _{\imath j}},\overline{{\mathcal {V}} _{\imath j}}\right) \right) &{} \text {if} &{} \text {for benefit} \\ \left( \eth (\gimel _{\imath j})\right) ^{c}=\left( \left( \underline{\eth } \left( \gimel _{\imath j}\right) \right) ^{c},\left( \overline{\eth }\left( \gimel _{\imath j}\right) \right) ^{c}\right) =\left( \left( \underline{{\mathcal {V}} _{\imath j}},\underline{\mu _{\imath j}}\right) ,\left( \overline{{\mathcal {V}} _{\imath j}},\overline{ \mu _{\imath j}}\right) \right) &{} \text {if} &{} \text {for cost } \end{array} \right. \end{aligned}$$
Step-3: Employing a FHFRWA aggregation operator, compute the FHFR collected information from DMs.
  $$\begin{aligned}{} & {} FHFRWA\left( \eth (\gimel _{1}),\eth (\gimel _{2}),...,\eth (\gimel _{\ell })\right) \\= & {} \left( \bigoplus _{\imath =1}^{\ell }\propto _{\imath }\underline{\eth }(\gimel _{\imath }),\bigoplus _{\imath =1}^{\ell }\propto _{\imath }\overline{\eth }(\gimel _{\imath })\right) \\= & {} \left[ \begin{array}{l} \bigcup \limits _{\underline{\mu _{\imath }}\in \zeta _{h_{\underline{\eth } (\gimel )}}}\root 3 \of {\left( 1-\overset{\ell }{\underset{\imath =1}{\boxtimes }} \left( 1-\left( \underline{\mu _{\imath }}\right) ^{3}\right) ^{\propto _{\imath }}\right) }, \text { }\bigcup \limits _{\underline{{\mathcal {V}} _{\imath }}\in \mathcal {J} _{h_{\underline{\eth } (\gimel )}}}\overset{\ell }{\underset{\imath =1}{\boxtimes }}\left( \underline{{\mathcal {V}} _{\imath }}\right) ^{^{\propto _{\imath }}} \\ \bigcup \limits _{\overline{\mu _{\imath }}\in \zeta _{h_{\overline{\eth }(\gimel )}}}\root 3 \of {\left( 1-\overset{\ell }{\underset{\imath =1}{\boxtimes }}\left( 1-\left( \overline{\mu _{\imath }}\right) ^{3}\right) ^{\propto _{\imath }}\right) },\text { } \bigcup \limits _{\overline{{\mathcal {V}} _{\imath }}\in \mathcal {J} _{h_{\overline{\eth }(\gimel )}}}\overset{\ell }{\underset{\imath =1}{\boxtimes }}\left( \overline{{\mathcal {V}} _{\imath }}\right) ^{^{\propto _{\imath }}} \end{array} \right] \end{aligned}$$
Step-4: Utilizing the suggested aggregation information, examine the aggregated FHFRVs for each alternative in the context of specified set of attributes/criteria.
Step-5: Evaluate the ranking of alternatives based on the score function outlined in the following way:
  $$\begin{aligned} \Game (\eth (\gimel ))=\frac{1}{4}\left( \begin{array}{l} 2+\frac{1}{M_{\mathcal {R} }}\bigoplus \nolimits _{\mu _{\imath }\in \zeta _{h_{\underline{ \eth }(\gimel )}}}(\underline{\mu _{\imath }})+\frac{1}{N_{\mathcal {R} }} \bigoplus \nolimits _{\mu _{\imath }\in \zeta _{h_{\overline{\eth }(\gimel )}}}( \overline{\mu _{\imath }}) \\ \frac{1}{M_{\mathcal {R} }}\bigoplus \nolimits _{\underline{{\mathcal {V}} _{\imath }}\in \mathcal {J} _{h_{ \underline{\eth }(\gimel )}}}(\underline{{\mathcal {V}} _{\imath }})-\frac{1}{ M_{\mathcal {R} }}\bigoplus \nolimits _{\overline{{\mathcal {V}} _{\imath }}\in \mathcal {J} _{h_{\overline{ \eth }(\gimel )}}}(\overline{{\mathcal {V}} _{\imath }}) \end{array} \right) , \end{aligned}$$
Step-6: Rank each possible scoring alternative in decreasing order from highest to lowest. The alternative that provides a higher value is considered to be the ideal alternative.

## Numerical implementation of the MCGDM framework

This part explores a real-world application of the GSS in the chemical processing industries in order to demonstrate the trustworthiness, supremacy, and precision of the suggested aggregation operators.

### Case study (green supplier selection in industrial systems)

The selection of suppliers has recently risen as one of the important responsibilities of management, as well as one of the most vital and intricate concerns they must address. In addition, GSS performance measures must be taken into account throughout the supplier selection process, and GSS decision-making in the chemical process industry has received relatively significant attention. Due to the rising consumption levels, the GSS has become the most crucial component for environmental conservation and sustainable growth. Since the previous couple of decades, environmental concerns have increased and spread quicker than a wildfire, from nation to region to worldwide territory, which is a significant contributor to rising temperatures and climate variability. Furthermore, the depletion of natural resources and air pollution have a negative impact on the fauna and flora, as well as human life through the infectious illnesses they aggravate, such as diabetes and cardiovascular disease, brain hemorrhage, lung cancer, and chronic obstructive pulmonary disorder, intestinal parasites, typhoid fever, Hepatitis, Cholera, and water-borne diseases. While the GSS idea is used to alleviate potential ecological impacts and control air, water, and waste contaminants through the adoption of environmentally sustainable industry operations. This research aims to give a comprehensive framework for choosing green suppliers by including both economic and environmental factors. The following are the most important attributes for selecting the ideal green supplier:

$(c_{1})$ Cost: Cost may be considered as a significant factor in supplier selection decisions. Appropriate suppliers may minimize costs and give purchasers with enhanced market skills. Cost involves transportation, manufacturing, inventory, energy, maintenance, inspecting expenses, and safety expenditure. In addition to waste disposal expenses as an ecological component and reducing costs capability.

$(c_{2})$ Quality: The administration must address quality assurance and procedure improvement to enhance quality performance. Quality management may fulfil consumer needs for efficient resource usage and aligns with an organisational objectives. Consideration is given to whole management of quality and reliability certifications such as ISO 9000, BS 5750, and EN 29000. Low toxicity and consumer rejection may also indicate quality. Firms may accomplish this objective by fast response, minimal wastage, high production, low inventories, no damage, few faults, and so forth.

$(c_{3})$ Green products: In recent years, there has been a greater focus on green competence among customers and suppliers, which has significant implications and enhances brand reputation. Green packaging is a form of packaging that tries to preserve the natural environment by employing recyclable or reusable, efficient and environmentally materials.

$(c_{4})$ Environmental management: The objectives of environmental sustainable approaches are to persuade businesses to alleviate the negative effects of production on the ecology and to make consumers more environmentally conscious, therefore, influencing the decision-making of industries. Environment-related certifications such as ISO 14000, green manufacturing management, an internal control mechanism, and low carbon initiatives are the primary indications of sustainable development.

**The evaluation procedure for ideal green supplier selection:** Assuming an industry desires to assess the framework for selecting green product suppliers. They will appoint a panel of professionals to evaluate a suitable supplier. Let $\left\{ A_{1},A_{2},A_{3},A_{4}\right\}$ be the set of four alternatives for supplier, and the penal will choose the optimal one. Let $\left\{ c _{1},c _{2},c _{3},c _{4}\right\}$ be the set of attributes of each alternative according tothe determining variables established as follows: cost $\left( c _{1}\right)$, quality $\left( c _{2}\right)$, green products $\left( c_{3}\right)$ and environmental management $\left( c_{4}\right)$ of green products farming. Due of uncertainty, the information utilized by decision makers to make decisions is given as FHFR information. The attribute weight vector under consideration is $w=\left( 0.180,0.250,0.310,0.260\right) ^{T}$ and decision makers weights vector is $w=\left( 0.230,0.380,0.390\right) ^{T}.$ To assess the MCDM problem using the established framework for analyzing alternatives, the following computations are carried out as follows:

**[Step-1]**Analyses of the information provided by three experts using FHFRVs are demonstrated in Tables 2, 3, 4.

**Table 2:** Expert-1 information.

(a)		
	$c_{1}$	$c_{2}$
$A_{1}$	$$\left( \begin{array}{l} \left\{ \left( 0.10,0.20,0.50\right) ,\left( 0.30,0.40\right) \right\} , \\ \left\{ \left( 0.30,0.80,0.90\right) ,\left( 0.40,0.60\right) \right\} \end{array} \right)$$	$$\left( \begin{array}{l} \left\{ \left( 0.50,0.70,0.90\right) ,\left( 0.50,0.60,0.80\right) \right\} , \\ \left\{ \left( 0.30,0.50,0.60\right) ,\left( 0.70,0.90\right) \right\} \end{array} \right)$$
$A _{2}$	$$\left( \begin{array}{l} \left\{ \left( 0.50,0.60,0.70\right) ,\left( 0.70,0.90\right) \right\} , \\ \left\{ \left( 0.30,0.50,0.70\right) ,\left( 0.60,0.70\right) \right\} \end{array} \right)$$	$$\left( \begin{array}{l} \left\{ \left( 0.20,0.40,0.50\right) ,\left( 0.50\right) \right\} , \\ \left\{ \left( 0.60,0.70\right) ,\left( 0.30,0.50,0.90\right) \right\} \end{array} \right)$$
$A_{3}$	$$\left( \begin{array}{l} \left\{ \left( 0.40,0.50,0.60\right) ,\left( 0.60,0.70,0.80\right) \right\} ,\\ \left\{ \left( 0.70,0.80\right) ,\left( 0.10,0.40,0.70\right) \right\} \end{array} \right)$$	$$\left( \begin{array}{l} \left\{ \left( 0.10\right) ,\left( 0.50,0.60\right) \right\} , \\ \left\{ \left( 0.40,0.60,0.70\right) ,\left( 0.50,0.70\right) \right\} \end{array} \right)$$
$A _{4}$	$$\left( \begin{array}{l} \left\{ \left( 0.60,0.70,0.90\right) ,\left( 0.30,0.40,0.60\right) \right\} , \\ \left\{ \left( 0.20,0.70\right) ,\left( 0.70,0.80,0.90\right) \right\} \end{array} \right)$$	$$\left( \begin{array}{l} \left\{ \left( 0.30,0.40,0.50\right) ,\left( 0.40,0.70,0.90\right) \right\} , \\ \left\{ \left( 0.10,0.20\right) ,\left( 0.20,0.30\right) \right\} \end{array} \right)$$

(b)		
	$c_{3}$	$c_{4}$
$A_{1}$	$$\left( \begin{array}{l} \left\{ \left( 0.20,0.30,0.40\right) ,\left( 0.30,0.40,0.70\right) \right\} \textbf{,} \\ \left\{ \left( 0.10,0.50\right) ,\left( 0.30,0.50\right) \right\} \end{array} \right)$$	$$\left( \begin{array}{l} \left\{ \left( 0.50,0.60\right) \textbf{,}\left( 0.40,0.50,0.70\right) \right\} , \\ \left\{ \left( 0.60,0.80,0.90\right) ,\left( 0.60,0.70,0.90\right) \right\} \end{array} \right)$$
$A_{2}$	$$\left( \begin{array}{l} \left\{ \left( 0.40,0.50,0.80\right) ,\left( 0.40,0.50,0.70\right) \right\} , \\ \left\{ \left( 0.20,0.50\right) ,\left( 0.40,0.50\right) \right\} \end{array} \right)$$	$$\left( \begin{array}{l} \left\{ \left( 0.40,0.60,0.80\right) ,\left( 0.30,0.50\right) \right\} , \\ \left\{ \left( 0.70\right) ,\left( 0.10,0.30,0.40\right) \right\} \end{array} \right)$$
$A _{3}$	$$\left( \begin{array}{l} \left\{ \left( 0.30,0.60,0.70\right) ,\left( 0.50,0.70,0.80\right) \right\} , \\ \left\{ \left( 0.50,0.90\right) ,\left( 0.50,0.80\right) \right\} \end{array} \right)$$	$$\left( \begin{array}{l} \left\{ \left( 0.30,0.60\right) ,\left( 0.50,0.60,0.80\right) \right\} , \\ \left\{ \left( 0.10,0.30,0.70\right) ,\left( 0.30,0.40\right) \right\} \end{array} \right)$$
$A _{4}$	$$\left( \begin{array}{l} \left\{ \left( 0.30,0.40,0.50\right) ,\left( 0.70,0.80,0.90\right) \right\} , \\ \left\{ \left( 0.60,0.70\right) ,\left( 0.40,0.70\right) \right\} \end{array} \right)$$	$$\left( \begin{array}{l} \left\{ \left( 0.20,0.30,0.40\right) ,\left( 0.50,0.60,0.90\right) \right\} , \\ \left\{ \left( 0.30,0.40\right) ,\left( 0.70,0.80\right) \right\} \end{array} \right)$$

**Table 3:** Expert-2 information.

(a)		
	$c_{1}$	$c_{2}$
$A _{1}$	$$\left( \begin{array}{l} \left( \left\{ 0.20,0.30,0.40\right\} ,\left\{ 0.20,0.50\right\} \right) , \\ \left( \left\{ 0.40,0.60\right\} ,\left\{ 0.20,0.50\right\} \right) \end{array} \right)$$	$$\left( \begin{array}{l} \left( \left\{ 0.40,0.50,0.60\right\} ,\left\{ 0.30,0.70,0.80\right\} \right) , \\ \left( \left\{ 0.20,0.70,0.80\right\} ,\left\{ 0.20,0.80,0.90\right\} \right) \end{array} \right)$$
$A _{2}$	$$\left( \begin{array}{l} \left( \left\{ 0.10,0.30,0.40\right\} ,\left\{ 0.50,0.80\right\} \right) , \\ \left( \left\{ 0.50,0.60\right\} ,\left\{ 0.80,0.90\right\} \right) \end{array} \right)$$	$$\left( \begin{array}{l} \left( \left\{ 0.30,0.40,0.60\right\} ,\left\{ 0.70,0.80\right\} \right) , \\ \left( \left\{ 0.10,0.50\right\} ,\left\{ 0.30,0.70,0.80\right\} \right) \end{array} \right)$$
$A _{3}$	$$\left( \begin{array}{l} \left( \left\{ 0.60,0.70,0.80\right\} ,\left\{ 0.30,0.40\right\} \right) , \\ \left( \left\{ 0.30,0.80,0.90\right\} ,\left\{ 0.20,0.50,0.70\right\} \right) \end{array} \right)$$	$$\left( \begin{array}{l} \left( \left\{ 0.30,0.40,0.50\right\} ,\left\{ 0.40,0.70,0.90\right\} \right) , \\ \left( \left\{ 0.40,0.60,0.70\right\} ,\left\{ 0.70,0.80,0.90\right\} \right) \end{array} \right)$$
$A _{4}$	$$\left( \begin{array}{l} \left( \left\{ 0.10,0.20,0.30\right\} ,\left\{ 0.50,0.70\right\} \right) , \\ \left( \left\{ 0.20,0.40\right\} ,\left\{ 0.70,0.80\right\} \right) \end{array} \right)$$	$$\left( \begin{array}{l} \left( \left\{ 0.50,0.70,0.80\right\} ,\left\{ 0.30,0.50,0.70\right\} \right) , \\ \left( \left\{ 0.30,0.40,0.60\right\} ,\left\{ 0.40,0.50,0.70\right\} \right) \end{array} \right)$$

(b)		
	$c_{3}$	$c_{4}$
$A _{1}$	$$\left( \begin{array}{l} \left( \left\{ 0.20,0.40\right\} ,\left\{ 0.30,0.50\right\} \right) \textbf{,}\\ \left( \left\{ 0.40,0.70,0.80\right\} ,\left\{ 0.20,0.60\right\} \right) \end{array} \right)$$	$$\left( \begin{array}{l} \left( \left\{ 0.10,0.20\right\} \textbf{,}\left\{ 0.40,0.60\right\} \right) ,\\ \left( \left\{ 0.20,0.50\right\} ,\left\{ 0.70,0.90\right\} \right) \end{array} \right)$$
$A _{2}$	$$\left( \begin{array}{l} \left( \left\{ 0.30,0.50,0.70\right\} ,\left\{ 0.20,0.60\right\} \right) , \\ \left( \left\{ 0.60,0.70,0.80\right\} ,\left\{ 0.20,0.80\right\} \right) \end{array} \right)$$	$$\left( \begin{array}{l} \left( \left\{ 0.20,0.30\right\} ,\left\{ 0.40,0.60,0.70\right\} \right) , \\ \left( \left\{ 0.10,0.30,0.50\right\} ,\left\{ 0.20,0.30,0.50\right\} \right) \end{array} \right)$$
$A _{3}$	$$\left( \begin{array}{l} \left( \left\{ 0.50,0.60,0.70\right\} ,\left\{ 0.30,0.50\right\} \right) , \\ \left( \left\{ 0.70,0.80,0.90\right\} ,\left\{ 0.20,0.30,0.50\right\} \right) \end{array} \right)$$	$$\left( \begin{array}{l} \left( \left\{ 0.20,0.70,0.80\right\} ,\left\{ 0.20,0.70\right\} \right) , \\ \left( \left\{ 0.10,0.20\right\} ,\left\{ 0.50,0.60,0.70\right\} \right) \end{array} \right)$$
$A _{4}$	$$\left( \begin{array}{l} \left( \left\{ 0.60,0.70,0.90\right\} ,\left\{ 0.20,0.50\right\} \right) , \\ \left( \left\{ 0.60,0.90\right\} ,\left\{ 0.20,0.50\right\} \right) \end{array} \right)$$	$$\left( \begin{array}{l} \left( \left\{ 0.30,0.50\right\} ,\left\{ 0.40,0.60,0.70\right\} \right) , \\ \left( \left\{ 0.20,0.30,0.60\right\} ,\left\{ 0.40,0.50,0.70\right\} \right) \end{array} \right)$$

**Table 4:** Expert-3 information.

(a)		
	$c_{1}$	$c_{2}$
$A _{1}$	$$\left( \begin{array}{l} \left( \left\{ 0.40,0.70,0.90\right\} ,\left\{ 0.30,0.60,0.80\right\} \right) , \\ \left( \left\{ 0.20,0.30,0.80\right\} ,\left\{ 0.70,0.80,0.90\right\} \right) \end{array} \right)$$	$$\left( \begin{array}{l} \left( \left\{ 0.40,0.70,0.80\right\} ,\left\{ 0.70,0.80\right\} \right) , \\ \left( \left\{ 0.30,0.50,0.60\right\} ,\left\{ 0.70,0.80\right\} \right) \end{array} \right)$$
$A _{2}$	$$\left( \begin{array}{l} \left( \left\{ 0.10,0.30,0.40\right\} ,\left\{ 0.50,0.60,0.90\right\} \right) , \\ \left( \left\{ 0.20,0.30,0.70\right\} ,\left\{ 0.20,0.60,0.80\right\} \right) \end{array} \right)$$	$$\left( \begin{array}{l} \left( \left\{ 0.20,0.30,0.70\right\} ,\left\{ 0.30,0.80,0.90\right\} \right) , \\ \left( \left\{ 0.10,0.50,0.80\right\} ,\left\{ 0.20,0.70,0.80\right\} \right) \end{array} \right)$$
$A _{3}$	$$\left( \begin{array}{l} \left( \left\{ 0.20,0.30,0.50\right\} ,\left\{ 0.40,0.80,0.90\right\} \right) , \\ \left( \left\{ 0.10,0.80,0.90\right\} ,\left\{ 0.40,0.70\right\} \right) \end{array} \right)$$	$$\left( \begin{array}{l} \left( \left\{ 0.20,0.30,0.80\right\} ,\left\{ 0.20,0.80\right\} \right) , \\ \left( \left\{ 0.50,0.80,0.90\right\} ,\left\{ 0.20,0.90\right\} \right) \end{array} \right)$$
$A _{4}$	$$\left( \begin{array}{l} \left( \left\{ 0.10,0.50,0.70\right\} ,\left\{ 0.50,0.80\right\} \right) , \\ \left( \left\{ 0.30,0.50,0.70\right\} ,\left\{ 0.40,0.90\right\} \right) \end{array} \right)$$	$$\left( \begin{array}{l} \left( \left\{ 0.20,0.30\right\} ,\left\{ 0.50,0.60\right\} \right) , \\ \left( \left\{ 0.30,0.80,0.90\right\} ,\left\{ 0.70,0.80,0.90\right\} \right) \end{array} \right)$$
**(b)**		
	$c_{3}$	$c_{4}$
$A _{1}$	$$\left( \begin{array}{l} \left( \left\{ 0.20,0.30,0.80\right\} ,\left\{ 0.50,0.60,0.70\right\} \right) , \\ \left( \left\{ 0.30,0.50,0.60\right\} ,\left\{ 0.20,0.80,0.90\right\} \right) \end{array} \right)$$	$$\left( \begin{array}{l} \left( \left\{ 0.20,0.30,0.70\right\} ,\left\{ 0.20,0.30,0.70\right\} \right) , \\ \left( \left\{ 0.20,0.30,0.80\right\} ,\left\{ 0.50,0.70\right\} \right) \end{array} \right)$$
$A _{2}$	$$\left( \begin{array}{l} \left( \left\{ 0.10,0.30,0.60\right\} ,\left\{ 0.40,0.60,0.80\right\} \right) , \\ \left( \left\{ 0.60,0.70,0.90\right\} ,\left\{ 0.30,0.80,0.90\right\} \right) \end{array} \right)$$	$$\left( \begin{array}{l} \left( \left\{ 0.10,0.20,0.30\right\} ,\left\{ 0.20,0.50\right\} \right) , \\ \left( \left\{ 0.30,0.40,0.60\right\} ,\left\{ 0.10,0.20\right\} \right) \end{array} \right)$$
$A _{3}$	$$\left( \begin{array}{l} \left( \left\{ 0.10,0.20,0.30\right\} ,\left\{ 0.30,0.50,0.90\right\} \right) , \\ \left( \left\{ 0.20,0.30,0.40\right\} ,\left\{ 0.20,0.40,0.60\right\} \right) \end{array} \right)$$	$$\left( \begin{array}{l} \left( \left\{ 0.20,0.80,0.90\right\} ,\left\{ 0.10,0.20\right\} \right) , \\ \left( \left\{ 0.20,0.40,0.50\right\} ,\left\{ 0.70,0.80\right\} \right) \end{array} \right)$$
$A _{4}$	$$\left( \begin{array}{l} \left( \left\{ 0.20,0.30,0.70\right\} ,\left\{ 0.80,0.90\right\} \right) , \\ \left( \left\{ 0.20,0.30,0.80\right\} ,\left\{ 0.10,0.20,0.30\right\} \right) \end{array} \right)$$	$$\left( \begin{array}{l} \left( \left\{ 0.20,0.30,0.80\right\} ,\left\{ 0.20,0.30\right\} \right) , \\ \left( \left\{ 0.30,0.50,0.80\right\} ,\left\{ 0.40,0.50\right\} \right) \end{array} \right)$$

**[Step-2]** All of the expert information is of the benefit type. In this situation, it is not necessary to normalize the FHFRVs.

**[Step-3]** Table 5 assesses the collective information of three expert analysts using the FHFRWA aggregation operator.

**Table 5:** Collective aggregation of FHFR information.

(a)		
	$c_{1}$	$c_{2}$
$A _{1}$	$$\left( \begin{array}{l} \left( \begin{array}{l} \left\{ 0.240,0.544,0.755\right\} , \\ \left\{ 0.257,0.510,0.840\right\} \end{array} \right) , \\ \left( \begin{array}{l} \left\{ 0.323,0.617,0.760\right\} , \\ \left\{ 0.274,0.626,0.959\right\} \end{array} \right) \end{array} \right)$$	$$\left( \begin{array}{l} \left( \begin{array}{l} \left\{ 0.428,0.644,0.663\right\} , \\ \left\{ 0.469,0.711,0.950\right\} \end{array} \right) , \\ \left( \begin{array}{l} \left\{ 0.270,0.599,0.728\right\} , \\ \left\{ 0.382,0.775,0.937\right\} \end{array} \right) \end{array} \right)$$
$A _{2}$	$$\left( \begin{array}{l} \left( \begin{array}{l} \left\{ 0.314,0.420,0.367\right\} , \\ \left\{ 0.521,0.693,0.921\right\} \end{array} \right) , \\ \left( \begin{array}{l} \left\{ 0.388,0.500,0.612\right\} , \\ \left\{ 0.436,0.811,0.959\right\} \end{array} \right) \end{array} \right)$$	$$\left( \begin{array}{l} \left( \begin{array}{l} \left\{ 0.248,0.367,0.629\right\} , \\ \left\{ 0.465,0.842,0.959\right\} \end{array} \right) , \\ \left( \begin{array}{l} \left\{ 0.380,0.460,0.624\right\} , \\ \left\{ 0.256,0.647,0.822\right\} \end{array} \right) \end{array} \right)$$
$A _{3}$	$$\left( \begin{array}{l} \left( \begin{array}{l} \left\{ 0.471,0.566,0.681\right\} , \\ \left\{ 0.393,0.596,0.921\right\} \end{array} \right) , \\ \left( \begin{array}{l} \left\{ 0.467,0.751,0.919\right\} , \\ \left\{ 0.223,0.541,0.804\right\} \end{array} \right) \end{array} \right)$$	$$\left( \begin{array}{l} \left( \begin{array}{l} \left\{ 0.239,0.327,0.655\right\} , \\ \left\{ 0.321,0.800,0.960\right\} \end{array} \right) , \\ \left( \begin{array}{l} \left\{ 0.445,0.703,0.808\right\} , \\ \left\{ 0.397,0.812,0.960\right\} \end{array} \right) \end{array} \right)$$
$A _{4}$	$$\left( \begin{array}{l} \left( \begin{array}{l} \left\{ 0.380,0.520,0.672\right\} , \\ \left\{ 0.444,0.648,0.889\right\} \end{array} \right) , \\ \left( \begin{array}{l} \left\{ 0.249,0.420,0.532\right\} , \\ \left\{ 0.562,0.837,0.976\right\} \end{array} \right) \end{array} \right)$$	$$\left( \begin{array}{l} \left( \begin{array}{l} \left\{ 0.388,0.553,0.639\right\} , \\ \left\{ 0.391,0.580,0.852\right\} \end{array} \right) , \\ \left( \begin{array}{l} \left\{ 0.276,0.641,0.763\right\} , \\ \left\{ 0.424,0.534,0.838\right\} \end{array} \right) \end{array} \right)$$
**(b)**		
	$c_{3}$	$c_{4}$
$A _{1}$	$$\left( \begin{array}{l} \left( \begin{array}{l} \left\{ 0.200,0.345,0.634\right\} , \\ \left\{ 0.366,0.510,0.801\right\} \end{array} \right) \textbf{,} \\ \left( \begin{array}{l} \left\{ 0.328,0.599,0.673\right\} , \\ \left\{ 0.219,0.643,0.959\right\} \end{array} \right) \end{array} \right)$$	$$\left( \begin{array}{l} \left( \begin{array}{l} \left\{ 0.322,0.406,0.578\right\} \textbf{,} \\ \left\{ 0.232,0.535,0.921\right\} \end{array} \right) , \\ \left( \begin{array}{l} \left\{ 0.392,0.587,0.760\right\} , \\ \left\{ 0.592,0.770,0.976\right\} \end{array} \right) \end{array} \right)$$
$A _{2}$	$$\left( \begin{array}{l} \left( \begin{array}{l} \left\{ 0.295,0.444,0.699\right\} , \\ \left\{ 0.307,0.575,0.844\right\} \end{array} \right) , \\ \left( \begin{array}{l} \left\{ 0.610,0.721,0.815\right\} , \\ \left\{ 0.274,0.718,0.959\right\} \end{array} \right) \end{array} \right)$$	$$\left( \begin{array}{l} \left( \begin{array}{l} \left\{ 0.264,0.406,0.544\right\} , \\ \left\{ 0.285,0.535,0.873\right\} \end{array} \right) , \\ \left( \begin{array}{l} \left\{ 0.467,0.328,0.513\right\} , \\ \left\{ 0.130,0.256,0.622\right\} \end{array} \right) \end{array} \right)$$
$A _{3}$	$$\left( \begin{array}{l} \left( \begin{array}{l} \left\{ 0.388,0.527,0.700\right\} , \\ \left\{ 0.494,0.679,0.950\right\} \end{array} \right) , \\ \left( \begin{array}{l} \left\{ 0.560,0.761,0.814\right\} , \\ \left\{ 0.188,0.320,0.480\right\} \end{array} \right) \end{array} \right)$$	$$\left( \begin{array}{l} \left( \begin{array}{l} \left\{ 0.231,0.731,0.815\right\} , \\ \left\{ 0.188,0.414,0.950\right\} \end{array} \right) , \\ \left( \begin{array}{l} \left\{ 0.155,0.325,0.517\right\} , \\ \left\{ 0.506,0.611,0.873\right\} \end{array} \right) \end{array} \right)$$
$A _{4}$	$$\left( \begin{array}{l} \left( \begin{array}{l} \left\{ 0.459,0.553,0.793\right\} , \\ \left\{ 0.458,0.700,0.976\right\} \end{array} \right) , \\ \left( \begin{array}{l} \left\{ 0.406,0.565,0.814\right\} , \\ \left\{ 0.179,0.377,0.625\right\} \end{array} \right) \end{array} \right)$$	$$\left( \begin{array}{l} \left( \begin{array}{l} \left\{ 0.248,0.403,0.634\right\} , \\ \left\{ 0.321,0.457,0.852\right\} \end{array} \right) , \\ \left( \begin{array}{l} \left\{ 0.270,0.421,0.677\right\} , \\ \left\{ 0.454,0.557,0.873\right\} \end{array} \right) \end{array} \right)$$

**[Step-4]** In order to use the suggested aggregation operators, the aggregate information of the alternative under the specified set of attributes is evaluated.

Case-1: The aggregation information utilizing FHFRWA operator is displayed in Table 6:

**Table 6:** Aggregated information using *q*-ROHFRWA.

$A _{1}$	$$\left( \begin{array}{l} \left( \left\{ 0.710,0.811,0.893\right\} ,\left\{ 0.302,0.544,0.796\right\} \right) \textbf{,} \\ \left( \left\{ 0.739,0.877,0.940\right\} ,\left\{ 0.330,0.694,0.886\right\} \right) \end{array} \right)$$
$A _{2}$	$$\left( \begin{array}{l} \left( \left\{ 0.670,0.773,0.872\right\} ,\left\{ 0.345,0.605,0.819\right\} \right) \textbf{,} \\ \left( \left\{ 0.820,0.827,0.894\right\} ,\left\{ 0.218,0.511,0.781\right\} \right) \end{array} \right)$$
$A _{3}$	$$\left( \begin{array}{l} \left( \left\{ 0.703,0.907,0.950\right\} ,\left\{ 0.305,0.581,0.873\right\} \right) \textbf{,} \\ \left( \left\{ 0.759,0.880,0.935\right\} ,\left\{ 0.292,0.513,0.677\right\} \right) \end{array} \right)$$
$A _{4}$	$$\left( \begin{array}{l} \left( \left\{ 0.726,0.814,0.915\right\} ,\left\{ 0.377,0.567,0.839\right\} \right) \textbf{,} \\ \left( \left\{ 0.695,0.827,0.929\right\} ,\left\{ 0.334,0.510,0.768\right\} \right) \end{array} \right)$$

Case-2: Information aggregated employing the FHFROWA operator is displayed in Table 7:

**Table 7:** Information aggregated utilizing FHFROWA.

$A _{1}$	$$\left( \begin{array}{l} \left( \left\{ 0.716,0.818,0.893\right\} ,\left\{ 0.306,0.555,0.879\right\} \right) \textbf{,} \\ \left( \left\{ 0.738,0.877,0.941\right\} ,\left\{ 0.342,0.701,0.955\right\} \right) \end{array}\right)$$
$A _{2}$	$$\left( \begin{array}{l} \left( \left\{ 0.688,0.774,0.837\right\} ,\left\{ 0.381,0.655,0.902\right\} \right) \textbf{,} \\ \left( \left\{ 0.790,0.837,0.898\right\} ,\left\{ 0.241,0.549,0.819\right\} \right) \end{array}\right)$$
$A _{3}$	$$\left( \begin{array}{l} \left( \left\{ 0.705,0.840,0.925\right\} ,\left\{ 0.292,0.582,0.943\right\} \right) \textbf{,} \\ \left( \left\{ 0.792,0.919,0.963\right\} ,\left\{ 0.309,0.562,0.786\right\} \right) \end{array}\right)$$
$A _{4}$	$$\left( \begin{array}{l} \left( \left\{ 0.749,0.835,0.915\right\} ,\left\{ 0.375,0.561,0.877\right\} \right) \textbf{,} \\ \left( \left\{ 0.677,0.820,0.904\right\} ,\left\{ 0.388,0.566,0.836\right\} \right) \end{array} \right)$$

Case-3: Aggregation information using FHFRHWA operator presented in Table 8 with associated weights vector that is $\left( 0.180,0.230,0.280,0.310\right) ^{T}.$

**Table 8:** Information aggregated employing FHFRHWA.

$A _{1}$	$$\left( \begin{array}{l} \left( \left\{ 0.722,0.853,0.925\right\} ,\left\{ 0.316,0.557,0.874\right\} \right) \textbf{,} \\ \left( \left\{ 0.741,0.889,0.940\right\} ,\left\{ 0.333,0.698,0.956\right\} \right) \end{array} \right)$$
$A _{2}$	$$\left( \begin{array}{l} \left( \left\{ 0.714,0.794,0.865\right\} ,\left\{ 0.358,0.598,0.791\right\} \right) \textbf{,} \\ \left( \left\{ 0.821,0.861,0.911\right\} ,\left\{ 0.235,0.542,0.820\right\} \right) \end{array} \right)$$
$A _{3}$	$$\left( \begin{array}{l} \left( \left\{ 0.737,0.863,0.936\right\} ,\left\{ 0.296,0.580,0.945\right\} \right) \textbf{,} \\ \left( \left\{ 0.820,0.929,0.966\right\} ,\left\{ 0.314,0.549,0.768\right\} \right) \end{array} \right)$$
$A _{4}$	$$\left( \begin{array}{l} \left( \left\{ 0.772,0.847,0.920\right\} ,\left\{ 0.376,0.561,0.880\right\} \right) \textbf{,} \\ \left( \left\{ 0.714,0.842,0.918\right\} ,\left\{ 0.367,0.536,0.815\right\} \right) \end{array} \right)$$

**Step-5 & 6]** The score values for all alternatives determined by the specified aggregation operators are summarized in Table 9.

**Table 9:** Ranking of alternative.

Proposed operators	Score values of alternatives				Ranking
	$A _{1}$	$A _{2}$	$A _{3}$	$A _{4}$	
FHFRWA	0.618	0.631	0.657	0.625	$A _{3}>A _{2}> A _{4}>A _{1}$
FHFROWA	0.6037	0.606	0.639	0.608	$A _{3}>A _{4}>A _{2}>A _{1}$
FHFRHWA	0.611	0.635	0.650	0.623	$A _{3}>A _{2}>A _{4}>A _{1}$

**Figure 2:**
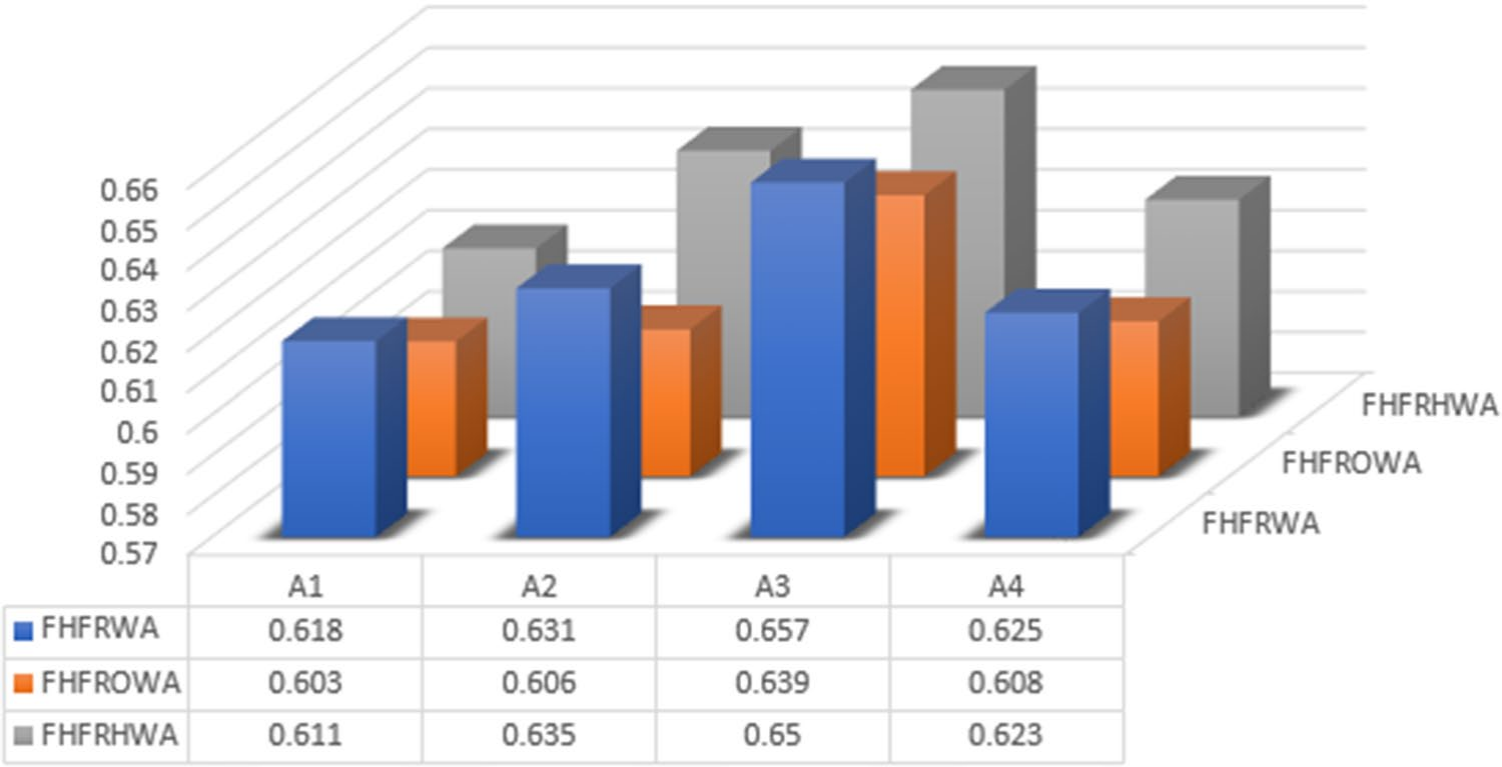
The pictorial diagram of the ranking of underdeveloped operators.

Graphical representations of rankings for each alternative are illustrated in Fig. 2.

## The comparative evaluation

In this section, we intend to enhanced the VIKOR scheme for the FHFR information in order to deal with MCGDM problems.

### The improved FHFR-VIKOR technique

The following is a detailed explanation of the modified form of VIKOR approach based on FHFR information:

Step-1: Construct evaluation matrices for the experts in the form of FHFRVs.
Step-2: Through using FHFRWA aggregation operator, compute the collected information of decision makers along their weights vector and obtained the aggregated decision matrix.
Step-3: Compute the positive ideal solutions (PIS) ${\mathcal {T}}^+$ and negative ideal solutions (NIS) ${\mathcal {T}}^-$ in the form of FHFR information as follows:
  $$\begin{aligned} {\mathcal {T}}^{+}= & {} \left( \mathcal {Y} _{1}^{+},\mathcal {Y} _{2}^{+},\mathcal {Y} _{3}^{+},...,\mathcal {Y} _{\ell }^{+}\right) =\left( \max _{\imath }\mathcal {Y} _{\imath 1},\max _{\imath }\mathcal {Y} _{\imath 2},\max _{\imath }\mathcal {Y} _{\imath 3},...,\max _{\imath }\mathcal {Y} _{\imath n}.\right) , \\ {\mathcal {T}}^{-}= & {} \left( \mathcal {Y} _{1}^{-},\mathcal {Y} _{2}^{-},\mathcal {Y} _{3}^{-},...,\mathcal {Y} _{\ell }^{-}\right) =\left( \min _{\imath }\mathcal {Y} _{\imath 1},\min _{\imath }\mathcal {Y} _{\imath 2},\min _{\imath }\mathcal {Y} _{\imath 3},...,\min _{\imath }\mathcal {Y} _{\imath n}.\right) \end{aligned}$$
Step-4: To determine the FHFR group utility measure $S_{\imath }(\imath =1,2,3,...,\ell )$ and the regret measure $R_{\imath }(\imath =1,2,3,...,\ell )$ of all alternatives $L=\left( A _{1},A_{2},A _{3},...,A_{\ell }\right)$ applying the formulas mentioned below:
  $$\begin{aligned} S_{\imath }= & {} \bigoplus _{j=1}^{\ell }\frac{\propto _{j}d\left( \mathcal {Y} _{\imath j},\mathcal {Y} _{j}^{+}\right) }{d\left( \mathcal {Y} _{j}^{+},\mathcal {Y} _{j}^{-}\right) },\text { } \imath =1,2,3,4,...,m. \\ R_{\imath }= & {} \max \frac{\propto _{j}d\left( \mathcal {Y} _{\imath j},\mathcal {Y} _{j}^{+}\right) }{ d\left( \mathcal {Y} _{j}^{+},\mathcal {Y} _{j}^{-}\right) },\text { }\imath =1,2,3,4,...,m \end{aligned}$$
Step-5: Determine the maximum and minimum values of *S* and *R*, respectively as follows:
  $$\begin{aligned} S^{\diamondsuit }=\min _{\imath }S_{\imath },\text { }S^{\circ }=\max _{\imath }S_{\imath },\text { } R^{\diamondsuit }=\min _{\imath }R_{\imath },\text { }R^{\circ }=\max _{\imath }R_{\imath },\text { }\imath =1,2,3,...,\ell . \end{aligned}$$
  Lastly, we integrate the features of both the group utility $S_{\imath }$ and the individual regret $R_{\imath }$ in order to assess the ranking measure $Q_{\imath }$ for the alternative $L=\left( A _{1},A _{2},A _{3},...,A _{\ell }\right)$ as follows:
  $$\begin{aligned} Q_{\imath }=\chi \frac{S_{\imath }-S^{\diamondsuit }}{S^{\circ }-S^{\diamondsuit }}+\left( 1-\chi \right) \frac{ R_{\imath }-R^{\diamondsuit }}{R^{\circ }-R^{\diamondsuit }},R_{\imath },\text { }\imath =1,2,3,...,\ell , \end{aligned}$$
  where $\chi$ is the strategic weight of the majority of parameters (the parameter with the largest group utility) and is essential for assessing the compromised solution. The value chosen from the range [0, 1],  however 0.5 is a common number, we utilized it.
Step-6: Furthermore, the alternatives are ordered in decreasing order for the group utility measure $S_i$, individual regret measure $R_\imath$, and ranking measure $Q_\imath$. Here, we obtained three ranking lists that will help us determine the best compromise alternative.

**Table 10:** The PIS and NIS based on FHFR information.

Criteria	${\mathcal {T}}^{+}$	${\mathcal {T}}^{-}$
$c_{1}$	$$\left( \begin{array}{l} \left( \begin{array}{l} \left\{ 0.380,0.520,0.672\right\} , \\ \left\{ 0.444,0.648,0.889\right\} \end{array} \right) , \\ \left( \begin{array}{l} \left\{ 0.249,0.420,0.532\right\} , \\ \left\{ 0.562,0.837,0.976\right\} \end{array} \right) \end{array} \right)$$	$$\left( \begin{array}{l} \left( \begin{array}{l} \left\{ 0.314,0.420,0.367\right\} , \\ \left\{ 0.521,0.693,0.921\right\} \end{array} \right) , \\ \left( \begin{array}{l} \left\{ 0.388,0.500,0.612\right\} , \\ \left\{ 0.436,0.811,0.959\right\} \end{array} \right) \end{array} \right)$$
$c_{2}$	$$\left( \begin{array}{l} \left( \begin{array}{l} \left\{ 0.248,0.367,0.629\right\} , \\ \left\{ 0.465,0.842,0.959\right\} \end{array} \right) , \\ \left( \begin{array}{l} \left\{ 0.380,0.460,0.624\right\} , \\ \left\{ 0.256,0.647,0.822\right\} \end{array} \right) \end{array} \right)$$	$$\left( \begin{array}{l} \left( \begin{array}{l} \left\{ 0.239,0.327,0.655\right\} , \\ \left\{ 0.321,0.800,0.960\right\} \end{array} \right) , \\ \left( \begin{array}{l} \left\{ 0.445,0.703,0.808\right\} , \\ \left\{ 0.397,0.812,0.960\right\} \end{array} \right) \end{array} \right)$$
$c_{3}$	$$\left( \begin{array}{l} \left( \begin{array}{l} \left\{ 0.459,0.553,0.793\right\} , \\ \left\{ 0.458,0.700,0.976\right\} \end{array} \right) , \\ \left( \begin{array}{l} \left\{ 0.406,0.565,0.814\right\} , \\ \left\{ 0.179,0.377,0.625\right\} \end{array} \right) \end{array} \right)$$	$$\left( \begin{array}{l} \left( \begin{array}{l} \left\{ 0.295,0.444,0.699\right\} , \\ \left\{ 0.307,0.575,0.844\right\} \end{array} \right) , \\ \left( \begin{array}{l} \left\{ 0.610,0.721,0.815\right\} , \\ \left\{ 0.274,0.718,0.959\right\} \end{array} \right) \end{array} \right)$$
$c_{4}$	$$\left( \begin{array}{l} \left( \begin{array}{l} \left\{ 0.264,0.406,0.544\right\} , \\ \left\{ 0.285,0.535,0.873\right\} \end{array} \right) , \\ \left( \begin{array}{l} \left\{ 0.467,0.328,0.513\right\} , \\ \left\{ 0.130,0.256,0.622\right\} \end{array} \right) \end{array} \right)$$	$$\left( \begin{array}{l} \left( \begin{array}{l} \left\{ 0.322,0.406,0.578\right\} \textbf{,} \\ \left\{ 0.232,0.535,0.921\right\} \end{array} \right) , \\ \left( \begin{array}{l} \left\{ 0.392,0.587,0.760\right\} , \\ \left\{ 0.592,0.770,0.976\right\} \end{array} \right) \end{array} \right)$$

**Table 11:** $$S_{\imath },\text { }R_{\imath },\text { }Q_{\imath }\text { for each alternative}$$.

Alternatives	$S_{\imath }$	$R_{\imath }$	$Q_{\imath }$
$A _{1}$	1.0520	0.3599	0.9918
$A _{2}$	0.4900	0.3099	0.5000
$A _{3}$	1.3689	0.4050	1.2319
$A _{4}$	0.7422	0.3421	0.6588

### Numerical example of the improved FHFR-VIKOR methodology

In this section, we implement an improved FHFR-VIKOR approach to the MAGDM problem in order to identify the best green supplier in process industries through using four criteria mentioned in the following numerical example.

**Figure 3:**
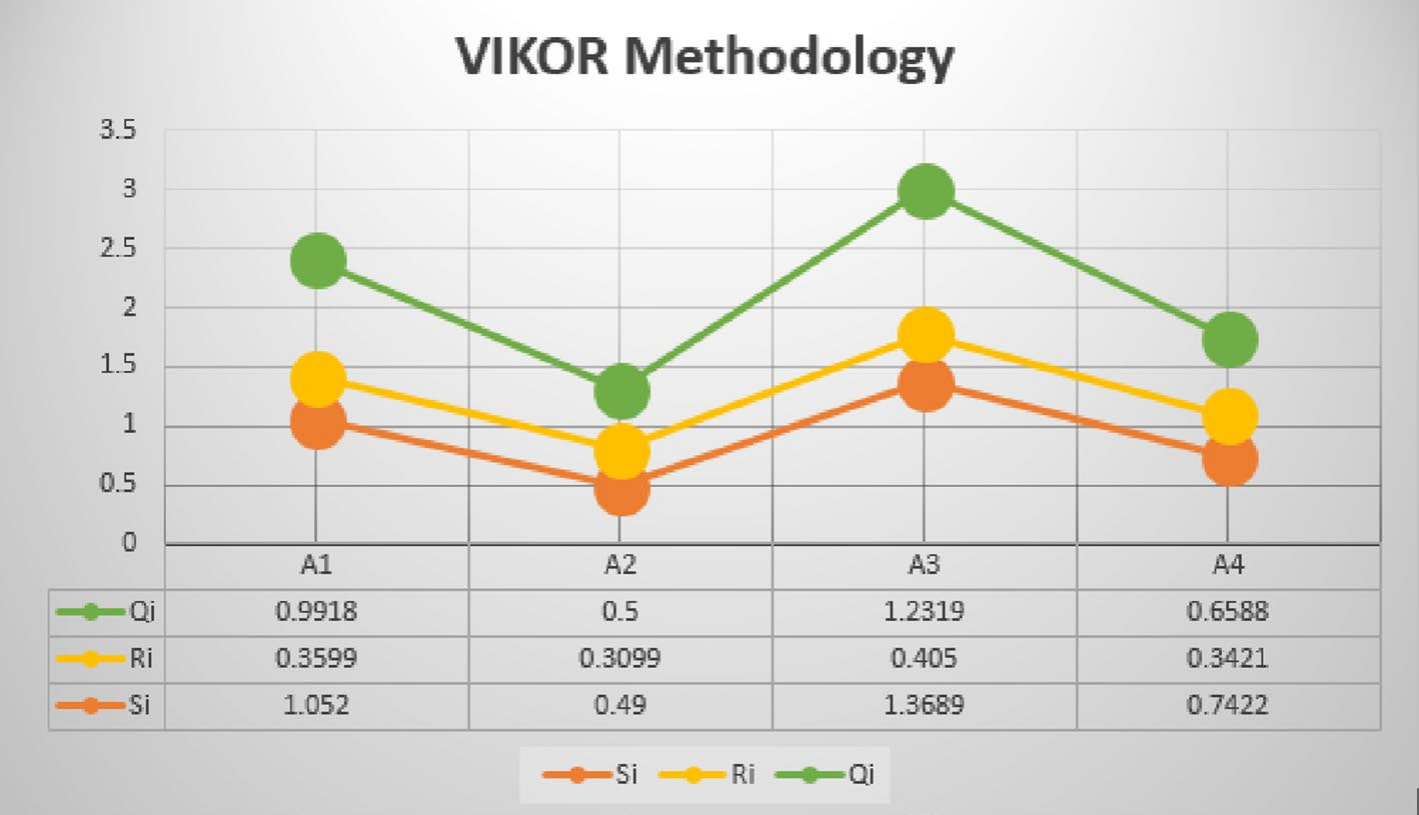
The graphical depiction of ranking according to the modified VIKOR-method.

Step-1: The information based on FHFRVs of the three professional experts is analyzed in Tables 2, 3, 4.
Step-2: The aggregated information of the team of experts, as determined by the FHFRWA aggregation operator, is summarized in Table 5.
Step-3: The FHFR PIS (${\mathcal {T}}^{+}$) and the FHFR NIS (${\mathcal {T}}^{-}$) are computed in Table 10:
Step-4: The FHFR group utility measure $S_{\imath }(\imath =1,2,3,4)$ and the regret measure $R_{\imath }(\imath =1,2,3,4)$ of the alternatives under consideration are summarized in Table 11.
Step-5 & 6: Alternative rankings based on the group utility measure $S_{\imath }$, the individual regret measure $R_{\imath }$, and the ranking measure $Q_{\imath }$ are indicated in Table 12. Figure 3 depicts a graphical illustration of the ranking according to the modified VIKOR approach.

**Table 12:** Alternative ranking depending on $S_{\imath },$ $R_{\imath },$ and $Q_{\imath }$.

Alternatives	Ranking order of $S_{\imath }$	Ranking order of $R_{\imath }$	Ranking order of $Q_{\imath }$
$A _{1}$	2	2	2
$A _{2}$	4	4	4
$A _{3}$	1	1	1
$A _{4}$	3	3	3

### The improved TOPSIS approach based on FHFR information

Hwang and Yoon^76^ invented the TOPSIS technique for optimum solution, allowing decision makers to examine the PIS and NIS. TOPSIS is based on the idea that the best alternative is the one that is nearer to the positive ideal while being the far away from the negative ideal solution^77,78^. The supplier selection in the process industries through using four criteria mentioned in the following numerical example. The following are the major components of aforesaid scheme:

Step-1: The information provided by three professionals experts is analyzed employing FHFRVs in Tables 2 through 4.
Step-2: The collective information of professional experts utilizing the FHFRWA AOPs is given in Table 5.
Step-3: The FHFR PIS ${\mathcal {T}}^{+}$ and the FHFR NIS ${\mathcal {T}}^{-}$ on the basis of their score values are computed in Table 10:
Step-5: Both the PIS and the NIS are determined using the score value. In this context, the PIS and NIS are referred to as ${\mathcal {T}} ^{+}=\left( \mathcal {Y} _{1}^{+},\mathcal {Y} _{2}^{+},\mathcal {Y} _{3}^{+},...,\mathcal {Y} _{\ell }^{+}\right)$ and ${\mathcal {T}}^{-}=\left( \mathcal {Y} _{1}^{-},\mathcal {Y} _{2}^{-},\mathcal {Y} _{3}^{-},...,\mathcal {Y} _{\ell }^{-}\right)$ respectively. For PIS ${\mathcal {T}}^{+}$, it can be determined by employing the formula as follows:
  $$\begin{aligned} {\mathcal {T}}^{+}= & {} \left( \mathcal {Y} _{1}^{+},\mathcal {Y} _{2}^{+},\mathcal {Y} _{3}^{+},...,\mathcal {Y} _{\ell }^{+}\right) \\= & {} \left( \max _{\imath }score(\mathcal {Y} _{\imath 1}),\max _{\imath }score\mathcal {Y} _{\imath 2},\max _{\imath }score\mathcal {Y} _{\imath 3},...,\max _{\imath }score\mathcal {Y} _{\imath n}.\right) \end{aligned}$$
  In a similar fashion, the NIS can be obtained using the formula as follows:
  $$\begin{aligned} {\mathcal {T}}^{-}= & {} \left( \mathcal {Y} _{1}^{-},\mathcal {Y} _{2}^{-},\mathcal {Y} _{3}^{-},...\mathcal {Y} _{\ell }^{-}\right) \\= & {} \left( \min _{\imath }score\mathcal {Y} _{\imath 1},\min _{\imath }score\mathcal {Y} _{\imath 2},\min _{\imath }score\mathcal {Y} _{\imath 3},...,\min _{\imath }score\mathcal {Y} _{\imath n}.\right) \end{aligned}$$
  After that, determine the geometric distance between each of the alternatives and the PIS ${\mathcal {T}}^{+}$ using the formula as follows:
  $$\begin{aligned} d(\alpha _{\imath j},{\mathcal {T}}^{+})= & {} \frac{1}{8}\left( \begin{array}{l} \left( \begin{array}{l} \frac{1}{\sharp h}\sum _{s=1}^{\sharp h}\left| \left( \underline{\mu } _{\imath j(s)}\right) ^{2}-\left( \underline{\mu }_{\imath }^{+}\right) ^{2}\right| \\ +\left| \left( \overline{\mu }_{\imath j(s)}\right) ^{2}-\left( \overline{\mu } _{\imath (s)}^{+}\right) ^{2}\right| \end{array}\right) \\ +\left( \begin{array}{l} \frac{1}{\sharp g}\sum _{s=1}^{\sharp g}\left| \left( \underline{{\mathcal {V}} } _{\imath j(s)}\right) ^{2}-\left( \underline{{\mathcal {V}} }_{\imath (s)}^{+}\right) ^{2}\right| \\ +\left| \left( \overline{{\mathcal {V}} _{h}}_{_{\imath j}}\right) ^{2}-\left( \overline{ {\mathcal {V}} _{h}}_{_{\imath }}^{+}\right) ^{2}\right| \end{array} \right) \end{array} \right) ,\text { } \\ \text {where }\imath= & {} 1,2,3,...,\ell ,\text {and }j=1,2,3,...,m. \end{aligned}$$
  In a similar manner, the geometric distance between each of the alternatives and NIS ${\mathcal {T}}^{-}$ may be expressed as follows:
  $$\begin{aligned} d(\alpha _{\imath j},{\mathcal {T}}^{-})= & {} \frac{1}{8}\left( \begin{array}{l} \left( \begin{array}{l} \frac{1}{\sharp h}\sum _{s=1}^{\sharp h}\left| \left( \underline{\mu } _{\imath j(s)}\right) ^{2}-\left( \underline{\mu }_{\imath (s)}^{-}\right) ^{2}\right| \\ +\left| \left( \overline{\mu }_{\imath j(s)}\right) ^{2}-\left( \overline{\mu } _{\imath (s)}^{-}\right) ^{2}\right| \end{array} \right) \\ +\left( \begin{array}{l} \frac{1}{\sharp g}\sum _{s=1}^{\sharp g}\left| \left( \underline{{\mathcal {V}} } _{\imath j(s)}\right) ^{2}-\left( \underline{{\mathcal {V}} }_{\imath (s)}^{-}\right) ^{2}\right| \\ +\left| \left( \overline{{\mathcal {V}} _{h}}_{_{\imath j}}\right) ^{2}-\left( \overline{ {\mathcal {V}} _{h}}_{_{\imath }}^{-}\right) ^{2}\right| \end{array} \right) \end{array} \right) ,\text { \ } \\ \text {where }\imath= & {} 1,2,3,...,\ell ,\text {and \ }j=1,2,3,...,m. \end{aligned}$$
Step-6: The following are the relative closeness indices calculated for all decision makers of the alternatives:
  $$\begin{aligned} RC(\alpha _{\imath j})=\frac{d(\alpha _{\imath j},{\mathcal {T}}^{+})}{d(\alpha _{\imath j},{\mathcal {T}} ^{-})+d(\alpha _{\imath j},{\mathcal {T}}^{+})}. \end{aligned}$$
Step-7: The alternatives may be ranked according to their desirability, and the choice with the lowest distance can be selected.

### Numerical example of the improved TOPSIS approach

**Figure 4:**
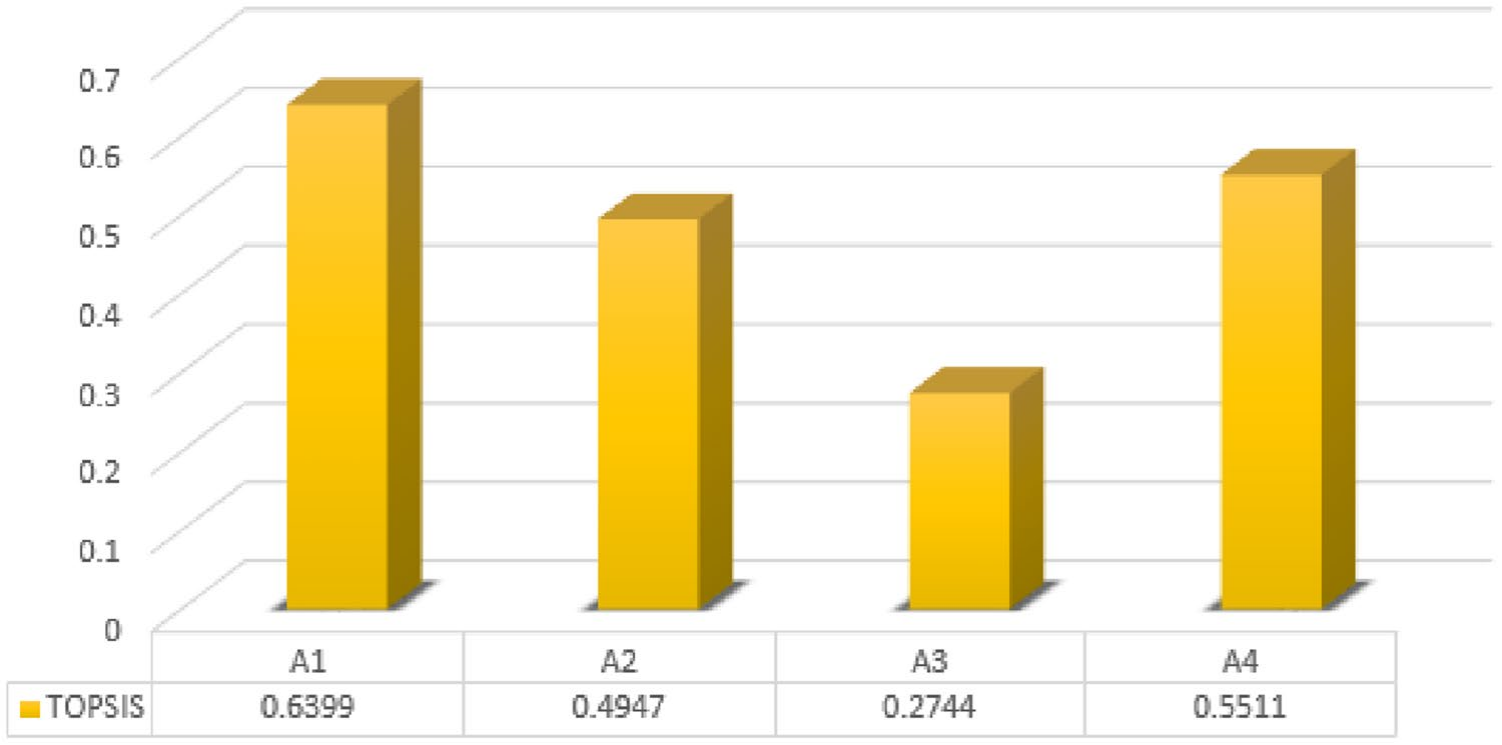
The schematic depiction of the ranking based on the improved TOPSIS approach.

A numerical example relevant to “green supplier selection in industrial systems” is provided to illustrate the effectiveness of the proposed approach as follows:

Step-1: The information of decision makers is displayed in the form of FHFRNs in Tables 2, 3, 4, 5.
Step-2: The distance between PIS is computed as follows:
  $$\begin{aligned} \begin{array}{llll} 0.2936 &{} 0.1399 &{} 0.0940 &{} 0.3029 \\ \end{array} \end{aligned}$$
  and similarly the the distance between NIS is calculated as follows:
  $$\begin{aligned} \begin{array}{llll} 0.1652 &{} 0.1429 &{} 0.2486 &{} 0.2467 \\ \end{array} \end{aligned}$$
Step-4: The following are the relative closeness indices for all decision-makers evaluating the alternatives:
  $$\begin{aligned} \begin{array}{llll} 0.6399 &{} 0.4947 &{} 0.2744 &{}0.5511 \\ \end{array} \end{aligned}$$
Step-5: According to the aforementioned ranking of alternatives visualized in Fig. 4, the $A_3$ has the shortest distance. Hence, $A_{3}$ is the best option.

## Concluding remarks and future recommendations

This research presented an enhanced model of the Fermatean hesitant fuzzy rough set, a novel hybrid structure of the Fermatean FSs, the HFSs, and the rough set for GSS in process industries^79–82^. The addition of RS theory makes this method more adaptable and efficient for modelling fuzzy systems and crucial DM under ambiguity. A variety of AOPs, including FHFRWA, FHFR ordered WA, and FHFR hybrid WA operators, is presented utilizing algebraic t-norm and t-conorm. In addition, the key properties of developed operators are elaborately discussed. This study presented an assessment approach for determining each potential supplier’s overall performance. The optimal selection may then be made based on the overall rating of the supplier. Using a suggested model can assist organisational decisions by employing a suggested approach towards selecting the most appropriate supplier. The possible uses of the MCDM approach for determining the best decision were illustrated utilising numerical examples. The suggested techniques and the improved FHFR-VKOR and TOPSIS methods are used to compare the final ranking and best decision for selecting the green suppliers in process industries. The comparison demonstrated the capability, superiority, and trustworthiness of the suggested approaches.

In the future, the presented approach may be extended to solve MAGDM problems involving generalised aggregated information with applications in machine learning, artificial intelligence, medical diagnostics, and DM challenges.

## Data Availability

All data generated or analysed during this study are included in this manuscript.

## References

[1] Aguezzoul, A. Overview on supplier selection of goods versus 3PL selection. In:* 2011 4th International Conference on Logistics*. (IEEE, 2011). 248–253.

[2] Quan J, Zeng B, Liu D (2018). Green supplier selection for process industries using weighted grey incidence decision model. Complexity.

[3] Singh R, Rajput H, Chaturvedi V, Vimal J (2012). Supplier selection by technique of order preference by similarity to ideal solution (TOPSIS) method for automotive industry. Int. J. Adv. Technol. Eng. Res..

[4] Chan FT, Kumar N, Tiwari MK, Lau HC, Choy K (2008). Global supplier selection: A fuzzy-AHP approach. Int. J. Prod. Res..

[5] Cheraghi, S.H., Dadashzadeh, M. and Subramanian, M. Critical success factors for supplier selection: An update. J. Appl. Bus. Res. (JABR) 20(2) (2004).

[6] Ho W, Xu X, Dey PK (2010). Multi-criteria decision making approaches for supplier evaluation and selection: A literature review. Eur. J. Oper. Res..

[7] Kumar A, Jain V, Kumar S (2014). A comprehensive environment friendly approach for supplier selection. Omega.

[8] Ali MI (2018). Another view on q-rung orthopair fuzzy sets. Int. J. Intell. Syst..

[9] Dubois, D.J. Fuzzy Sets and Systems: Theory and Applications Vol. 144. (Academic press, 1980).

[10] Khan MJ, Kumam P, Shutaywi M (2021). Knowledge measure for the q-rung orthopair fuzzy sets. Int. J. Intell. Syst..

[11] Liu D, Huang A (2020). Consensus reaching process for fuzzy behavioral TOPSIS method with probabilistic linguistic q-rung orthopair fuzzy set based on correlation measure. Int. J. Intell. Syst..

[12] Mendel JM (2007). Advances in type-2 fuzzy sets and systems. Inf. Sci..

[13] Zadeh LA (2011). A note on Z-numbers. Inf. Sci..

[14] Zadeh LA (1965). Fuzzy sets. Inf. Control.

[15] Zhan J, Alcantud JCR (2019). A survey of parameter reduction of soft sets and corresponding algorithms. Artif. Intell. Rev..

[16] Rodríguez RM, Martínez L, Torra V, Xu ZS, Herrera F (2014). Hesitant fuzzy sets: State of the art and future directions. Int. J. Intell. Syst..

[17] Atanassov, K.T. Intuitionistic fuzzy sets. In:* Intuitionistic Fuzzy Sets*. (Physica, 1999). 1–137.

[18] Yager RR (2013). Pythagorean membership grades in multicriteria decision making. IEEE Trans. Fuzzy Syst..

[19] Torra V (2010). Hesitant fuzzy sets. Int. J. Intell. Syst..

[20] Pawlak Z (1982). Rough sets. Int. J. Comput. Inf. Sci..

[21] Attaullah S, Ashraf S, Rehman N, Khan A, Park C (2022). A decision making algorithm for wind power plant based on q-rung orthopair hesitant fuzzy rough aggregation information and TOPSIS. AIMS Math..

[22] Attaullah S, Ashraf S, Rehman N, Alsalman H, Gumaei AH (2022). A decision-making framework using q-rung orthopair probabilistic hesitant fuzzy rough aggregation information for the drug selection to treat COVID-19. Complexity.

[23] Attaullah, *et al.* A wind power plant site selection algorithm based on q-rung orthopair hesitant fuzzy rough Einstein aggregation information. *Sci. Rep.* **12**(1), 1–25 (2022). 10.1038/s41598-022-09323-5 PMC8971469 35361827

[24] Attaullah, Ashraf, S., Rehman, N. and Khan, A. q-Rung orthopair probabilistic hesitant fuzzy rough aggregation information and their application in decision making.* Int. J. Fuzzy Syst.* 1–14 (2022).

[25] Wu H (2021). An improved calibration and uncertainty analysis approach using a multicriteria sequential algorithm for hydrological modeling. Sci. Rep..

[26] Wang CN, Nguyen NAT, Dang TT (2022). Offshore wind power station (OWPS) site selection using a two-stage MCDM-based spherical fuzzy set approach. Sci. Rep..

[27] Abdel-Basset M, Mohamed M, Mostafa NN, El-Henawy IM, Abouhawwash M (2022). New multi-criteria decision-making technique based on neutrosophic axiomatic design. Sci. Rep..

[28] Limberger, F., Rümpker, G., Lindenfeld, M. and Deckert, H. Development of a numerical modelling method to predict the seismic signals generated by wind farms. (2022). 10.1038/s41598-022-19799-w PMC9478089 36109555

[29] Stańczyk J, Kajewska-Szkudlarek J, Lipinski P, Rychlikowski P (2022). Improving short-term water demand forecasting using evolutionary algorithms. Sci. Rep..

[30] Eseoglu G, Yapsakli K, Tozan H, Vayvay O (2022). A novel fuzzy framework for technology selection of sustainable wastewater treatment plants based on TODIM methodology in developing urban areas. Sci. Rep..

[31] Huang Y, Li T, Luo C, Fujita H, Horng SJ (2017). Matrix-based dynamic updating rough fuzzy approximations for data mining. Knowl. Based Syst..

[32] Hu J, Li T, Luo C, Fujita H, Yang Y (2017). Incremental fuzzy cluster ensemble learning based on rough set theory. Knowl. Based Syst..

[33] Zhang J, Li T, Chen H (2014). Composite rough sets for dynamic data mining. Inf. Sci..

[34] Chen D, Yang Y, Dong Z (2016). An incremental algorithm for attribute reduction with variable precision rough sets. Appl. Soft Comput..

[35] El-Alfy ESM, Alshammari MA (2016). Towards scalable rough set based attribute subset selection for intrusion detection using parallel genetic algorithm in MapReduce. Simul. Model. Pract. Theory.

[36] Eskandari S, Javidi MM (2016). Online streaming feature selection using rough sets. Int. J. Approx. Reason..

[37] Pattaraintakorn P, Cercone N, Naruedomkul K (2006). Rule learning: Ordinal prediction based on rough sets and soft-computing. Appl. Math. Lett..

[38] Sanchis A, Segovia MJ, Gil JA, Heras A, Vilar JL (2007). Rough Sets and the role of the monetary policy in financial stability (macroeconomic problem) and the prediction of insolvency in insurance sector (microeconomic problem). Eur. J. Oper. Res..

[39] Valdés, J.J., Romero, E. and Gonzalez, R. Data and knowledge visualization with virtual reality spaces, neural networks and rough sets: Application to geophysical prospecting. In:* 2007 International Joint Conference on Neural Networks*. (IEEE, 2007). 160–165.

[40] Ni YC, Yang JG, Lv ZJ (2006). Raw cotton yarn Tenacity’s rule extraction based on rough set theory. Prog. Text. Sci. Technol..

[41] Chen ZC, Zhang F, Jiang DZ, Ni LL, Wang HY (2004). The filtering method for X-ray digital image of chest based on multi-resolution and rough set. Chin. J. Biomed. Eng..

[42] Pang FH, Pang ZL, Du RQ (2008). Assessment on rough-set theory for lake ecosystem health index. J. Biomath..

[43] Greco S, Matarazzo B, Slowinski R (2001). Rough sets theory for multicriteria decision analysis. Eur. J. Oper. Res..

[44] Zhu, Y. C., Xiong, W., Jing, Y. W. & Gao, Y. B. Design and realization of integrated classifier based on rough set. *Tongxin Xuebao (J. Commun.)* **27**(11), 63–67 (2006).

[45] Minghui WANG (2004). Study on the application of rough set in railway dispatching system. China Railw. Sci..

[46] Li WX, Cheng M, Li BY (2008). Extended dominance rough set theory’s application in food safety evaluation. Food Res. Dev..

[47] Liang ZA, Liu F, Zhao Q (2007). Application of rough-set theory and neural network at superfamily level in insect taxonomy. Acta Zootaxonomica Sin..

[48] Kuang LH, Xu LR, Liu BS, Yao JC (2006). A new method for choosing zonation indicators of mudflow danger degrees based on the rough set theory. J. Geomechan..

[49] Hu F, Huang JG, Chu FH (2008). Grey relation evaluation model of weapon system based on rough set. Acta Armamentarii.

[50] Dubois D, Prade H (1990). Rough fuzzy sets and fuzzy rough sets. Int. J. General Syst..

[51] Khan MA, Ashraf S, Abdullah S, Ghani F (2020). Applications of probabilistic hesitant fuzzy rough set in decision support system. Soft Comput..

[52] Yun SM, Lee SJ (2015). Intuitionistic fuzzy rough approximation operators. Int. J. Fuzzy Logic Intell. Syst..

[53] Zhan J, Malik HM, Akram M (2019). Novel decision-making algorithms based on intuitionistic fuzzy rough environment. Int. J. Mach. Learn. Cybern..

[54] Zhang C (2020). Classification rule mining algorithm combining intuitionistic fuzzy rough sets and genetic algorithm. Int. J. Fuzzy Syst..

[55] Hussain A, Ali MI, Mahmood T (2020). Pythagorean fuzzy soft rough sets and their applications in decision-making. J. Taibah Univ. Sci..

[56] Cornelis C, De Cock M, Kerre EE (2003). Intuitionistic fuzzy rough sets: At the crossroads of imperfect knowledge. Expert Syst..

[57] Jena SP, Ghosh SK, Tripathy BK (2002). Intuitionistic fuzzy rough sets. Notes Intuitionistic Fuzzy Sets.

[58] Feng F, Li C, Davvaz B, Ali MI (2010). Soft sets combined with fuzzy sets and rough sets: a tentative approach. Soft Comput..

[59] Feng F, Liu X, Leoreanu-Fotea V, Jun YB (2011). Soft sets and soft rough sets. Inf. Sci..

[60] Zhang, H., Shu, L. and Liao, S. Intuitionistic fuzzy soft rough set and its application in decision making. In:* Abstract and Applied Analysis* Vol. 2014. (Hindawi, 2014).

[61] Zhang H, Shu L, Liao S (2016). On interval-valued hesitant fuzzy rough approximation operators. Soft Comput..

[62] Zhou L, Wu WZ (2011). Characterization of rough set approximations in Atanassov intuitionistic fuzzy set theory. Comput. Math. Appl..

[63] Hussain A, Ali MI, Mahmood T, Munir M (2020). q-Rung orthopair fuzzy soft average aggregation operators and their application in multicriteria decision-making. Int. J. Intell. Syst..

[64] Pamucar D (2020). Normalized weighted geometric Dombi Bonferroni mean operator with interval grey numbers: Application in multicriteria decision making. Rep. Mech. Eng..

[65] Ashraf S, Abdullah S, Khan S (2020). Fuzzy decision support modeling for internet finance soft power evaluation based on sine trigonometric Pythagorean fuzzy information. J. Ambient Intell. Hum. Comput..

[66] Batool B, Ahmad M, Abdullah S, Ashraf S, Chinram R (2020). Entropy based pythagorean probabilistic hesitant fuzzy decision making technique and its application for fog-haze factor Assessment problem. Entropy.

[67] Khan AA (2019). Pythagorean fuzzy Dombi aggregation operators and their application in decision support system. Symmetry.

[68] Peng X, Dai J, Garg H (2018). Exponential operation and aggregation operator for q-rung orthopair fuzzy set and their decision-making method with a new score function. Int. J. Intell. Syst..

[69] Wang P, Wei G, Wang J, Lin R, Wei Y (2019). Dual hesitant q-rung orthopair fuzzy hamacher aggregation operators and their applications in scheme selection of construction project. Symmetry.

[70] Wang J, Wei G, Wei C, Wei Y (2019). Dual hesitant q-Rung Orthopair fuzzy Muirhead mean operators in multiple attribute decision making. IEEE Access.

[71] Wang Y, Shan Z, Huang L (2020). The extension of TOPSIS method for multi-attribute decision-making with q-Rung orthopair hesitant fuzzy sets. IEEE Access.

[72] Zhou L, Wu WZ (2008). On generalized intuitionistic fuzzy rough approximation operators. Inf. Sci..

[73] Yager RR (2016). Generalized orthopair fuzzy sets. IEEE Trans. Fuzzy Syst..

[74] Liu D, Peng D, Liu Z (2019). The distance measures between q-rung orthopair hesitant fuzzy sets and their application in multiple criteria decision making. Int. J. Intell. Syst..

[75] Chinram R, Hussain A, Mahmood T, Ali MI (2021). EDAS method for multi-criteria group decision making based on intuitionistic fuzzy rough aggregation operators. IEEE Access.

[76] Hwang, C.L. and Yoon, K., Methods for multiple attribute decision making. In:* Multiple Attribute Decision Making*. (Springer, 1981). 58–191.

[77] Hsu PF, Hsu MG (2008). Optimizing the information outsourcing practices of primary care medical organizations using entropy and TOPSIS. Quality & Quantity.

[78] Tzeng, G. H. & Huang, J. J. Multiple Attribute Decision Making: Methods and Applications. (CRC Press, 2011).

[79] Peng X, Yang Y (2015). Some results for Pythagorean fuzzy sets. Int. J. Intell. Syst..

[80] Peng X, Liu L (2019). Information measures for q-rung orthopair fuzzy sets. Int. J. Intell. Syst..

[81] Senapati T, Yager RR (2020). Fermatean fuzzy sets. J. Ambient Intell. Hum. Comput..

[82] Zhang X, Xu Z (2014). Extension of TOPSIS to multiple criteria decision making with Pythagorean fuzzy sets. Int. J. Intell. Syst..

